# DFCP1-containing ER microdomains mediate lysosomal membrane repair

**DOI:** 10.1038/s41556-026-02062-z

**Published:** 2026-09-15

**Authors:** Maja Radulovic, Sascha Pust, Athanasios Kournoutis, Di Chen, M. Isabel Giner, Qijian Liang, Santosh Phuyal, Thomas J. Böddeker, Katy McCarron, Eric Herrmann, Kevin Rose, Sebastian W. Schultz, Andreas Brech, James H. Hurley, Claudio Bussi, Luis Bonet-Ponce, Maximiliano G. Gutierrez, Camilla Raiborg, Harald Stenmark

**Affiliations:** 1 Centre for Cancer Cell Reprogramming, Institute of Clinical Medicine, Faculty of Medicine, University of Oslo, Montebello, Norway; 2 Institute for Cancer Research, Oslo University Hospital, Montebello, Norway; 3 Host Pathogen Interactions in Tuberculosis laboratory, The Francis Crick Institute, London, UK; 4 Department of Biological Chemistry and Pharmacology, The Ohio State University, Columbus, OH USA; 5 Department of Neurology, The Neuroscience Research Institute, College of Medicine, The Ohio State University Wexner Medical Center, Columbus, OH USA; 6 School of Biological Sciences, Nanyang Technological University, Singapore, Singapore; 7 Precision Immunotherapy Alliance, Institute of Clinical Medicine, Faculty of Medicine, University of Oslo, Montebello, Norway; 8 Institute of Molecular Biology and Biochemistry, Charité – Universitätsmedizin Berlin, Berlin, Germany; 9 Department of Molecular and Cellular Biology, University of California, Berkeley, Berkeley, CA USA; 10 Institute for Digital Molecular Analytics and Science (IDMxS), Nanyang Technological University, Singapore, Singapore

**Keywords:** Lysosomes, Macroautophagy

## Abstract

Lysosomal membrane integrity is essential for preserving cellular homeostasis in response to different stressors. Upon lysosomal membrane permeabilization, cells activate several mechanisms for lysosomal membrane repair, including ESCRT proteins, phosphatidylinositol 4-phosphate (PI4P)-dependent lipid transfer from the endoplasmic reticulum (ER) and conjugation of ATG8 family proteins to single membranes (CASM). The interplay between these pathways and the regulation of the lipid transfer machinery remain incompletely understood. Here we show that phosphatidylinositol 3-phosphate (PI3P)-containing ER domains play a major role in lysosomal membrane repair. PI3P is formed on lysosome-proximal ER domains by the phosphatidylinositol 3-kinase PIK3C3/VPS34 in response to membrane damage, and inhibition or depletion of PIK3C3 inhibits lysosome repair. Mechanistically, the ATPase DFCP1/ZFYVE1 accumulates on lysosome-proximal ER domains by its PI3P binding, triggered by Ca^2+^ efflux from lysosomes and requiring the ULK1 kinase complex and ER proteins of the VAP family. Downstream of CASM, PI4P, ESCRTs and PI3P, DFCP1 promotes focal accumulation of the lipid channel VPS13C on ER domains proximal to damaged lysosomes to promote their repair. The function and dynamics of DFCP1 depend on its ability to bind and hydrolyse ATP, and absence of DFCP1 compromises cellular resistance to vacuolar damage induced by *Listeria* *monocytogenes*. We conclude that DFCP1 mediates concentration of the ER-associated lipid transport machinery at damaged lysosomes to promote their sealing in response to Ca^2+^ flux and PIK3C3 activation.

## Abstract (web summary)

Radulovic et al. show that phosphatidylinositol 3-phosphate (PI3P), produced by the phosphatidylinositol 3-kinase PIK3C3, is formed on ER domains after lysosome damage. ER domain PI3P helps to recruit the ATPase DFCP1, which promotes lysosome repair.

## Main

Lysosomes are the cell’s main stations for degradation of macromolecules, particles and pathogens, and they also serve as hubs for metabolic regulation^1,2^. As lysosomes contain lipases and other molecules that can potentially cause membrane damage, and as they accumulate endocytosed molecules, particles and pathogens, their membrane is prone to damage. Such damage can activate cell death and inflammatory signalling by several alternative pathways, and protective cellular mechanisms have therefore evolved to limit the harmful consequences of lysosome destabilization, namely lysosomal membrane repair and degradation of damaged lysosomes by lysophagy^3,4^.

Although discovered relatively recently^5,6^, lysosomal membrane repair has gained considerable attention as a notable mechanism in cellular homeostasis with relevance to infections, ageing, neurodegenerative diseases and cancer^3,4^. To date, several pathways of lysosome repair have been identified^5–11^, including the endosomal sorting complex required for transport (ESCRT)^5,6^, conjugation of ATG8 proteins to single membranes (CASM)^10–13^, and phosphatidylinositol 4-phosphate (PI4P) triggered phospholipid and cholesterol transport from the endoplasmic reticulum (ER)^14,15^. Phospholipid and cholesterol transport is mediated by ORP family proteins that form ER–lysosome contact sites through binding to VAP proteins in the ER membrane and PI4P in the lysosome membrane^14,15^. The lipid channel proteins ATG2A/B, VPS13C and LYVAC are also recruited to mediate high-capacity phospholipid transport from ER to damaged or osmotically stressed lysosomes^14,16,17^.

While ESCRT recruitment, CASM and the involvement of PI4P and ER–lysosome contact sites in lysosomal repair have been well documented, their interrelationships have been incompletely characterized, and the mechanisms that control lipid transport are not known. In the present study we show that phosphatidylinositol 3-phosphate (PI3P), produced by the phosphatidylinositol 3-kinase PIK3C3, is formed on characteristic ER domains after lysosome damage, triggered by Ca^2+^ release. ER-associated PI3P causes recruitment of the double FYVE domain containing protein DFCP1/ZFYVE1, which has previously been implicated in autophagosome biogenesis^18^. This facilitates lysosome repair after both chemically and pathogen-induced damage by promoting focal accumulation of the ER-associated lipid transport machinery at damaged lysosomes.

## Results

### PI3P is formed on ER domains in response to lysosome damage

**Extended Data Fig. 1:**
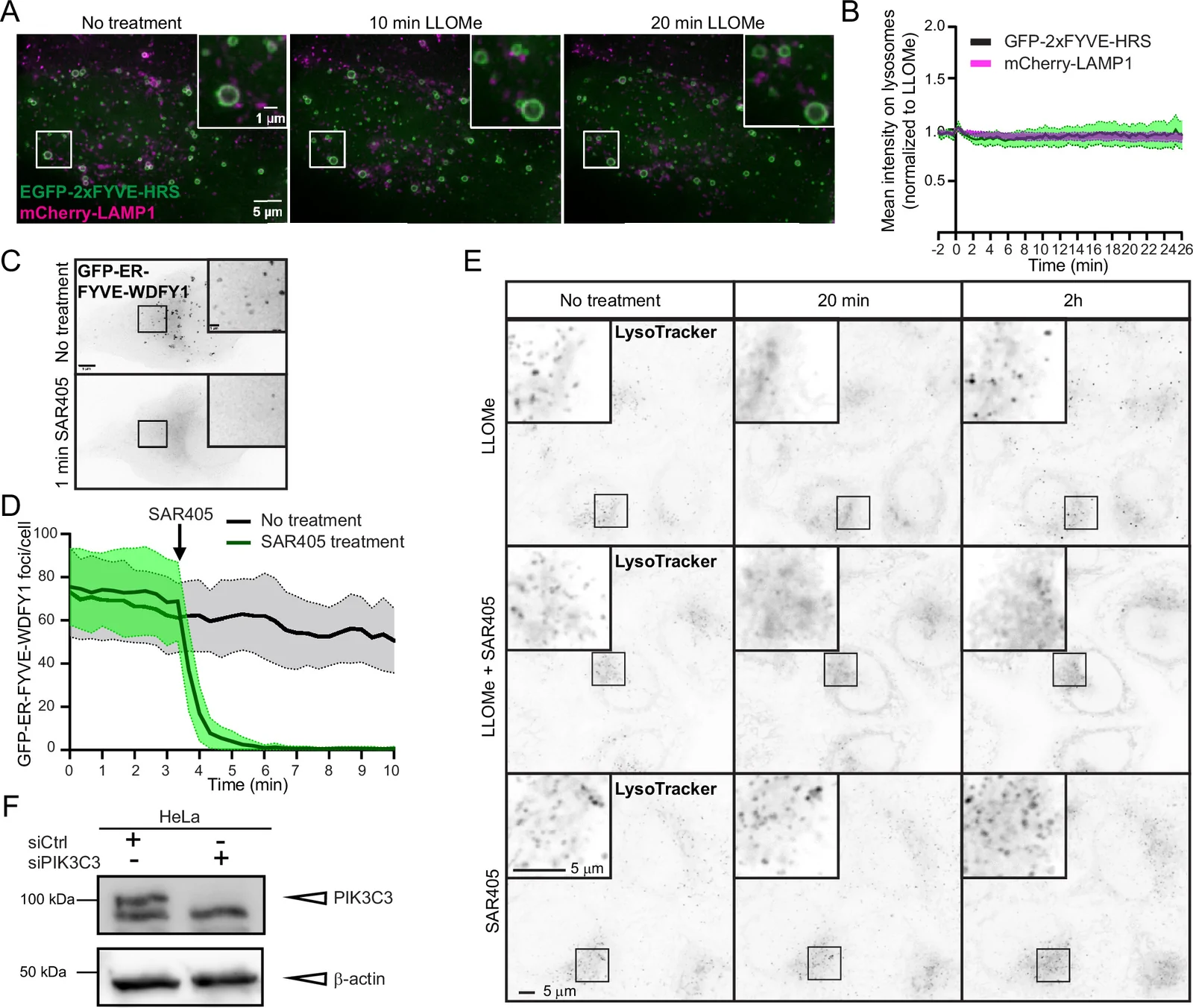
PI3P at the ER-associated sites is PIK3C3-dependent. **a)** Representative video stills of HeLa cells transiently expressing the PI3P probe EGFP-2xFYVE-HRS and the lysosomal marker mCherry–LAMP1 following treatment with 250 µM LLOMe. Upon induction of lysosomal damage, no increase in EGFP-2xFYVE-HRS signal was observed on mCherry–LAMP1–positive vesicles compared with untreated cells. Images are representative of >20 cells from *n* = 3 independent experiments. Scale bar, 5 µm, scale bar inset: 1 µm. **b)** Quantification graph of live-cell imaging experiment from A), representing mean intensity of GFP-2xFYVE-HRS or mCherry–LAMP1 probe normalized to LLOMe. Error bars correspond to mean ± 95% confidence intervals from 14 videos. >45 cells from *n* = 3 independent experiments were analysed. **c)** Representative video stills and **d)** quantification graph of the live-cell imaging experiments illustrating sensitivity of ER- associated PI3P (GFP-ER-FYVE-WDFY1 probe) to SAR405 treatment. Scale bar, 5 µm, scale bar inset: 1 µm. Error bars denote mean ± SD from more than 16 videos from *n* = 3 independent experiments, > 40 cells were analysed per experiment for each condition. **e)** Representative video stills of the LysoTracker repair assay shown in Fig. 1f from *n* = 3 independent experiments. Treatment with 250 µM LLOMe or 1 µM SAR405 cause LysoTracker fluorescence recovery. However, upon LLOMe + SAR405 treatment HeLa cells fail to repair damaged lysosomes. Scale bar, 5 µm, scale bar inset: 5 µm. **f)** Knockdown efficiency of siRNA depletion of PIK3C3 in HeLa cells as detected by western blot corresponding to Fig. 1g, h. β-actin used as a loading control. Source numerical data and unprocessed blots are available in source data. Source data

Because phosphoinositides are important regulators of both cellular membrane dynamics^19^ and formation of membrane contact sites^20^ (MCSs), we hypothesized that phosphoinositides at the ER might play a role in the lysosomal damage response. PI4P is formed rapidly on lysosome membranes by PI4K2A in response to their damage^14,15^. We addressed the possibility that PI3P production on the ER could also play a role in the lysosomal damage response although no increase in PI3P has been detected with the standard PI3P probe 2xFYVE^Hrs^ (refs. ^15,21^) (Extended Data Fig. 1a,b and Supplementary Video 1). As this probe requires a certain density of PI3P molecules in the membrane to detect PI3P, it will not detect low PI3P levels. Previous studies have shown that very low levels of phosphoinositides, including PI3P, can be detected by probes that contain an additional membrane targeting domain that promotes coincidence detection^18,22^. To investigate whether PI3P is formed on ER membranes after lysosome damage, we therefore used an ER-targeted PI3P probe, GFP-ER-FYVE^WDFY1^, which has previously been used to detect PI3P formed on ER domains that promote autophagosome biogenesis^18^.

**Fig. 1:**
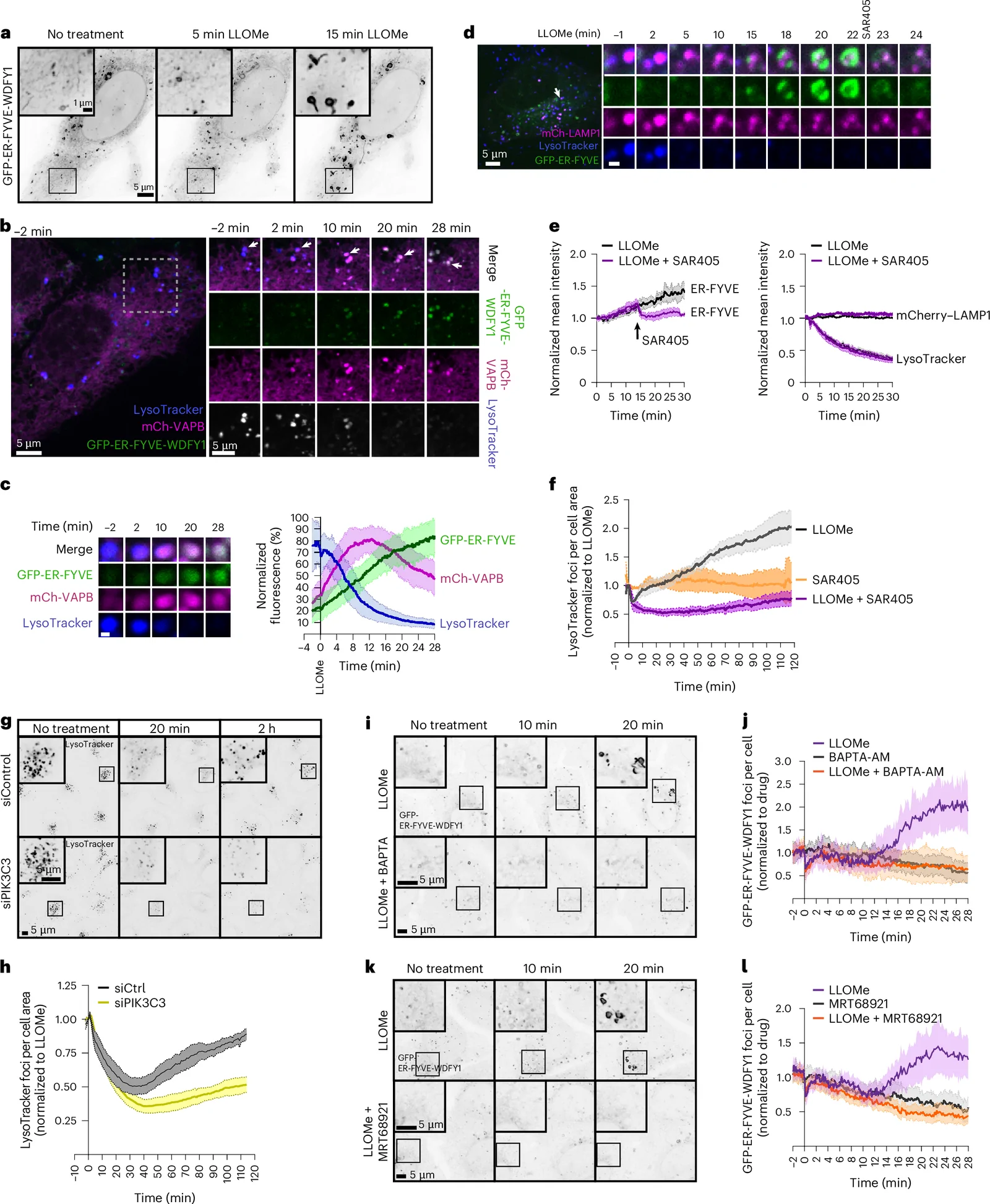
Lysosomal damage induces Ca^2+^- and ULK1-dependent activation of PIK3C3, and formation of PI3P on ER domains is required for lysosome repair. **a**, Video stills from live-cell imaging experiments showing recruitment of the GFP-ER-FYVE-WDFY1 probe following 250 µM LLOMe treatment. Data are representative of *n* = 3 independent experiments, >100 cells per experiment were imaged. Scale bar, 5 µm; scale bar inset, 1 µm. **b**, Representative video stills from live-cell imaging experiments illustrating recruitment and co-occurrence of stably expressed GFP-ER-FYVE-WDFY1 probe (green) with the ER marker mCherry–VAPB (magenta) following 250 µM LLOMe treatment and imaged every 10 s for 30 min. Cells were pre-treated with 75 nM LysoTracker Deep Red for 30 min to monitor lysosome damage (grey). Scale bar, 5 µm; scale bar inset, 5 µm. **c**, Representative gallery of a single tracked lysosome from **b** (arrow) and quantification of GFP-ER-FYVE-WDFY1 probe (green), mCherry–VAPB (magenta) and LysoTracker (blue) mean intensities, showing PI3P accumulation relative to the drop in LysoTracker signal. Shaded error bands denote mean ± s.d. of 55 tracked lysosomes, from *n* = 4 independent experiments. Scale bar, 500 nm. **d**, Video stills from tracked lysosomes from a live-cell imaging experiment show loss of LLOMe-induced formation of PI3P at ER domains (GFP-ER-FYVE-WDFY1 probe) upon treatment with 1 µM SAR405. Scale bar, 5 µm; scale bar inset, 500 nm. **e**, Graphs represent quantifications of U2OS cells transiently expressing GFP-ER-FYVE-WDFY1 probe treated with LLOMe and SAR405, as shown in **d**. Shaded error bands denote mean ± s.e.m. from *n* = 4 independent experiments, >18 cells per condition. **f**, The graph represents quantifications of LysoTracker-positive foci per cell. HeLa cells were pre-treated with 75 nM LysoTracker Deep Red for 30 min before the addition of 250 µM LLOMe, 1 µM SAR405 or LLOMe and SAR405. Shaded error bands denote mean ± s.e.m. from *n* = 3 independent experiments, >120 cells were analysed per experiment. **g**,**h**, Representative images (**g**) and quantification graph (**h**) of HeLa siControl and siPIK3C3 cells pre-treated with 75 nM LysoTracker Deep Red for 30 min before the addition of 250 µM LLOMe (0 min). Cells were imaged every 1 min for 120 min. While the decrease in the number of LysoTracker foci was quickly recovered in HeLa siControl cells treated with LLOMe, there was a severe impairment in lysosomal repair in HeLa siPIK3C3 cells. Shaded error bands denote mean ± s.e.m. from *n* = 4 independent experiments, >130 cells were analysed per experiment. Scale bar, 5 µm; scale bar inset, 5 µm. **i**,**j**, Representative images (**i**) and quantification graph (**j**) of U2OS cells stably expressing GFP-ER-FYVE-WDFY1 probe. Cells were pre-treated with 15 µM BAPTA-AM for 45 min before LLOMe addition at time 0 and imaged every 10 s for 30 min. While LLOMe treatment alone induced rapid recruitment and accumulation of the GFP-ER-FYVE-WDFY1 probe, BAPTA-AM pre-treatment largely abolished this response, resulting in a marked reduction in probe accumulation following lysosomal damage. Shaded error bands denote mean ± 95% CI from 12 live-cell imaging videos for LLOMe and BAPTA-AM and from 10 live-cell imaging videos for LLOMe + BAPTA-AM conditions, >77 cells were analysed per condition from *n* = 4 independent experiments. Scale bar, 5 µm; scale bar inset, 5 µm. **k**,**l**, Representative images (**k**) and quantification graph (**l**) of U2OS cells stably expressing GFP-ER-FYVE-WDFY1 probe and treated with 250 µM LLOMe, 1 µM MRT68921 and MRT68921 + LLOMe. Cells were imaged every 10 s for 30 min. Cells treated with LLOMe alone displayed robust recruitment and accumulation of the GFP-ER-FYVE-WDFY1 probe. In contrast, co-treatment with MRT68921 markedly impaired probe accumulation following lysosomal damage, resulting in a substantially reduced GFP-ER-FYVE-WDFY1 number of foci compared with the LLOMe-only condition. Shaded error bands denote mean ± 95% CI from 21 live-cell imaging videos per condition, >169 cells were analysed from *n* = 7 independent experiments. Scale bar, 5 µm; scale bar inset, 5 µm. Source numerical data are available in source data. Source data

Live-cell imaging of untreated U2OS cells expressing this probe showed faint labelling in reticular ER-like structures as well as on vesicle-like structures, probably reflecting some recruitment to PI3P on PI3P-rich endosomes. Of note, however, when a low concentration (250 µM) of LLOMe (a compound that causes rapid and specific damage of lysosome membranes^23,24^) was added to the cells, tubular and ring-shaped membrane domains were strongly detected with the PI3P probe within minutes after lysosome damage (Fig. 1a and Supplementary Video 2). To clarify the origin of these domains, we performed triple-labelling live-fluorescence microscopy with U2OS cells stably expressing GFP-ER-FYVE^WDFY1^, and coexpressing the ER marker mCherry–VAPB, and the lysosome marker LysoTracker. This imaging showed that the PI3P probe emerged on VAP-B-containing ER domains shortly after addition of LLOMe to the cells, and in multiple events, these domains seemed to wrap around lysosomes (Fig. 1b and Supplementary Video 3).

To analyse in detail the relationship between membrane damage, PI3P formation and ER mobilization, we monitored single lysosomes by live microscopy and correlated loss of LysoTracker fluorescence (a sign of membrane damage) with acquisition of GFP-ER-FYVE^WDFY1^ and mCherry–VAPB. Loss of LysoTracker fluorescence was accompanied by acute accumulation of GFP-ER-FYVE^WDFY1^ and mCherry–VAPB (Fig. 1c and Supplementary Video 4). These results demonstrate a tight temporal coupling between lysosome damage, PI3P formation and ER mobilization.

### PI3P formed by PIK3C3 is required for repair of damaged lysosomes

Although PI3P can be formed on cellular membranes by several mechanisms, the phosphatidylinositol 3-kinase PIK3C3/VPS34 is responsible for production of most of this phosphoinositide^19,25^. Under basal conditions, formation of PI3P on ER domains was completely abolished by a specific inhibitor of PIK3C3, SAR405 (ref. ^26^) (Extended Data Fig. 1c,d and Supplementary Video 5). More notably, SAR405 strongly reduced the LLOMe-induced formation of PI3P on ER domains (Fig. 1d,e and Supplementary Video 6). These findings indicate that PI3P is rapidly generated by PIK3C3 at ER sites in response to lysosomal damage.

The accumulation of PI3P on lysosome-contacting ER domains in response to lysosome damage raised the question whether PI3P is required for lysosome repair. To address this, we used the well-established LysoTracker recovery assay, which measures regain of LysoTracker fluorescence in lysosomes following recovery after LLOMe-induced lysosome damage^5,6^. As expected, lysosomes in HeLa cells rapidly lost lysosomal LysoTracker fluorescence after addition of LLOMe, which was rapidly recovered as a sign of lysosome repair. Of note, however, when cells were treated with LLOMe and the PIK3C3 inhibitor SAR405, LysoTracker recovery was strongly inhibited (Fig. 1f, Extended Data Fig. 1e and Supplementary Video 7a–c). A similar result was obtained when PIK3C3 was depleted by siRNA (Fig. 1g,h and Extended Data Fig. 1f). This indicates that PIK3C3 is required for lysosomal membrane repair.

### PIK3C3 is activated by Ca^2+^ and ULK1 in response to lysosome damage

The above results raised the question of how PIK3C3 is activated upon lysosome damage. Previous studies have shown that Ca^2+^ leakage from damaged lysosomes is a major trigger of the lysosomal damage response^5,7,14^, and we therefore hypothesized that Ca^2+^ released from damaged lysosomes might trigger PIK3C3 activation. To address this, we treated cells expressing the GFP-ER-FYVE^WDFY1^ probe with the Ca^2+^ chelator BAPTA-AM and monitored its effect after LLOMe treatment. As shown in Fig. 1i,j, GFP-ER-FYVE^WDFY1^ recruitment in response to LLOMe was strongly inhibited by BAPTA-AM, indicating that Ca^2+^ is indeed a trigger of PIK3C3 activation upon lysosome damage. During autophagy induction, PIK3C3 is activated by the ULK1 kinase complex downstream of Ca^2+^ release from the ER^27–29^, and we therefore asked whether ULK1 is involved in the lysosomal damage response. Notably, the specific ULK1 inhibitor MRT68921 strongly inhibited GFP-ER-FYVE^WDFY1^ recruitment upon LLOMe treatment (Fig. 1k,l), and we therefore propose that Ca^2+^ release from damaged lysosomes triggers ULK1 to activate PIK3C3 on ER membranes.

### DFCP1-containing ER domains respond to lysosomal membrane damage

**Fig. 2:**
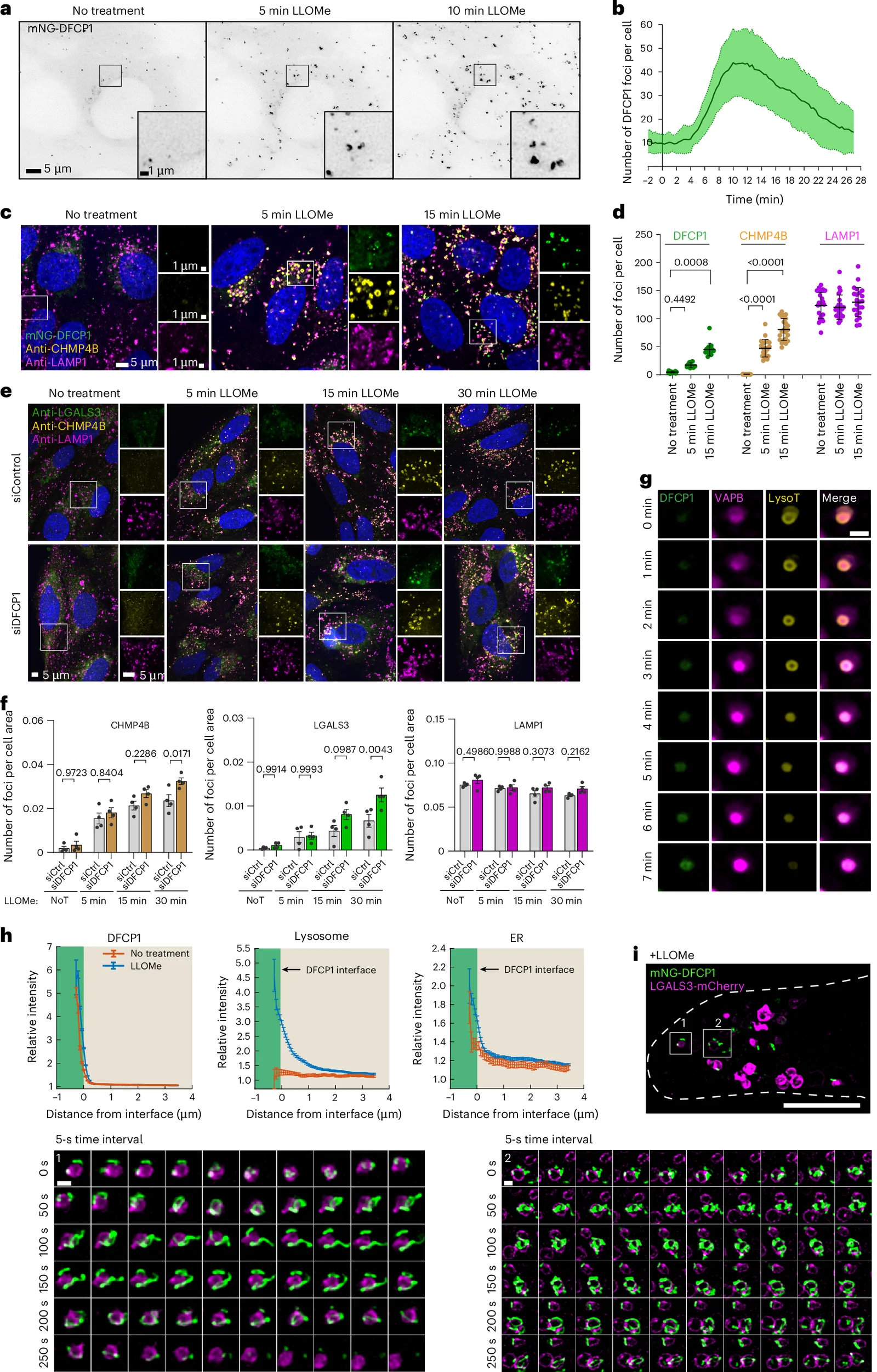
DFCP1-containing ER microdomains respond to lysosomal membrane damage. **a**, Representative video stills of a live-cell imaging experiment illustrating accumulation of mNG-DFCP1 foci upon 250 µM LLOMe treatment. Data representative of *n* = 3 independent experiments, >120 cells per experiment. Scale bar, 5 µm; scale bar inset, 1 µm. **b**, Quantification graph of mNG-DFCP1 foci per cell after LLOMe treatment. Shaded error bands denote mean ± s.d. from 20 live-cell imaging videos, >25 cells were analysed per experiment from *n* = 3 independent experiments. **c**, Representative fluorescence micrographs of U2OS-DFCP1-KO cells stably expressing mNG-DFCP1 treated with 250 μM LLOMe or an equal volume of dimethylsulfoxide (DMSO) (No treatment) for 5 and 15 min before fixation and immunostained with anti-CHMP4B (yellow) and anti-LAMP1 (magenta). Nuclei are stained with Hoechst (blue). Scale bar, 5 µm; scale bar inset, 1 µm. **d**, Quantification graph showing number of DFCP1, CHMP4B and LAMP1-positive foci per cell at different time points as illustrated in **c**. Every dot represents number of foci per cell per image. Error bars denote mean ± s.d. from 16–24 individual images from *n* = 5 independent experiments, >20 cells per experiment were analysed. Significant differences within the same group are shown, one-way analysis of variance (ANOVA) with Tukey’s multiple-comparisons test. **e**, Representative fluorescence micrographs of U2OS cells transfected with either siControl or siDFCP1 and treated with LLOMe or an equivalent volume of DMSO (No treatment) for 5, 15 and 30 min before fixation. Cells were immunostained with anti-CHMP4B (yellow), anti-LGALS3 (green) and anti-LAMP1 (magenta). Nuclei are stained with Hoechst (blue). Scale bar, 5 µm; scale bar inset, 5 µm. **f**, Quantification graph of data shown in **e**. DFCP1 depletion did not significantly affect the number of CHMP4B- or LGALS3-positive foci at early time points following lysosomal damage. The number of LAMP1-positive foci per cell area remained unchanged across all treatments and conditions. Scale bar, 5 µm; scale bar inset, 5 µm. Error bars denote mean ± s.e.m. from *n* = 4 independent experiments, >400 cells per condition per time point were analysed. Two-way ANOVA with Šidák’s multiple-comparisons test. **g**, Representative video stills of a live-cell imaging experiment illustrate response and accumulation of DFCP1 (green) positive foci to mCherry–VAPB (magenta) positive structures upon lysosome damage as indicated by the loss of LysoTracker fluorescence (yellow). Data representative of *n* = 3 independent live-cell imaging experiments, >70 cells per experiment were imaged. Scale bar, 1 µm. **h**, Normalized intensity of DFCP1, lysosomes and ER as a function of distance from the DFCP1 interface averaged over *n* = 1,400 puncta for LLOMe-treated and *n* = 390 puncta for control cells. LAMP1 (lysosome) and ER signal intensity within and around DFCP1 puncta increases upon LLOMe treatment compared with the control (no treatment) cells. Error bars denote standard error of the median from *n* = 3. **i**, Representative Lattice-SIM imaging of an LLOMe-treated cell expressing mNG-DFCP1 (green) and LGALS3–mCherry (magenta). Cell overview highlighting damaged lysosomes (boxes 1 and 2) (top). Time-lapse montages (5-s intervals) of the indicated regions showing the dynamic, partial envelopment of damaged lysosomes by DFCP1 (bottom). Images are representative of *n* = 3 independent experiments. Scale bar, 10 µm; scale bar insets, 1 µm. Source numerical data are available in source data. Source data

**Extended Data Fig. 2:**
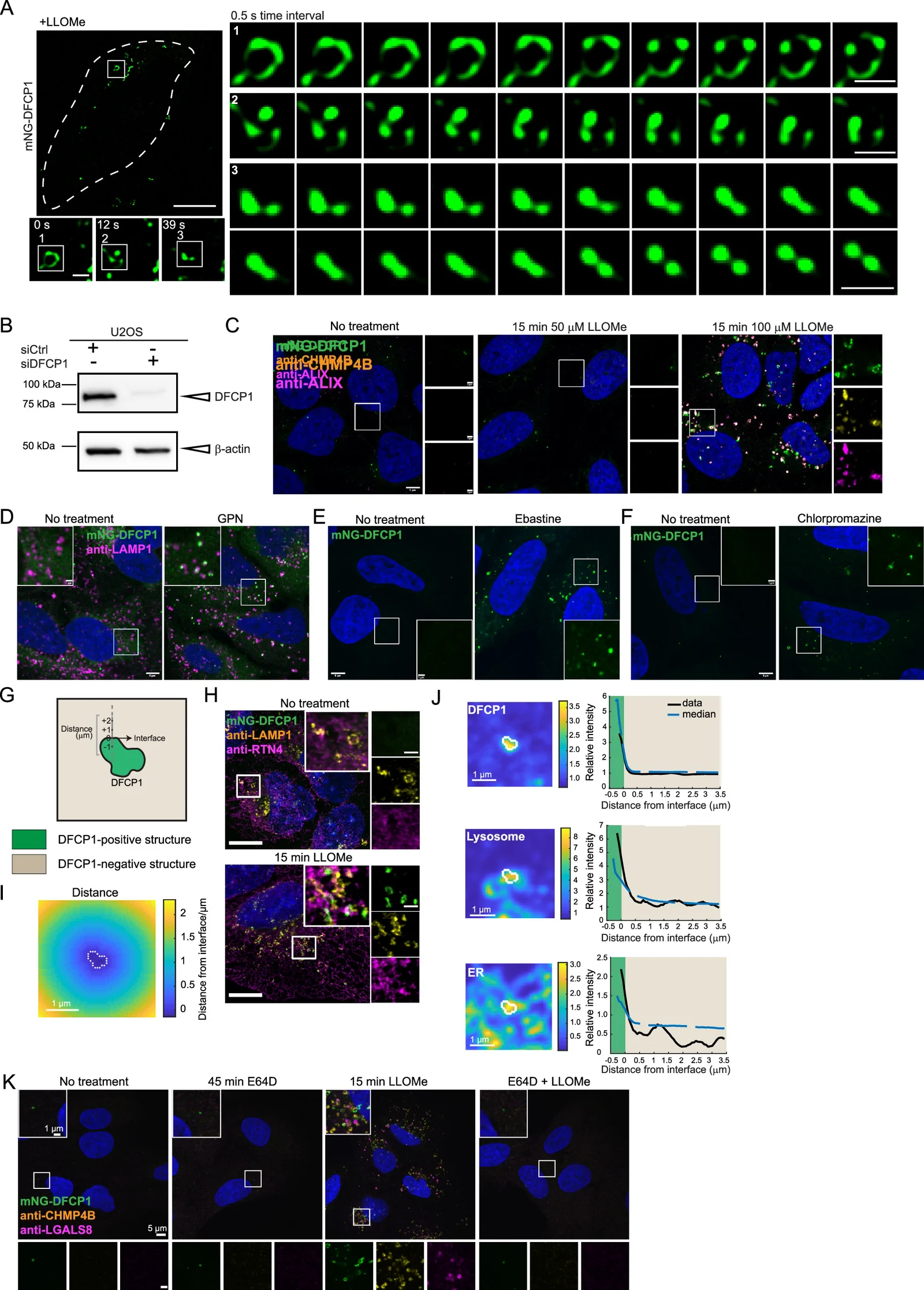
DFCP1 responds to different damaging reagents. **a)** Live-cell imaging of U2OS-DFCP1-KO cells stably expressing mNG-DFCP1 following LLOMe treatment. Selected time points (0-39 s) showing representative events (1–3). Events represent time series (0.5 s interval) illustrating rapid morphological transitions. Single z-plane shown. Images are representative of *n* = 3 independent experiments. Scale bar, 10 µm, scale bar insets: 1 µm. **b)** Knockdown efficiency of siRNA depletion of DFCP1 in U2OS cells as detected by western blot corresponding to Fig. 2e. β-actin used as a loading control. **c)** Representative fluorescence micrographs of U2OS-DFCP1-KO cells stably expressing mNG-DFCP1. After treatment with 50 µM LLOMe, 100 µM LLOMe or DMSO (No treatment) cells were fixed and immunostained with anti-CHMP4B (yellow) and anti-ALIX (magenta). Nuclei are stained with Hoechst (blue). Images are representative of *n* = 3 independent experiments. Scale bar, 5 µm, scale bar insets: 1 µm. **d)** Representative fluorescence images from *n* = 3 independent experiments of U2OS-mNG-DFCP1 cells treated with 250 µM glycyl-L-phenylalanine 2-naphthylamide (GPN) for 15 min and fixed. Cells were immunostained with anti-LAMP1 (magenta). Nuclei are stained with Hoechst (blue). Scale bar, 5 µm, scale bar inset: 1 µm. **e)** Representative fluorescence images from *n* = 3 independent experiments of U2OS-mNG-DFCP1 cells treated with 15 µM ebastine for 2.5 h and fixed. mNG-DFCP1 (green) and Hoechst (blue) are shown. Scale bar, 5 µm, scale bar inset: 1 µm. **f)** Representative fluorescence images from *n* = 3 independent experiments of U2OS-mNG-DFCP1 cells treated with 20 µM chlorpromazine for 2.5 h and fixed. mNG-DFCP1 (green) and Hoechst (blue) are shown. Scale bar, 5 µm, scale bar inset: 1 µm. **g)** Schematic illustration of quantification strategy used in Fig. 2h. Briefly, co-localization between mNG-DFCP1 and ER or lysosomes was evaluated by calculating the average intensity profiles of DFCP1, ER, and lysosome signals as a function of their distance from the DFCP1 puncta boundary. Radial intensity profiles were generated, with distances defined such that 0 corresponds to the punctum interface, negative values correspond to positions inside the punctum, and positive values to positions outside. **h)** Representative fluorescence images of U2OS-mNG-DFCP1 cells treated or not with 250 µM LLOMe for 15 min and fixed. Cells were immunostained with anti-LAMP1 (yellow) and anti-Reticulon-4 (magenta). Related to Fig. 2h. Scale bar, 10 µm, scale bar inset: 2 µm. **i)** Distance map for the exemplary DFCP1 punctum. Related to Fig. 2h. **j)** Representative image of a DFCP1 punctum and of the same location in the lysosome (LAMP1) and ER (Reticulon-4) channels. The graphs show the corresponding intensity as a function of distance from the DFCP1 interface compared with the median. Intensity normalized by the mean distance of each channel at a given distance from the cell nucleus. **k)** Representative fluorescence micrographs of U2OS-DFCP1-KO cells stably expressing mNG-DFCP1, treated with 250 µM LLOMe for 15 min, 50 µM E64D for 45 min, or with E64D and LLOMe as indicated. Cells were fixed and immunostained with anti-CHMP4B (yellow) and anti-LGALS8 (magenta). Nuclei were stained with Hoechst (blue). Cells treated with LLOMe show increased recruitment of CHMP4B, LGALS8 and DFCP1 when compared with DMSO-treated control (No treatment). Cells treated with E64D alone show no response, and E64D pre-treatment abrogates the effect of LLOMe on CHMP4B, LGALS8 and DFCP1 recruitment. Data represent >60-80 cells per condition. Scale bar, 5 µm, scale bar inset: 1 µm. Unprocessed blots are available in source data. Source data

A protein that has been previously shown to control the dynamics of ER domains is DFCP1, an ATPase that accumulates in characteristic ER domains, omegasomes, in response to amino acid starvation^18,30^. DFCP1 contains an ER-targeting domain and two non-canonical FYVE domains, which bind weakly but specifically to PI3P^31^. To probe whether DFCP1 responds to lysosome damage, we treated U2OS cells with LLOMe and used live-fluorescence microscopy to monitor the dynamics of monomeric NeonGreen (mNG)-tagged DFCP1 (ref. ^30^). At a low (250 µM) LLOMe concentration, we observed a noteworthy redistribution of mNG-DFCP1 from discrete puncta into larger structures that displayed highly dynamic behaviour, including rapid morphological rearrangements and recurrent fusion and fission events captured by fast live-cell imaging (Fig. 2a,b, Supplementary Video 8 and Extended Data Fig. 2a).

The recruitment dynamics were compared with those of the ESCRT protein CHMP4B and found to be within a similar time scale of a few minutes after LLOMe addition, indicating that DFCP1-containing ER domains respond rapidly to lysosome damage (Fig. 2c,d). However, depletion of DFCP1 did not affect CHMP4B recruitment at either 5 or 15 min following lysosomal damage, suggesting that DFCP1 does not play a major role in the initial membrane resilience response after damage (Fig. 2e,f and Extended Data Fig. 2b). The DFCP1 response occurred at LLOMe concentrations as low as 100 µM (Extended Data Fig. 2c) and was also observed with other compounds that cause lysosome damage, including the lysosomotropic agent glycyl-L-phenylalanine 2-naphthylamide (GPN; Extended Data Fig. 2d), the amphiphilic antihistamine ebastine (Extended Data Fig. 2e) and the antipsychotic drug chlorpromazine (Extended Data Fig. 2f). This indicates that DFCP1-containing ER domains respond dynamically to several types of stressors. In addition, DFCP1 response to lysosomal damage coincided spatially with the VAPB-positive ER domains and occurred in regions where LysoTracker fluorescence was lost, confirming that DFCP1 accumulation is tightly coupled to sites of lysosomal damage (Fig. 2g).

The colocalization of mNG-DFCP1 with ER and lysosomes was analysed by measuring the average intensity profile of DFCP1, ER and lysosomes as a function of distance from the interface of DFCP1 puncta (Extended Data Fig. 2g,h). Intensities were normalized to reveal variations from locally expected intensity, taking the cell geometry and distance from the nucleus into account (Methods and Extended Data Fig. 2i). Registered DFCP1 punctum together with the corresponding intensity profile across its interface, as well as the surrounding ER and lysosomes with their respective intensity profile relative to the DFCP1 punctum interface, are shown in Extended Data Fig. 2j. Quantitative analysis revealed weak colocalization of DFCP1 with both the ER and lysosomes in untreated cells. Following LLOMe treatment, colocalization between DFCP1 and the ER increased to approximately twofold relative to control cells. Notably, colocalization with lysosomes increased more prominently, reaching approximately threefold enrichment at the DFCP1 puncta interface and up to 4.6-fold enrichment at the centre of larger DFCP1-positive structures (Fig. 2h). Together, these analyses confirm persistent association of DFCP1 with ER membranes under all conditions while demonstrating enhanced accumulation of ER-associated DFCP1 at damaged lysosomes following LLOMe treatment.

By lattice structured illumination live-cell microscopy we monitored the distribution of DFCP1-positive ER microdomains relative to lysosomes in LLOMe-treated cells, using LGALS3 as a marker of damaged lysosomes. This analysis showed that the DFCP1-positive ER microdomains did not wrap around entire lysosomes. Instead, they encircled parts of the damaged lysosomes in a highly dynamic manner (Fig. 2i and Supplementary Video 9). In addition, preventing LLOMe-induced lysosomal membrane damage by inhibiting its cathepsin C-dependent activation with E64D^24^ completely abolished DFCP1 accumulation, demonstrating that DFCP1 specifically responds to lysosomal damage (Extended Data Fig. 2k).

### ER binding and VAP family proteins are required for DFCP1 response to lysosome damage

**Fig. 3:**
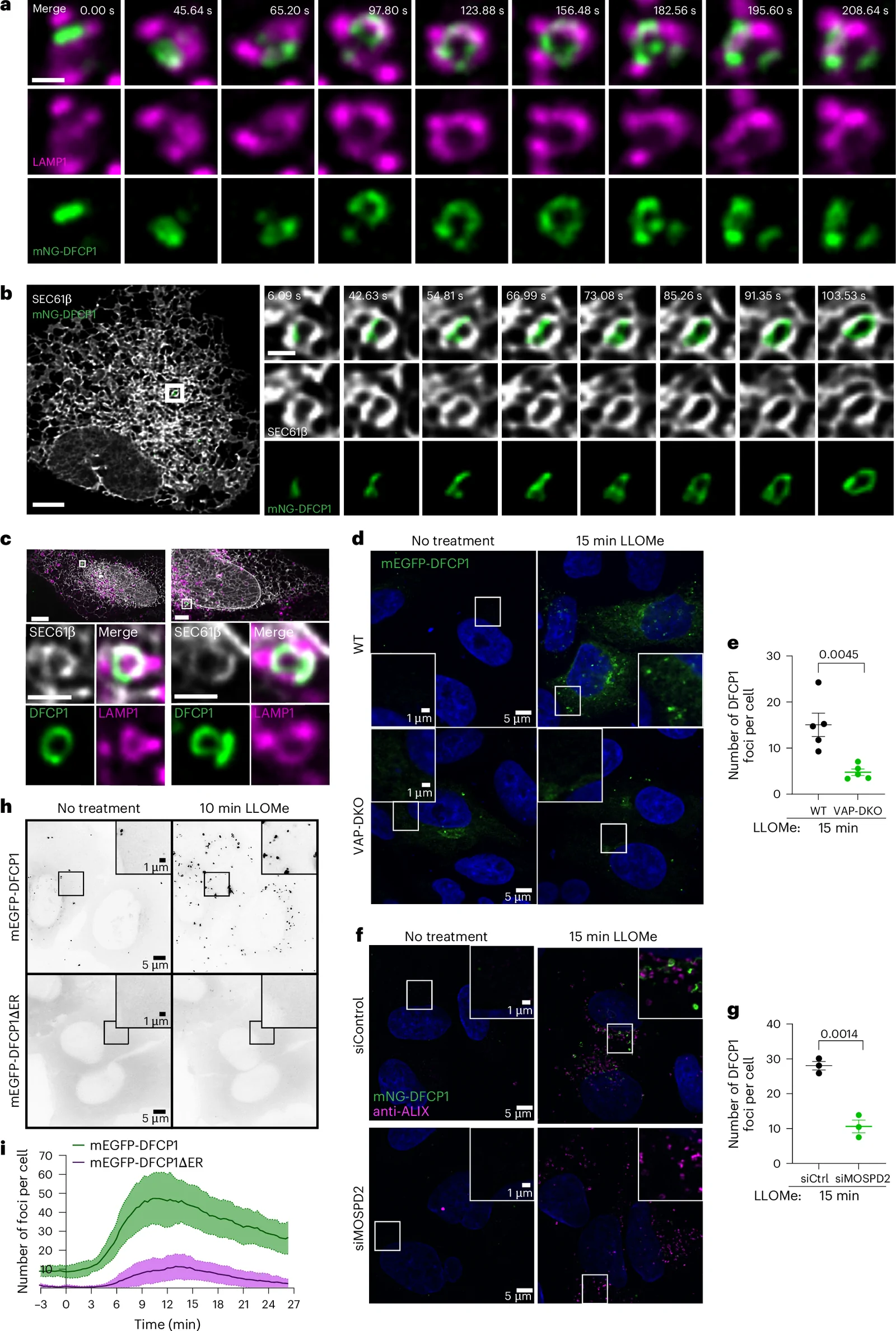
ER binding and VAP family proteins are required for DFCP1 response to lysosome damage. **a**, Representative live-cell images of U2OS-DFCP1-KO cells stably expressing mNG-DFCP1 transfected with LAMP1-HaloTag for 24 h. The ligand was added at a concentration of 50 nM for 10 min, followed by LLOMe treatment. The time interval between frames in the video is 6.52 s. Images are representative of *n* = 3 independent experiments. Scale bar, 1 µm. **b**, Representative live-cell imaging gallery of U2OS-DFCP1-KO cells stably expressing mNG-DFCP1 transfected with HaloTag-SEC61β for 24 h from *n* = 3 independent experiments. The ligand was added at a concentration of 50 nM for 5 min. Imaging started right after the addition of LLOMe. The time interval between frames is 6.09 s. For SEC61β the look-up table used was JDM_Greys g = 1.25. Scale bar, 7 µm; scale bar insets, 1 µm. **c**, Representative live-cell images of U2OS-DFCP1-KO cells stably expressing mNG-DFCP1 transfected with LAMP1-HaloTag and HaloTag-SEC61β indicating their co-occurrence 10 min post-LLOMe treatment from *n* = 3 independent experiments. Scale bar, 7 µm; scale bar insets, 1 µm. **d**, Representative fluorescence micrographs of HeLa WT or VAP-DKO cells transiently expressing mEGFP-DFCP1 illustrating reduced accumulation of DFCP1 foci in VAP-DKO cells 15 min post-treatment with 250 µM LLOMe when compared with control (no treatment). Scale bar, 5 µm; scale bar inset, 1 µm. **e**, The quantification graph of DFCP1-positive foci per cell in WT and VAP-DKO cells after 15 min of LLOMe treatment shows significant reduction in DFCP1 response to damage in the absence of VAP-A/B. Every dot represents average of DFCP1 foci per cell per experiment. Error bars denote mean ± s.d. from *n* = 5 independent experiments, >70 cells per experiment were analysed. Unpaired two-sided *t*-test. **f**, U2OS-DFCP1-KO cells stably expressing mNG-DFCP1 were co-transfected with siRNAs targeting MOSPD2 or with non-targeting siRNA control (siControl). At 48 h post-transfection, lysosomes were damaged with 250 μM LLOMe or not with DMSO (no treatment) for 15 min and fixed. Cells were immunostained with anti-ALIX (magenta). Scale bar, 5 µm; scale bar inset, 1 µm. **g**, Quantification graph of data shown in **f** indicating significant decrease in the number of DFCP1 foci per cell in siMOSPD2 when compared with siCtrl at 15 min post-LLOMe treatment. Every data point represents mean foci per cell per experiment. Error bars denote mean ± s.d. from *n* = 3 independent experiments, >50 cells per experiment per condition were analysed. Unpaired two-sided *t*-test. **h**, representative video stills of U2OS-DFCP1-KO cells stably expressing mEGFP-DFCP1 and a mutant DFCP1ΔER that lacks membrane targeting domain. Mutant that lacks ER-targeting fails to respond to LLOMe-induced lysosomal damage as indicated at 10 min post-treatment. Scale bar, 5 µm; scale bar inset, 1 µm. **i**, The graph showing quantification of DFCP1-full-length (green) or DFCP1ΔER (magenta) foci per cell over 30 min live-cell imaging experiment. Shaded error bands denote mean ± s.d. from 28 live-cell imaging videos, >25 cells per condition per experiment were analysed from *n* = 3 independent experiments. Source numerical data are available in source data. Source data

**Extended Data Fig. 3:**
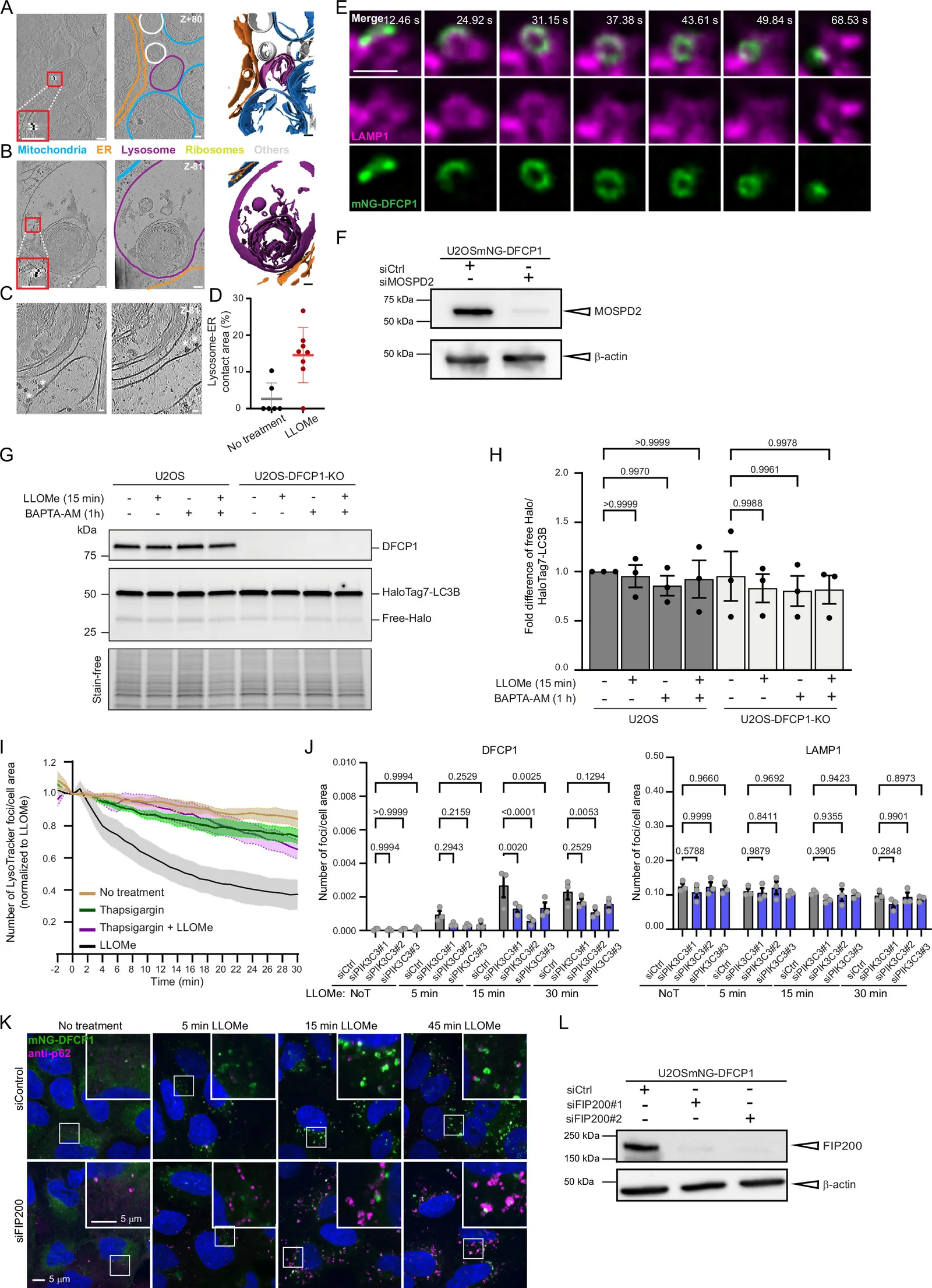
DFCP1 accumulation upon lysosome damage is dependent on Ca^2+^, ULK1 and PI3P. **a)** Representative cryo-electron tomogram and corresponding 3D segmentation of lysosomes in U2OS cells. The red box highlights the 15 nm gold fiducial used for lysosome identification. Scale bar, 100 nm. **b)** Representative cryo-electron tomogram and segmentation of lysosomes in U2OS-mNG-DFCP1 cells treated with LLOMe for 20 min. The white asterisks in the segmentation mark the position of membrane associated ribosomes as an ER marker. Cells were incubated for 4 h following washing steps and overnight incubation with 15-nm gold nanoparticles conjugated to bovine serum albumin to visualize lysosomes (indicated in red box). Scale bar, 100 nm. **c)** Close up of the lysosome-ER contact site shown in B. The white asterisks show the position of membrane attached to ribosomes. Scale bar, 25 nm. **d)** The quantification graph of the percentage area of lysosome-ER contacts sites based on the tomograms. In total 6 WT lysosomes and 8 LLOMe-treated U2OS-mNG-DFCP1 lysosomes were analysed. Error bars denote mean ± SD. **e)** Representative live-cell images of U2OS-DFCP1- KO cells stably expressing mNG-DFCP1 transfected with LAMP1-HaloTag for 24 h. The ligand was added at a concentration of 50 nM for 10 min, followed by LLOMe treatment. Video stills show mNG-DFCP1 interacting with lysosome. Additional example corresponding to Fig. 3a. The time interval between frames in the video is 6.23 s. Scale bar, 1 µm. **f)** Knockdown efficiency of siRNA depletion of MOSPD2 in U2OS-mNG-DFCP1 cells as detected by western blot corresponding to Fig. 3f, g. β-actin used as a loading control. **g)** In-gel fluorescence detection assay and immunoblotting of total cell lysates from wild-type and U2OS-DFCP1-KO cells stably expressing HaloTag7-LC3B. Cells were pulse-labelled for 30 min with 100 nM of tetramethylrhodamine (TMR)-conjugated ligand in nutrient-rich medium and treated with LLOMe, BAPTA, or both for the indicated times. **h)** Quantification of the in-gel fluorescence signal shown in **c**). Error bars denote mean ± SEM from *n* = 3 independent experiments. Statistical significance was assessed by one-way ANOVA with Tukey’s multiple-comparisons test. **i)** The quantification graph from live-cell imaging experiment shows the number of LysoTracker-positive foci per cell area normalized to the LLOMe. U2OS cells were pre-treated with 75 nM LysoTracker and 1.5 µM Thapsigargin for 30 min prior to induction of lysosomal damage with 250 µM LLOMe at time 0. Images were acquired every 1 min for 30 min. Error bars denote mean ± SEM from *n* = 4 independent experiments, >150 cells per condition were analysed. **j)** U2OS-DFCP1-KO cells stably expressing mNG-DFCP1 were transfected with three independent siRNAs targeting PIK3C3 (siPIK3C3 #1-#3) or a non-targeting control siRNA (siControl). 72 h post-transfection, cells were treated with 250 µM LLOMe or an equivalent volume of DMSO (NoT) for the indicated times, fixed, and immunostained for LAMP1. Graphs show quantification of DFCP1 foci and LAMP1-positive structures normalized to cell area. Error bars denote mean ± SEM from *n* = 3 independent experiments, >264 cells per condition per time point were analysed. Two-way ANOVA, Dunnett’s multiple-comparisons test. **k)** U2OS-DFCP1-KO cells stably expressing mNG-DFCP1 were transfected with siRNAs targeting FIP200 (siFIP200) or a non-targeting control siRNA (siControl). 72 h post-transfection, lysosomes were damaged with 250 µM LLOMe or DMSO (No treatment) for the indicated times, fixed, and immunostained for p62. Images correspond to data shown in Fig. 4n, mNG-DFCP1 shown in green and p62 in magenta. Scale bar, 5 µm, scale bar inset: 1 µm. **l)** Representative western blot showing knockdown efficiency of FIP200 in U2OS-mNG-DFCP1 cells corresponding to the experiments shown in Fig. 4n. β-actin used as a loading control. Source numerical data and unprocessed blots are available in source data. Source data

Cryo-electron tomography demonstrated that LLOMe-treated lysosomes localize in close proximity to the ER with a distance between the organelles of 20–25 nm, closely matching the length of the ATG2 and VPS13C lipid channels involved in ER-mediated lysosome repair (Extended Data Fig. 3a–d and Supplementary Table 1). Quantitative analyses of tomograms showed that ER–lysosome contacts increased after LLOMe treatment^15,24^. Upon induction of lysosomal damage, DFCP1 rapidly accumulated around LAMP1-positive structures, indicating that its response coincides with lysosome disruption (Fig. 3a, Extended Data Fig. 3e and Supplementary Video 10). Of note, the LLOMe-induced DFCP1-positive structures were also tightly associated with the ER (Fig. 3b and Supplementary Video 11), suggesting that DFCP1 accumulation during damage occurs at ER–lysosome MCSs (Fig. 3c). MCSs between lysosomes and ER contain ER-bound VAP proteins as an essential constituent^15^. To investigate whether VAP proteins are required for DFCP1 mobilization, we monitored mNG-DFCP1 recruitment after LLOMe treatment in HeLa cells lacking VAP-A and VAP-B^32^. Recruitment of mEGFP-DFCP1 was strongly reduced in the VAP-A-VAP-B double KO cells (Fig. 3d,e), and was also reduced by siRNA-mediated depletion of a third VAP family member, MOSPD2 (ref. ^33^) (Fig. 3f,g and Extended Data Fig. 3f). This indicates that VAP family proteins are required for DFCP1 mobilization in response to lysosomal damage. A DFCP1 construct lacking the ER-binding region, mEGFP-DFCP1ΔER, failed to respond after LLOMe treatment even in the presence of VAP family proteins (Fig. 3h,i and Supplementary Video 12), confirming that ER binding is required for the relocalization of DFCP1 after lysosome damage.

### Ca^2+^, ULK1 and PI3P trigger the DFCP1 response to lysosome damage

**Fig. 4:**
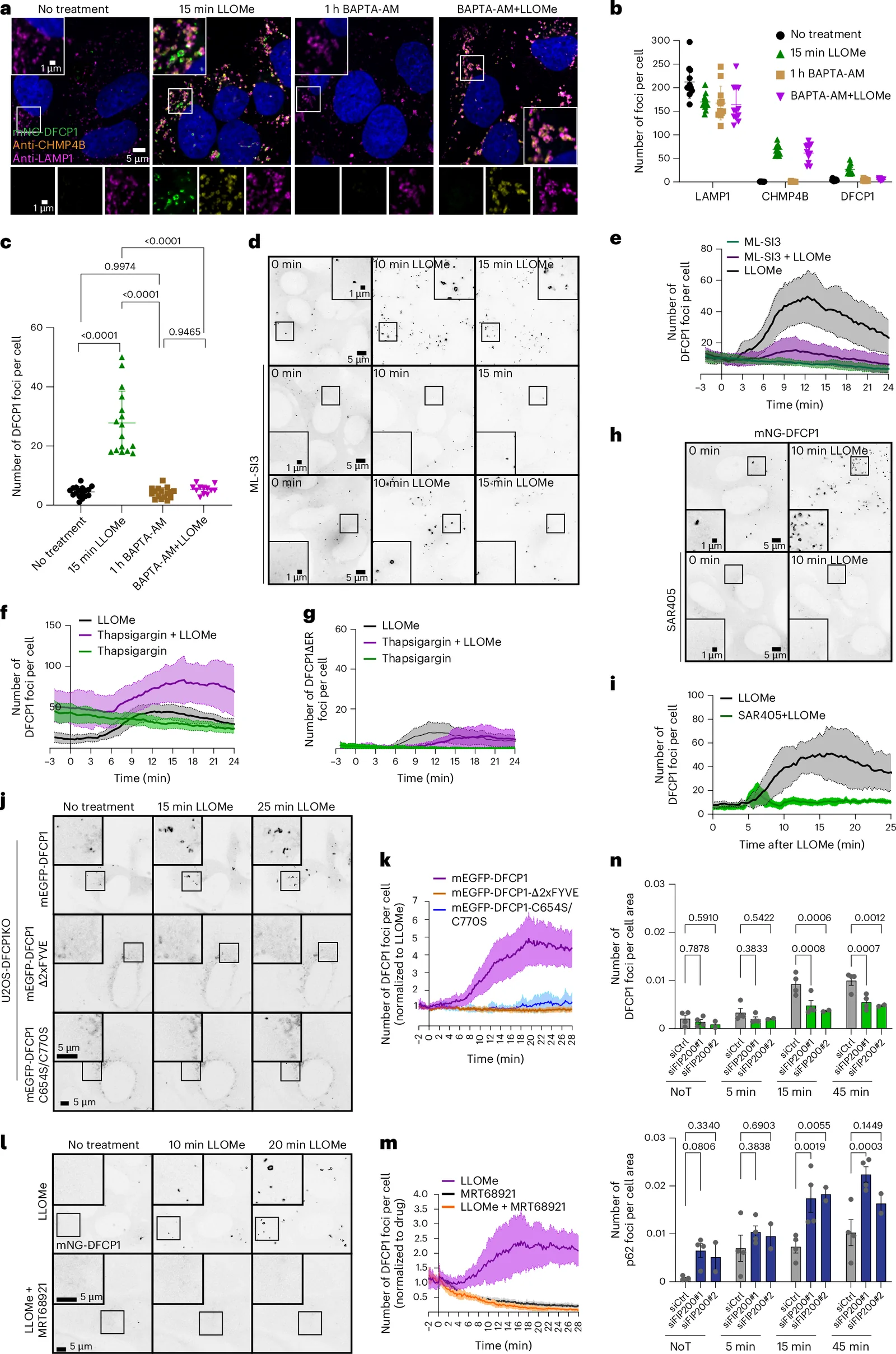
DFCP1 accumulation upon lysosome damage is dependent on Ca^2+^, ULK1 and PI3P. **a**, Representative fluorescence micrographs of U2OS-DFCP1-KO cells stably expressing mNG-DFCP1 treated with 250 µM LLOMe for 15 min, with 15 µM BAPTA-AM for 1 h, or with BAPTA-AM and LLOMe as indicated. Cells were fixed and immunostained with anti-CHMP4B (yellow) and anti-LAMP1 (magenta). Nuclei are stained with Hoechst (blue). Scale bar, 5 µm; scale bar inset, 1 µm. **b**, Quantification graph of data shown in **a** illustrating number of foci per cell for LAMP1, CHMP4B and DFCP1 upon different treatments as indicated. Every dot represents average foci per cell per image. Error bars denote mean ± s.d. from 12–17 images from *n* = 3 independent experiments, >20 cells per condition per experiment were analysed. **c**, Zoom in on quantification of DFCP1 foci per cell from **b** showing statistical difference between treatments from 12–17 images from *n* = 3 independent experiments. One-way ANOVA with Tukey’s multiple-comparisons test. **d**, Representative video stills of U2OS-mNG-DFCP1 cells treated with LLOMe, TRPML antagonist ML-SI3 for 30 min or pre-treated with ML-SI3 + LLOMe. Scale bar, 5 µm; scale bar inset, 1 µm. **e**, Quantification graph of data shown in **d**. LLOMe was added at 0 min. Shaded error bands denote mean ± s.d. from more than 14 live-cell imaging videos per condition, >20 cells per condition per experiment were analysed from *n* = 3 independent experiments. **f**, Quantification graph showing number of DFCP1 foci per cell in cells treated with 1.5 µM Thapsigargin for 30 min before the addition of LLOMe at time 0. Shaded error bands denote mean ± s.d. from 16 live-cell imaging videos per condition, >25 cells per condition per experiment were analysed from *n* = 3 independent experiments. **g**, U2OS-DFCP1-KO cells stably expressing a mutant that cannot bind to the ER-targeting domain DFCP1ΔER show no response to Thapsigargin, LLOMe or Thapsigargin + LLOMe treatment. Shaded error bands denote mean ± s.d. from more than 23 live-cell imaging videos per condition, >25 cells per condition per experiment were analysed from *n* = 3 independent experiments. **h**, Representative video stills of U2OS-mNG-DFCP1 cells illustrating no accumulation of DFCP1 upon treatment with 5 µM SAR405 + 10 min LLOMe. Scale bar, 5 µm; scale bar inset, 1 µm. **i**, Quantification graph of data shown in **h**. Shaded error bands denote mean ± s.d. from seven live-cell imaging videos per condition, >23 cells per condition per experiment were analysed from *n* = 3 independent experiments. **j**, Representative video stills of U2OS-DFCP1-KO cells transiently expressing mEGFP-DFCP1, mutant depleted for 2xFYVE domain (U2OS-DFCP1KO-mEGFP-DFCP1Δ2xFYVE), and point mutant C654S/C770S (U2OS-DFCP1KO-mEGFP-DFCP1C654S/C770S). Scale bar, 5 µm; scale bar inset, 5 µm. **k**, Quantification graph of data shown in **j**. Shaded error bands denote mean ± 95% CI from 52 cells for GFP-DFCP1-WT, 31 cells for U2OS-DFCP1KO-mEGFP-DFCP1Δ2xFYVE, and 36 cells for U2OS-DFCP1KO-mEGFP-DFCP1C654S/C770S from *n* = 5 independent experiments. **l**, Representative video stills of U2OS-DFCP1-KO cells stably expressing mNG-DFCP1 following treatment with LLOMe, 1 µM MRT68921, or LLOMe + MRT68921. Cells were imaged every 10 s for 30 min. Scale bar, 5 µm; scale bar inset, 5 µm. **m**, The quantification graph of data shown in **l**. Shaded error bands denote mean ± 95% CI from seven live-cell imaging videos for LLOMe, nine live-cell imaging videos for LLOMe + MRT68921, and 12 live-cell imaging videos for MRT68921; >57 cells per condition, from more than *n* = 4 independent experiments. **n**, U2OS-DFCP1-KO cells stably expressing mNG-DFCP1 were transfected with siRNAs against non-targeting control (siCtrl) and siFIP200 (#1 and #2 indicate two different siRNAs). At 72 h post-transfection cells were treated with DMSO (NoT) or LLOMe for 5, 15 and 45 min fixed and immunostained with anti-p62. Quantification graph represents number of DFCP1 or p62 foci per cell area. Error bars denote mean ± s.e.m. from *n* = 4 independent experiments for siCtrl and siFIP200#1, and *n* = 2 independent experiments for siFIP200#2, >50 cells per condition per time point per experiment were analysed. Two-way ANOVA with Dunnett’s multiple-comparisons test. Source numerical data are available in source data. Source data

Previous studies of autophagy have shown that DFCP1 responds to cytosolic Ca^2+^ (ref. ^34^), and we found that PI3P formation on ER membranes upon lysosome damage is activated by Ca^2+^ release. We therefore monitored whether chelation of cytosolic Ca^2+^ with BAPTA-AM^5^ would affect DFCP1 redistribution to LAMP1-containing lysosomes after LLOMe treatment. Indeed, treatment with BAPTA-AM completely abolished DFCP1 redistribution after LLOMe-induced lysosome damage (Fig. 4a–c). Of note, the ESCRT-III protein CHMP4B was recruited to damaged lysosomes in response to LLOMe even in the presence of BAPTA-AM, indicating that ESCRT recruitment is less sensitive to Ca^2+^ than DFCP1. To exclude the possibility that the effect of BAPTA-AM resulted from a general inhibition of autophagy^35^, we confirmed that BAPTA-AM treatment did not affect autophagic activity under these experimental conditions, indicating that its effect on DFCP1 redistribution is specific to lysosomal damage (Extended Data Fig. 3g,h).

The lysosomal Ca^2+^ channel TRPML1/MCOLN1 has been shown to respond to lysosome damage^5,36^, and we therefore monitored whether an inhibitor of this channel, ML-SI3 (ref. ^37^), would affect DFCP1 redistribution after LLOMe treatment. This was found to be the case (Fig. 4d,e and Supplementary Video 13), indicating that the Ca^2+^ flux that triggers DFCP1 recruitment after lysosome damage at least partially originates from TRPML1 channels.

DFCP1 has been found to form multiple puncta after incubation of cells with Thapsigargin, a compound that causes accumulation of cytosolic Ca^2+^ due to inhibition of the sarcoplasmic reticulum/ER Ca^2+^ ATPase^38,39^. Following lysosomal damage, cells co-treated with Thapsigargin and LLOMe exhibited increased lysosomal resilience compared with cells treated with LLOMe alone, as indicated by LysoTracker staining (Extended Data Fig. 3i), suggesting that elevated intracellular Ca^2+^ promotes lysosomal membrane resilience. Interestingly, even in the presence of Thapsigargin, LLOMe treatment caused a strong increase in DFCP1 profiles (Fig. 4f and Supplementary Video 14), supporting the notion that Ca^2+^ release from lysosomes triggers DFCP1 redistribution. A DFCP1 mutant lacking the ER-binding domain did not show any foci neither in response to Thapsigargin nor LLOMe (Fig. 4g and Supplementary Video 15). This shows that the DFCP1 foci triggered by Ca^2+^ and lysosome damage represent ER-bound DFCP1.

Because localization of DFCP1 to ER membranes during autophagy induction has been shown to require its binding to PI3P^18^, we also investigated LLOMe-induced DFCP1 redistribution in the absence and presence of the PIK3C3 inhibitor SAR405. Fluorescence microscopy showed that DFCP1 puncta were completely abolished in the presence of SAR405 (Fig. 4h,i and Supplementary Video 16). Likewise, DFCP1 constructs harbouring mutations in the FYVE domains that prevent PI3P binding^18^ or that completely lack both FYVE domains failed to respond to lysosome damage (Fig. 4j,k). Furthermore, transient depletion of PIK3C3 significantly reduced DFCP1 recruitment following LLOMe-induced damage (Extended Data Fig. 3j). These results indicate that PIK3C3-mediated formation of PI3P on ER domains causes DFCP1 recruitment in response to lysosome damage.

As we found that PIK3C3-mediated PI3P formation depended on ULK1, we addressed whether ULK1 is also required for DFCP1 mobilization after lysosome damage. Both the ULK1 inhibitor MRT68921 and siRNA against the ULK1 complex component FIP200 strongly inhibited DFCP1 mobilization after LLOMe treatment (Fig. 4l–n and Extended Data Fig. 3k,l). We conclude that the DFCP1 responds to lysosome is activated by PIK3C3 in response to Ca^2+^ efflux and ULK1 activation.

### DFCP1 mediates lysosomal membrane repair in an ATP-dependent manner

**Fig. 5:**
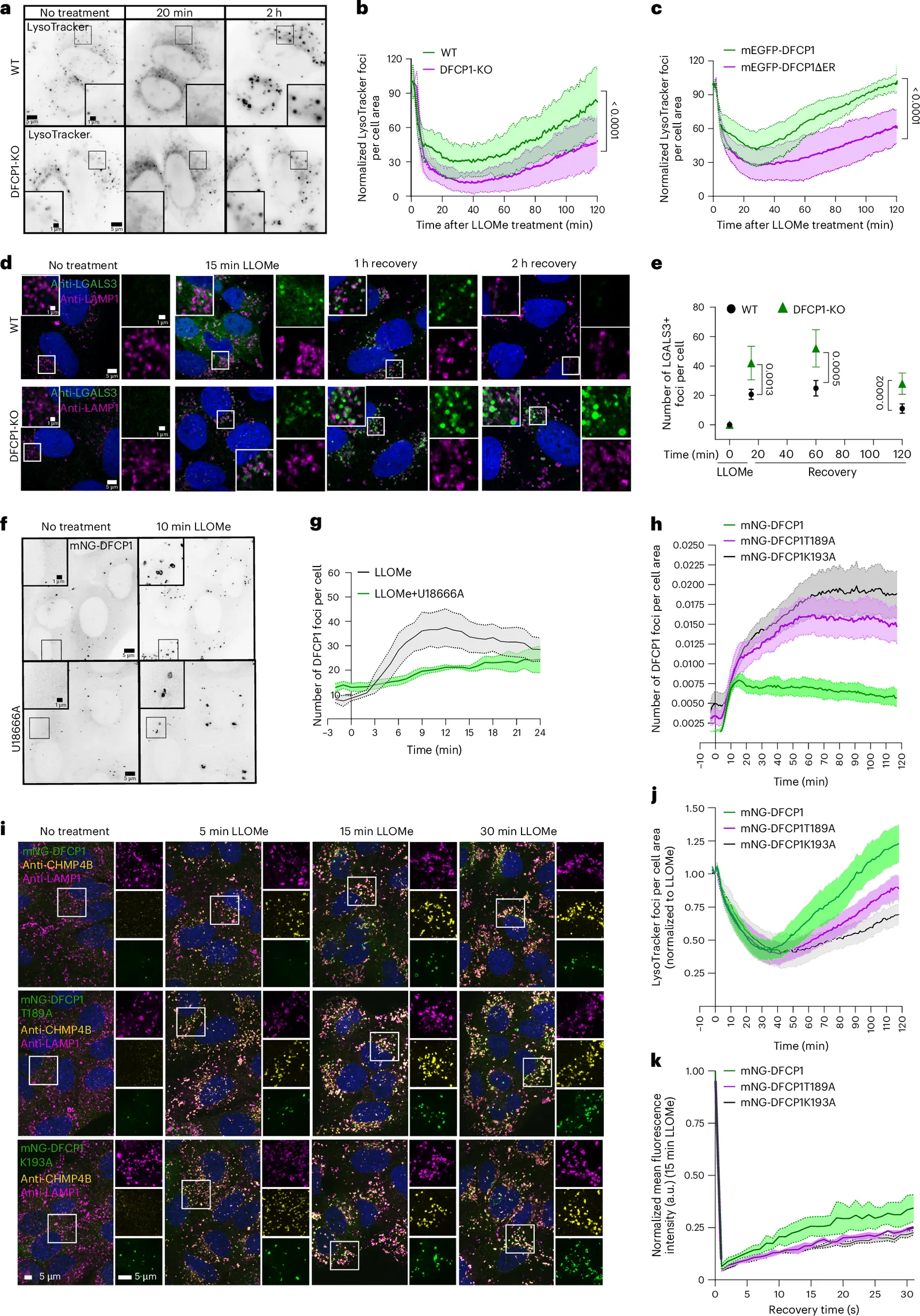
DFCP1 mediates lysosomal membrane repair in an ATPase-dependent manner. **a**, Representative video stills from live-cell imaging experiments of WT (U2OS) and DFCP1-KO cells monitoring LysoTracker recovery after induced lysosomal damage using 250 µM LLOMe. Cells were pre-treated with 75 nM LysoTracker Deep Red for 30 min before the addition of LLOMe. Scale bar, 5 µm; scale bar inset, 1 µm. **b**, The graph represents quantification of LysoTracker-positive foci per cell area. Shaded error bands denote mean ± s.d. from more than ten videos from more than *n* = 3 independent experiments, >100 cells per condition per experiment were analysed, two-way ANOVA. **c**, The graph represents the quantification of LysoTracker-positive foci per cell area of WT (U2OS-DFCP1-KO-mEGFP-DFCP1) and U2OS-DFCP1-KO-mEGFP-DFCP1ΔER cells that were pre-treated with 75 nM LysoTracker Deep Red for 30 min before the addition of LLOMe. Shaded error bands denote mean ± s.d. from more than 18 videos from more than *n* = 3 independent experiments, > 150 cells were analysed per experiment for each condition, two-way ANOVA. **d**, Representative fluorescence micrographs of U2OS (WT) or U2OS-DFCP1-KO cells treated with 250 μM LLOMe or an equal volume of DMSO (no treatment) for 15 min. Then LLOMe was washed off, and cells were left to recover for indicated time. After fixation cells were immunostained with anti-LGALS3 (green) and anti-LAMP1 (magenta). Nuclei are stained with Hoechst (blue). Scale bar, 5 µm; scale bar inset, 1 µm. **e**, Quantification graph of data shown in **c**. Error bars denote mean ± s.d. from 12 images from *n* = 3 independent experiments, >30 cells per condition per experiment were analysed, unpaired two-sided *t*-test. **f**, Representative video stills of U2OS-DFCP1-KO cells stably expressing mNG-DFCP1 indicate accumulation of DFCP1 after 10 min LLOMe treatment when compared with the control (no treatment). In the U18666A panel, cells were pre-incubated for 16 h with 2 µM NPC1 inhibitor U18666A and imaged. Scale bar, 5 µm; scale bar inset, 1 µm. **g**, The graph shows quantification of data presented in **f**. Shaded error bands denote mean ± s.d. from three live-cell imaging experiment per condition, >30 cells per condition per experiment were analysed from *n* = 2 independent experiments. **h**, The graph represents the dynamics of recruitment of U2OS-DFCP1-KO cells stably expressing mNG-DFCP1, or ATPase mutants mNG-DFCP1T189A and mNG-DFCP1K193A at different time points before and after LLOMe treatment. LLOMe was added at 0 min. Images were acquired every 1 min for 120 min. Shaded error bands denote mean ± s.e.m. from *n* = 4 independent experiments, >200 cells per condition were analysed. **i**, Representative fluorescence micrographs of U2OS-DFCP1-KO cells stably expressing mNG-DFCP1, or ATPase mutants mNG-DFCP1T189A and mNG-DFCP1K193A treated with 250 µM LLOMe or equal volume of DMSO (No treatment) for 5, 15 and 30 min before fixation and immunostained with anti-CHMP4B (yellow) and anti-LAMP1 (magenta). Nuclei are stained with Hoechst (blue). Scale bar, 5 µm; scale bar inset, 5 µm. **j**, Quantification graph showing dynamics of LysoTracker recovery in WT cells (U2OS-mNG-DFCP1) and ATPase mutants mNG-DFCP1T189A and mNG-DFCP1K193A. Cells were pre-treated for 30 min with 75 nM LysoTracker Deep Red. Shaded error bands correspond to mean ± s.e.m. from *n* = 4 independent experiments, >200 cells per condition were analysed. **k**, FRAP of U2OS-mNG-DFCP1, U2OS-mNG-DFCP1T189A and U2OS-mNG-DFCP1K193A after 15 min of LLOMe treatment. The graph illustrates FRAP analysis from 20 regions in U2OS-mNG-DFCP1, U2OS-mNG-DFCP1T189A and U2OS-mNG-DFCP1K193A cells. Shaded error bands denote mean ± s.e.m. from *n* = 3 independent experiments. Source numerical data are available in source data. Source data

**Extended Data Fig. 4:**
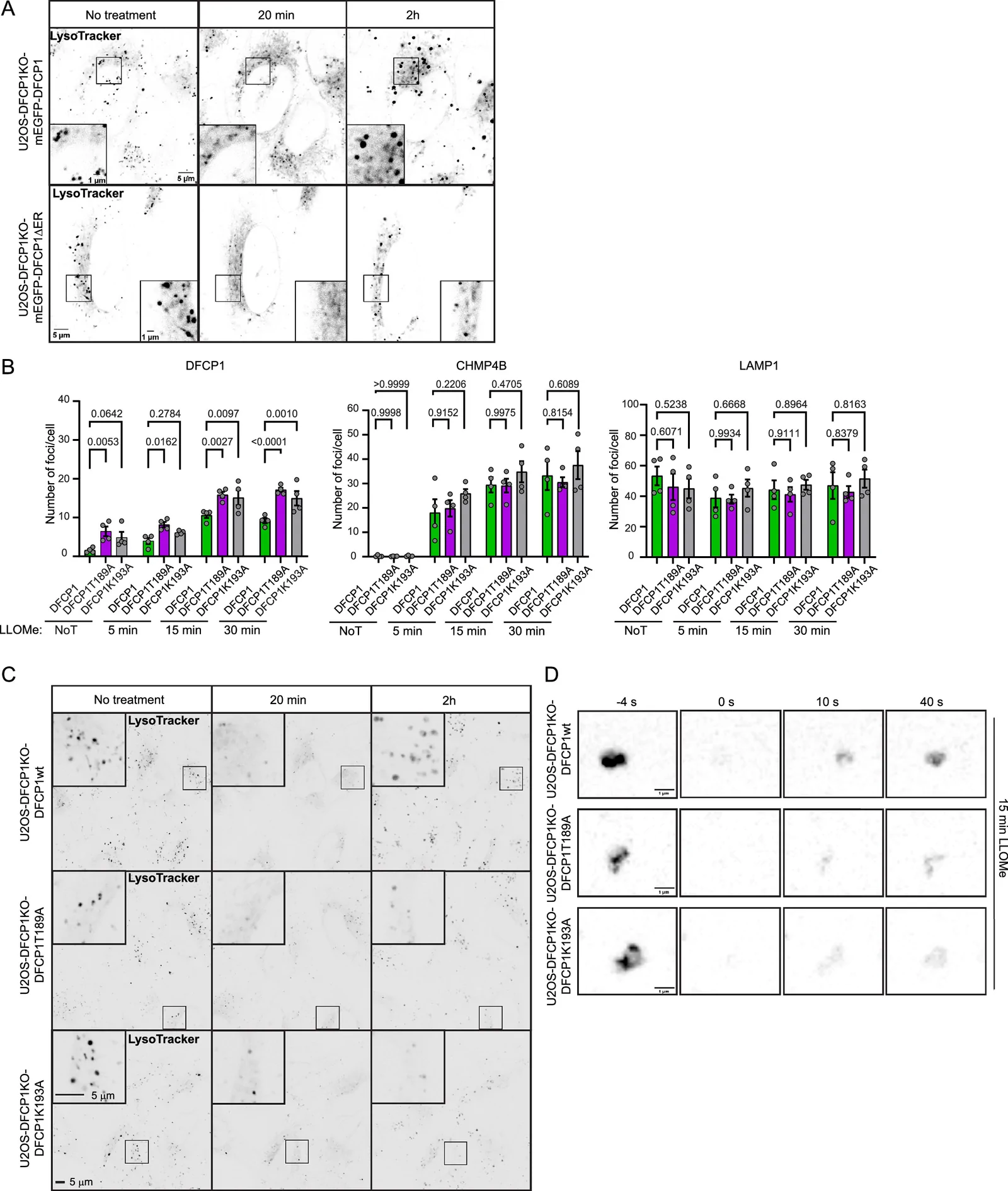
DFCP1 mediates lysosomal membrane repair in an ATPase dependent manner. **a)** Representative video stills from live-cell imaging experiments monitoring LysoTracker recovery after induced lysosomal damage using 250 µM LLOMe. WT (U2OS-DFCP1-KO-mEGFP-DFCP1) and U2OS-DFCP1-KO-mEGFP-DFCP1ΔER cells were pre-treated with 75 nM LysoTracker Deep Red for 30 min before the addition of LLOMe. Video stills correspond to Fig. 5c. Scale bar, 5 µm, scale bar inset: 1 µm. **b)** U2OS-DFCP1-KO cells stably expressing wild-type mNG-DFCP1, and ATPase mutants mNG-DFCP1T189A, and mNG-DFCP1K193A were treated with 250 µM LLOMe or DMSO (NoT) for 5, 15, and 30 min, fixed and immunostained for anti-CHMP4B and anti-LAMP1. Quantification graph shows number of DFCP1, CHMP4B, and LAMP1 foci per cell in wild-type and ATPase mutants upon LLOMe treatment. The graph corresponds to data shown in Fig. 5i. Error bars denote mean ± SEM from *n* = 4 independent experiments, >742 cells per condition per time point were analysed. Two-way ANOVA, Dunnett’s multiple-comparisons test. **c)** Representative video stills from live-cell imaging experiments monitoring LysoTracker recovery after induced lysosomal damage using 250 µM LLOMe. WT (U2OS-DFCP1KO-mNG-DFCP1) and ATPase mutants (U2OS-DFCP1KO-mNG-DFCP1T189A, U2OS-DFCP1KO-mNG-DFCP1K193A) were pre-treated with 75 nM LysoTracker Deep Red for 30 min before the addition of LLOMe. Video stills correspond to Fig. 5j. Scale bar, 5 µm, scale bar inset: 5 µm. **d)** Representative images showing WT U2OS-DFCP1KO-mNG-DFCP1, U2OS-DFCP1KO-mNG-DFCP1T189A, and U2OS-DFCP1KO-mNG-DFCP1K193A foci before (-4 s), bleaching (0 s), 10 and 40 s after photobleaching at 15 min post-LLOMe treatment. Scale bar, 1 µm. Figure corresponds to quantification graph shown in Fig. 5k. Source numerical data are available in source data. Source data

The rapid mobilization of DFCP1-containing ER microdomains close to lysosomes in response to their damage suggested the possibility that DFCP1 could be involved in membrane repair. To measure the importance of DFCP1 for lysosomal membrane repair, we used the LysoTracker recovery assay^5,6^. These analyses showed that LysoTracker recovery was impaired in DFCP1 knockout (KO) cells^30^ compared with wild-type (WT) cells (Fig. 5a,b and Supplementary Video 17). To investigate whether ER binding of DFCP1 is required for its function in lysosomal membrane repair, we stably expressed either WT DFCP1 or DFCP1ΔER in DFCP1-KO cells and assessed lysosomal repair following LLOMe-induced damage using the LysoTracker recovery assay. While cells expressing WT DFCP1 efficiently restored lysosomal integrity after LLOMe treatment, lysosomal repair was strongly impaired in cells expressing DFCP1ΔER (Fig. 5c, Extended Data Fig. 4a and Supplementary Video 18). These results demonstrate that ER binding of DFCP1 is essential for its role in mediating lysosomal membrane repair and support a functional requirement for ER association in the DFCP1-dependent repair process. As an additional assay for membrane repair we also stained WT and DFCP1-KO cells with antibodies against the galectin LGALS3/GAL3, which is known to be recruited to damaged lysosome membranes^6,40^. In agreement with the LysoTracker recovery data, we detected much stronger LGALS3 staining in lysosomes of DFCP1-KO cells than in WT cells after recovery periods of one or two hours after LLOMe-mediated lysosome damage (Fig. 5d,e).

Our previous work has shown that the NPC1 inhibitor U18666A, which causes accumulation of cholesterol in the lysosome membrane, makes the lysosomes partially refractory to lysosome damage^15^. We now found that U18666A treatment before LLOMe inhibited mobilization of DFCP1 around lysosomes (Fig. 5f,g). Collectively, these analyses demonstrate that DFCP1 is required for efficient lysosomal membrane repair.

To understand whether the ability of DFCP1 to bind and hydrolyse ATP is important for its function in lysosome repair, we took advantage of previously described mutants that affect its ATP-binding and ATPase activity^30^. Upon damage, these mutants showed increased persistence at damage sites compared with WT DFCP1 (Fig. 5h), without affecting CHMP4B recruitment or overall LAMP1 (Fig. 5i and Extended Data Fig. 4b). While DFCP1-KO cells reconstituted with WT DFCP1 showed normal lysosomal LysoTracker recovery after LLOMe treatment, cells reconstituted with the ATP-binding defective K193A mutant or the ATPase-defective T189A mutant failed to rescue LysoTracker recovery (Fig. 5j and Extended Data Fig. 4c). Fluorescence recovery after photobleaching (FRAP) analysis further showed that both ATPase mutants displayed reduced fluorescence recovery relative to WT DFCP1 15 min after LLOMe treatment, indicating reduced dynamics at damage sites (Fig. 5k and Extended Data Fig. 4d). This demonstrates that the ability of DFCP1 to bind and hydrolyse ATP is central to its function in lysosomal membrane repair.

### DFCP1 promotes focal accumulation of VPS13C at damaged lysosomes

**Fig. 6:**
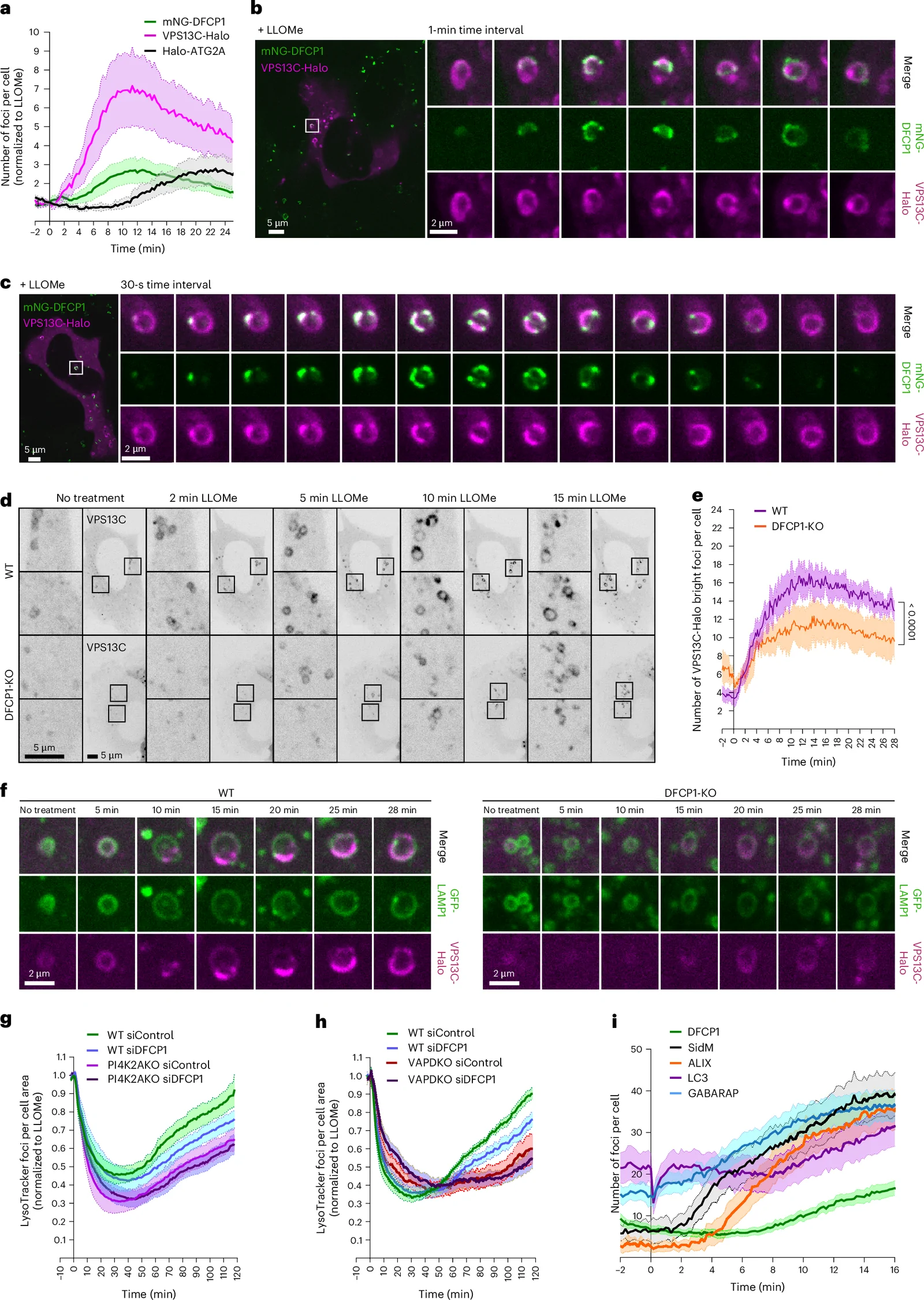
ER-associated lipid transfer machinery cooperates with DFCP1 during lysosomal membrane repair. **a**, The quantification graph of live-cell imaging experiments show the normalized accumulation kinetics of VPS13C-Halo, mNG-DFCP1, and Halo-ATG2A after treatment with 250 µM LLOMe. VPS13C was the first factor recruited to damaged lysosomes, accumulating approximately 2 min after LLOMe addition, followed by DFCP1 at ~6 min and ATG2A at ~9 min. DFCP1 dynamics were measured in U2OS-DFCP1-KO cells stably expressing mNG-DFCP1, VPS13C dynamics in WT U2OS cells transiently expressing VPS13C-Halo, and ATG2A dynamics in U2OS cells expressing endogenous Halo-ATG2A. Images were acquired every 20 s for 30 min. Shaded error bands denote mean ± 95% CI from 33 cells, from 23 live-cell imaging videos from *n* = 3 independent experiments for VPS13C-Halo; from 22 live-cell imaging videos from *n* = 5 independent experiments, >100 cells for mNG-DFCP1; from 20 live-cell imaging videos from *n* = 7 independent experiments, >200 cells for Halo-ATG2A. **b**,**c**, Representative video stills from live-cell imaging experiments of U2OS-DFCP1-KO cells stably expressing mNG-DFCP1 and transiently expressing VPS13C-Halo following treatment with 250 µM LLOMe. Images were acquired every 10 s for 30 min. The 1 min (**b**) or 30 s (**c**) time intervals are shown. Insets highlight the spatial organization of VPS13C- and DFCP1-positive structures during the repair response. Scale bar, 5 µm; scale bar insets, 2 µm. **d**, Representative fluorescence micrographs of WT U2OS and U2OS-DFCP1-KO cells transiently expressing VPS13C-Halo following treatment with 250 µM LLOMe. In WT cells, VPS13C accumulated in discrete punctate microdomains. VPS13C recruitment seemed more diffuse in DFCP1-deficient cells. Images were acquired every 10 s for 30 min. Scale bar, 5 µm; scale bar insets, 2 µm. **e**, The quantification graph of VPS13C-Halo microdomain formation shown in **d**. LLOMe was added at time 0. Shaded error bands denote mean ± s.e.m. from *n* = 6 independent experiments for WT U2OS cells, *n* = 5 independent experiments for U2OS-DFCP1-KO cells, >25 cells per condition were analysed. Two-way ANOVA. **f**, Representative video stills from tracked lysosomes from live-cell imaging experiments of WT U2OS and U2OS-DFCP1-KO cells transiently expressing GFP-LAMP1 (green) and VPS13C-Halo (magenta) following treatment with 250 µM LLOMe. VPS13C recruitment to LAMP1-positive lysosomes seemed diffuse in the absence of DFCP1. Images were acquired every 10 s for 30 min. Scale bar, 2 µm. **g**, Quantification of lysosomal repair measured by LysoTracker recovery in WT HeLa and HeLa-PI4K2AKO cells transfected with either siControl or siDFCP1. Lysosomal repair was assessed by quantifying the number of LysoTracker-positive foci normalized to cell area over time following LLOMe-induced damage. Images were acquired every 1 min for 120 min. Shaded error bands denote mean ± s.e.m. from *n* = 5 independent experiments, >250 cells per condition. **h**, The quantification graph of lysosomal repair measured by LysoTracker recovery in WT HeLa and HeLa-VAP-DKO cells transfected with either siControl or siDFCP1. Lysosomal repair was assessed by quantifying the number of LysoTracker-positive foci normalized to cell area. Images were acquired every 1 min for 120 min. Shaded error bands denote mean ± s.e.m. from *n* = 6 independent experiments for HeLa siControl and VAP-DKO siDFCP1, and from *n* = 5 independent experiments for HeLa siDFCP1 and VAP-DKO siControl, >150 cells per condition. **i**, The quantification graph from live-cell imaging experiments of the recruitment dynamics of factors involved in lysosomal membrane repair following LLOMe-induced damage. U2OS-DFCP1-KO cells stably expressing mNG–DFCP1 or mCherry–DFCP1 were transiently co-transfected with the PI4P probe mCherry–SidM, GFP–ALIX, mCherry–LC3 or mCherry–GABARAP and imaged every 10 s for 30 min following addition of 250 µM LLOMe at time 0. Only cells expressing low levels of the respective markers were included in the analysis. Quantification shows the number of fluorescent foci per cell over time. GABARAP displayed the earliest recruitment to damaged lysosomes, followed by the PI4P probe SidM, ALIX, DFCP1 and finally LC3. Shaded error bands denote mean ± 95% CI from individual cells, for DFCP1 74, SidM 20, LC3 27, GABARAP 27 and ALIX 23, from at least *n* = 3 independent experiments per marker. Source numerical data are available in source data. Source data

**Extended Data Fig. 5:**
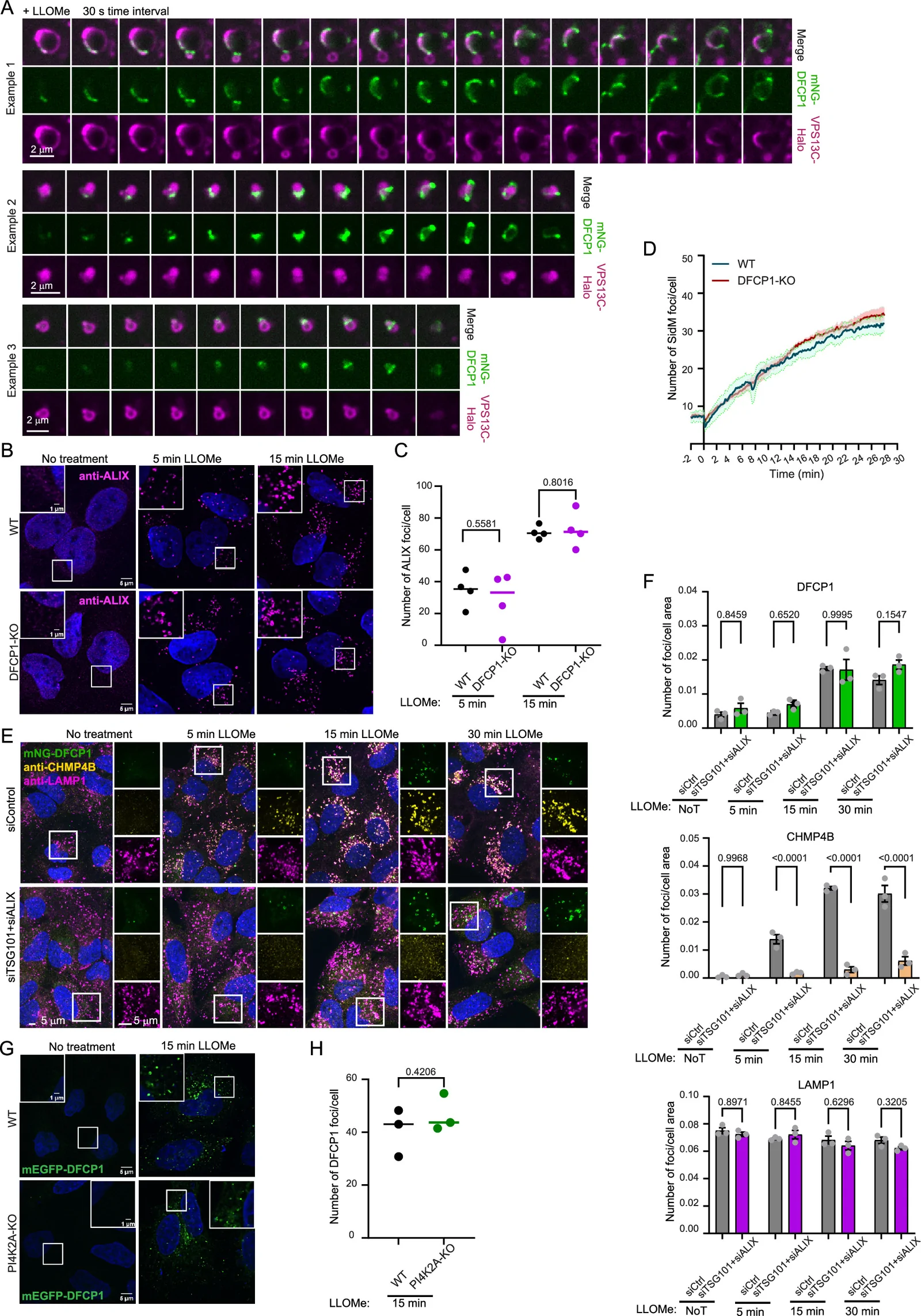
DFCP1 response to lysosome damage in ESCRT- and PI4P-independet. **a)** Additional examples (corresponding to Fig. 6b, c) of co-localization between VPS13C-Halo and mNG-DFCP1 during lysosomal damage. Images were taken every 10 s for 30 min. Scale bar, 2 µm. **b)** Representative fluorescence micrographs of U2OS (WT) and U2OS-DFCP1-KO cells treated with 250 µM LLOMe for 5 or 15 min and fixed. Cells were immunostained with anti-ALIX (magenta). Nuclei are stained with Hoechst (blue). Scale bar, 5 µm, scale bar inset: 1 µm. **c)** Quantification graph of data shown in **b**). There was no significant difference in the number of ALIX foci per cells between WT and DFCP1-KO cells at 5 or 15 min post-LLOMe treatment. Every dot represents mean foci per cell per experiment. Graph represents mean from *n* = 4 independent experiments, >90 cells per experiment per condition, unpaired two-sided *t*-test. **d)** The quantification graph from live-cell imaging experiments of U2OS (WT) and DFCP1-KO cells transiently transfected with PI4P probe SidM-mCherry. WT and DFCP1-KO cells have similar dynamics of recruitment and number of SidM foci per cell. LLOMe was added at 0 min. Error bars denote mean ± SEM from *n* = 5 independent experiments., >53 cells per condition. **e)** U2OS-mNG-DFCP1 cells were co-transfected with siRNAs targeting ESCRT proteins TSG101 and ALIX (siTSG101siALIX) or with non-targeting siRNA control (siCtrl). 48 h post-transfection, cells were treated with 250 μM LLOMe for 15 min to induce lysosomal damage or with DMSO (No treatment) and fixed. Cells were immunostained with anti-CHMP4B (yellow) and anti-LAMP1 (magenta). Scale bars: 5 µm, scale bar insets: 5 µm. **f)** Quantification graph of data shown in **e**). mNG-DFCP1 recruitment to damaged lysosomes was not affected in siTSG101siALIX when compared to the siCtrl. As expected, there is a significant drop in number of CHMP4B foci per cell upon lysosomal damage in cells depleted of TSG101 + ALIX. Error bars denote mean ± SEM from *n* = 3 independent experiments., >300 cells per condition per time point. Two-way ANOVA, Šidák’s multiple-comparisons test. **g)** Representative fluorescence images of HeLa (WT) and HeLa-PI4K2A-KO cells transiently expressing mEGFP-DFCP1. After treatment with LLOMe for 15 min or DMSO (No treatment) cells were fixed and stained with Hoechst (blue). Scale bar, 5 µm, scale bar inset: 1 µm. **h)** Quantification graph of data shown in **g**). Every dot represents mean foci per cell per experiment. Graph represents mean from *n* = 3 independent experiments, >75 cells per experiment, unpaired two-sided *t*-test. Source numerical data are available in source data. Source data

Lipid channel proteins mediate high-capacity lipid transfer between membranes^41^, and the lipid channels ATG2 and VPS13C have been found to be required for lysosomal membrane repair^14,16^. To monitor whether DFCP1 regulates recruitment or organization of these proteins, we used cells expressing Halo-ATG2 (ref. ^42^) or VPS13C-Halo^16^ and monitored their response to LLOMe by live spinning-disk fluorescence microscopy. Consistent with previous reports, both Halo-ATG2A and VPS13C-Halo were recruited in response to LLOMe-induced lysosomal damage. Strikingly, VPS13C-Halo recruitment and DFCP1 mobilization peaked at the same time whereas Halo-ATG2A was recruited several minutes later (Fig. 6a). The strong correlation between VPS13C recruitment and DFCP1 mobilization prompted us to monitor the colocalization of these proteins after lysosome damage. Live-cell microscopy showed that VPS13C-Halo accumulated uniformly in response to LLOMe, and this was followed by proximal recruitment of DFCP1 as sharply defined puncta that extended to form ring- or cup-shaped microdomains. VPS13C then accumulated strongly into these microdomains and remained there for a few minutes until both DFCP1 and VPS13C became more diffuse (Fig. 6b,c, Extended Data Fig. 5a and Supplementary Video 19). To address whether VPS13C accumulation into microdomains depends on DFCP1, we studied VPS13C-Halo localization in LLOMe-treated WT and DFCP1-KO U2OS cells. Notably, in cells lacking DFCP1, VPS13C-Halo failed to cluster into microdomains in response to LLOMe and instead was recruited diffusely around lysosomes (Fig. 6d–f and Supplementary Video 20). This indicates that DFCP1 mediates focal accumulation of VPS13C lipid channels at ER microdomains proximal to damaged lysosomes.

### Relationship of DFCP1 to other components of the lysosomal damage response

The DFCP1 redistribution after lysosome damage raised the question whether it is linked to ESCRT- or PI4P-mediated lysosome repair^5,6,14,15^. To monitor whether ESCRT recruitment on lysosomes depends on DFCP1, we monitored whether the ESCRT scaffold ALIX is recruited to LLOMe-treated lysosomes in the absence of DFCP1. We detected no effect on ALIX recruitment in DFCP1-KO cells^30^ when compared with WT cells, indicating that ESCRT recruitment is independent of DFCP1 (Extended Data Fig. 5b,c). Likewise, we observed no effect on PI4P formation on damaged lysosomes, monitored with SidM as a probe for PI4P^43^ (Extended Data Fig. 5d). To investigate whether the DFCP1 response to lysosome damage requires ESCRT proteins or PI4P, we monitored DFCP1 dynamics in cells treated with siRNAs against the ESCRT proteins TSG101 and ALIX^5,6^, or in the knockout of PI4K2A, the phosphatidylinositol 4-kinase responsible for PI4P formation on damaged lysosomes^14,15^. Neither the knockdown of ESCRT nor the knockout of PI4K2A resulted in any effect on DFCP1 mobilization after LLOMe treatment (Extended Data Fig. 5e–h). These results indicate that the response of DFCP1 to lysosomal damage is independent of ESCRTs and PI4P.

**Extended Data Fig. 6:**
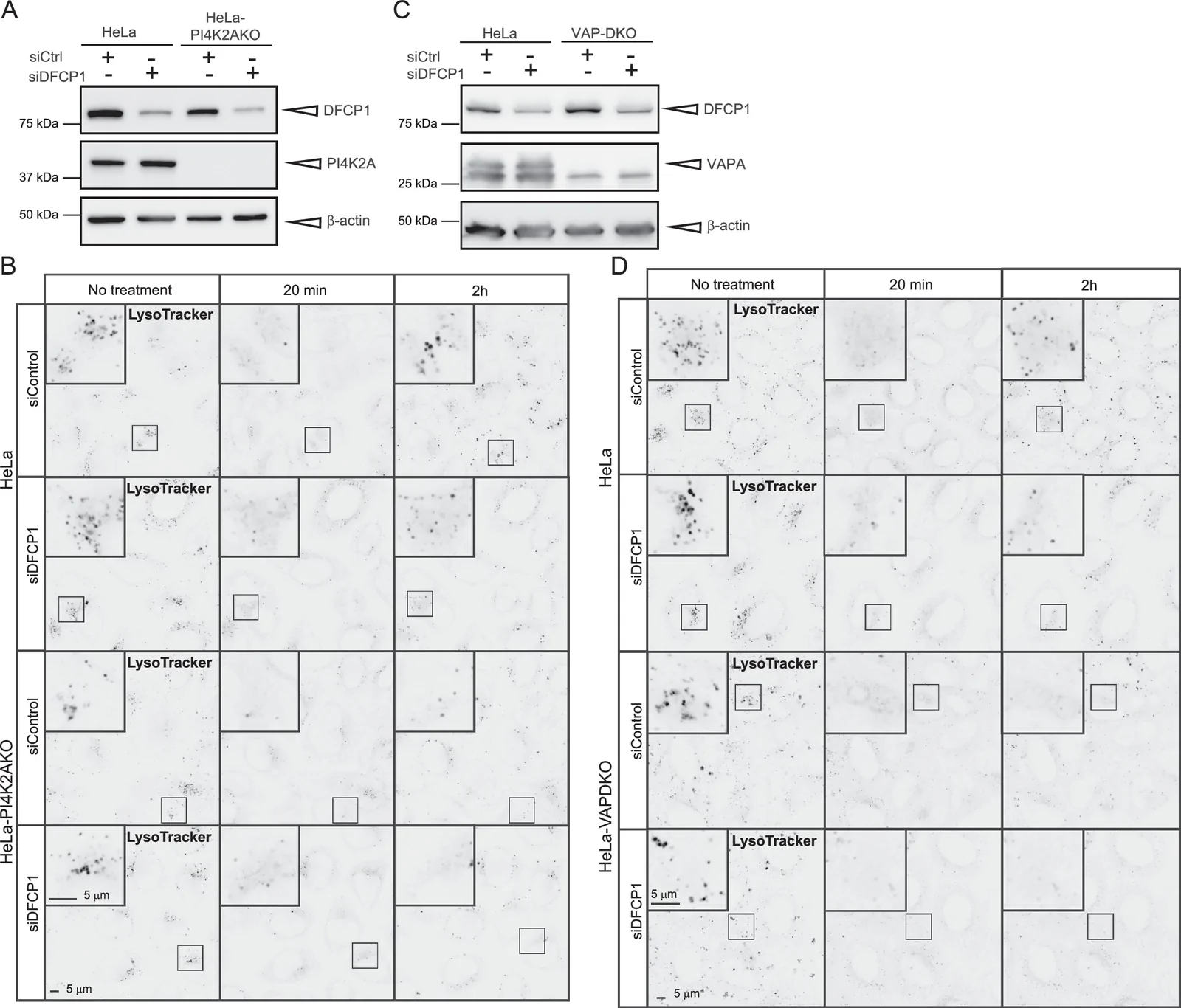
ER-associated lipid transfer machinery cooperates with DFCP1 during lysosomal membrane repair. **a)** Representative western blot showing knockdown efficiency of DFCP1 in HeLa and HeLa-PI4K2AKO cells corresponding to the experiments shown in Fig. 6g. β-actin used as a loading control. **b)** Representative video stills from lysosomal repair assays in HeLa and HeLa-PI4K2AKO cells transfected with either siRNAs targeting DFCP1 (siDFCP1) or a non-targeting control siRNA (siControl) and subjected to live-cell imaging following LLOMe-induced lysosomal damage. Images correspond to the quantification shown in Fig. 6g and were acquired every 1 min for 120 min. Scale bar, 5 µm, scale bar inset: 5 µm. **c)** Representative western blot showing knockdown efficiency of DFCP1 in HeLa and VAP-DKO cells used for the experiments shown in Fig. 6h. β-actin used as a loading control. **d)** Representative video stills from live-cell imaging repair experiments of HeLa and VAP-DKO cells depleted of DFCP1 (siDFCP1) or a non-targeting control siRNA (siControl). Images were taken every 1 min for 120 min and correspond to data shown in Fig. 6h. Scale bar, 5 µm, scale bar inset: 5 µm. Unprocessed blots are available in source data. Source data

To investigate the hypothesis that DFCP1 is part of the ER-mediated lipid transport response to lysosome damage, we performed epistasis-type experiments, which involved siRNA-mediated knockdown of DFCP1 in HeLa cells with KO of VAP-A/B or PI4K2A. Using the LysoTracker recovery assay we found that lysosome repair was inhibited in PI4K2A cells as shown previously^14,15^ and in VAP-A/B KO cells. However, there was no additive effect of knocking down DFCP1 in cells lacking PI4K2A or VAP-A/B (Fig. 6g,h and Extended Data Fig. 6a–d). This supports the idea that DFCP1 operates in the same pathway as PI4K2A and VAP-A/B (ER-mediated lipid transport).

To understand the sequence of events in the lysosomal damage response, we used spinning-disk microscopy of U2OS cells expressing fluorescently tagged components of the repair machinery. With frame rates as frequent as 10 s, the numbers of fluorescent puncta after LLOMe addition were measured and quantified (Fig. 6i). Of note, the ATG8 family protein GABARAP showed the fastest response, with a detectable increase already 1 min after LLOMe addition. The next fastest response was observed with the PI4P probe SidM, which increased 2 min after LLOMe, and this was followed by the ESCRT protein ALIX after 4 min and DFCP1 after 6 min. Notably, the ATG8 family protein LC3B showed the slowest response with a detectable increase 8 min after LLOMe addition. These results are consistent with the idea that CASM with GABARAP triggers ESCRT recruitment and that PI4P-dependent ER–lysosome MCSs precede DFCP1 recruitment^10^.

### DFCP1 depletion causes increased lysophagy

**Fig. 7:**
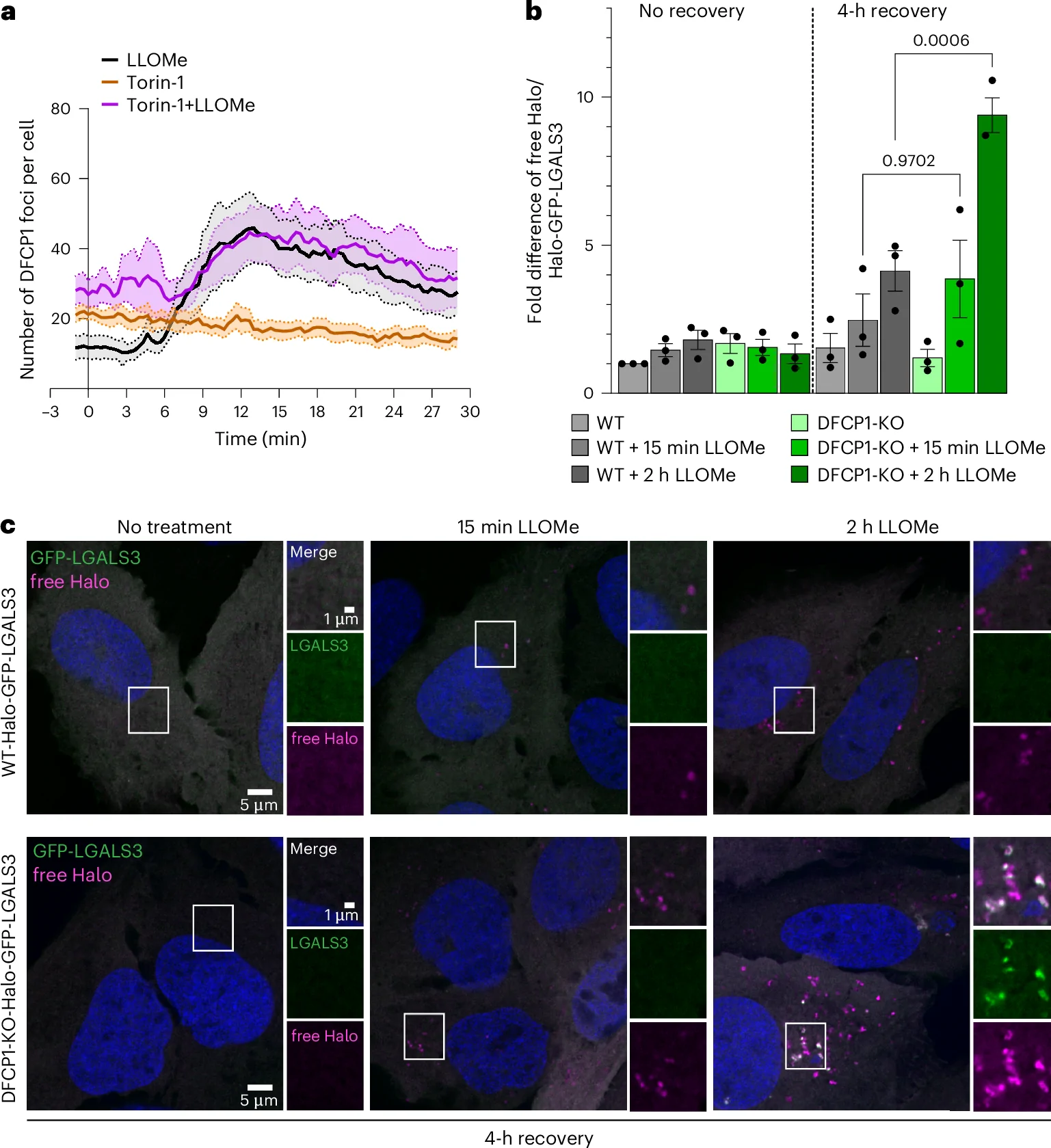
Absence of DFCP1 increases lysophagy following lysosome damage. **a**, The quantification graph of U2OS-mNG-DFCP1 cells following treatment with 100 nM Torin1 for 2 h, 250 µM LLOMe (added at 0 min), or Torin1 + LLOMe. Torin1 treatment increased the basal number of DFCP1 foci before induction of lysosomal damage (before 0 min) compared with cells treated with LLOMe alone, consistent with autophagy induction. Following addition of LLOMe, Torin1-treated cells displayed a further increase in DFCP1 foci. Shaded error bands denote mean ± s.d. from more than eight videos, >30 cells per condition per experiment were analysed from *n* = 2 independent experiments. **b**, Quantification graph of in-gel fluorescence analyses measuring induction of lysophagy using Lysoflux assay. Following washout of the free ligand, which prevents labelling of newly synthesized protein, only the pre-labelled pool of Halo-LGALS3 is tracked. Upon autophagic delivery to lysosomes, LGALS3 is degraded while the protease-resistant HaloTag is released, appearing as a free Halo band. After a 4 h LLOMe recovery, no significant difference was observed between WT (U2OS-Halo-GFP-LGALS3) and U2OS-DFCP1-KO-Halo-GFP-LGALS3 cell lines following 15 min of LLOMe treatment. In contrast, DFCP1-KO-Halo-GFP-LGALS3 cells that were treated for 2 h with 250 µM LLOMe followed by a 4-h recovery showed a significant increase in the fold change of free HaloTag when compared with WT. Error bars denote mean ± s.d. from *n* = 3 independent experiments. One-way ANOVA with Tukey’s multiple-comparisons test. **c**, Representative fluorescence micrographs of WT-Halo-GFP-LGALS3 and DFCP1-KO-Halo-GFP-LGALS3 cells used for immunoblotting analyses shown in **b**. Cells were treated for 15 min or 2 h with LLOMe, washed off and left to recover over 4 h. Before treatments cells were incubated for 20 min with TMR, a fluorescent HaloTag ligand, and washed three times with medium. After treatment with LLOMe or DMSO (no treatment), cells were fixed and imaged for GFP-LGALS3 (green), free Halo (magenta), and Hoechst (blue). Scale bar, 5 µm; scale bar inset, 1 µm. Source numerical data are available in source data. Source data

Previous work from our laboratory has shown that KO of DFCP1 reduces the ability of cells to perform several types of selective autophagy, including mitophagy and aggrephagy^30^. We therefore asked whether DFCP1 would also be involved in lysophagy. To probe the relationship between DFCP1 puncta during autophagy induction and lysosome damage, we treated cells with the mTORC1 inhibitor Torin1, which is known to induce bulk autophagy^44^, and measured the number of DFCP1 puncta. As expected, Torin1 increased the number of DFCP1 puncta at the start of imaging (time point 0). Upon subsequent addition of LLOMe, DFCP1 puncta continued to rise over time (Fig. 7a and Supplementary Video 21). The magnitude of the DFCP1 increase after LLOMe in Torin1-treated cells was similar to the response observed with LLOMe alone. This indicates that despite the elevated baseline of DFCP1 puncta under Torin1 treatment, cells still mount an additional DFCP1 response to lysosomal damage, suggesting the existence of a DFCP1 pool that operates independently of its canonical role.

**Extended Data Fig. 7:**
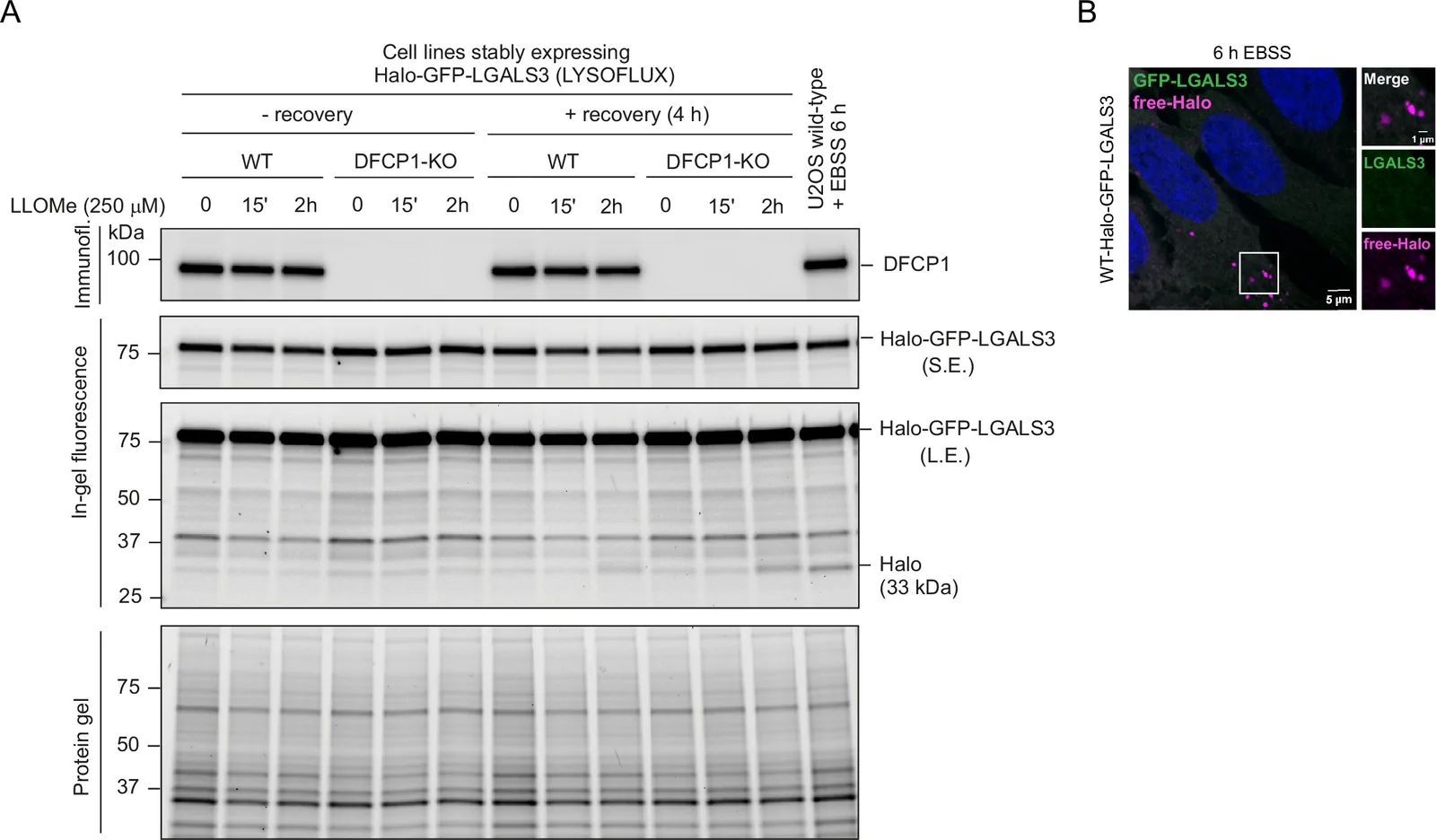
DFCP1 depletion increases lysophagy. **a)** In-gel fluorescence assay and immunoblot corresponding to the quantification shown in Fig. 7b. WT U2OS cells were starved in EBSS for 6 h to induce autophagy and allow detection of free HaloTag, used here as a control. Non-specific bands were detected between 37–50 kDa, in line with what has been previously published^45^. S.E., short exposure; L.E., long exposure. **b)** Representative image of WT cells after 6 h of EBSS treatment from *n* = 3 independent experiments showing the presence of free HaloTag (magenta) but no detectable LGALS3 signal (green). Scale bar, 5 µm, scale bar inset: 1 µm. Unprocessed blots are available in source data. Source data

To directly measure the importance of DFCP1 in lysophagy, we used Halo-tagged LGALS3 as a lysophagy marker. In this assay, cells are pulse-labelled with a cell-permeable HaloTag ligand, which binds covalently and irreversibly to the HaloTag moiety. This covalent binding renders the HaloTag itself resistant to lysosomal proteases, while the fused cargo protein (LGALS3) remains susceptible to lysosomal degradation^45^. The ratio of free HaloTag to full-length protein serves as a quantitative readout of lysophagic flux. As expected LLOMe treatment caused a clear increase in free HaloTag signal detected by western blotting and fluorescence microscopy in WT cells, confirming induction of lysophagy, and this was most pronounced after 2 h of LLOMe incubation followed by a 4-h chase period to allow lysosomal processing (Fig. 7b,c and Extended Data Fig. 7a,b). Of note, there was a strong increase in free Halo tag in LLOMe-treated DFCP1-KO cells both at 15 and 120 min of LLOMe treatment. This indicates that KO of DFCP1, in spite of inhibiting other types of selective autophagy, promotes lysophagy.

### Loss of DFCP1 exacerbates vacuole damage induced by *Listeriamonocytogenes*

**Fig. 8:**
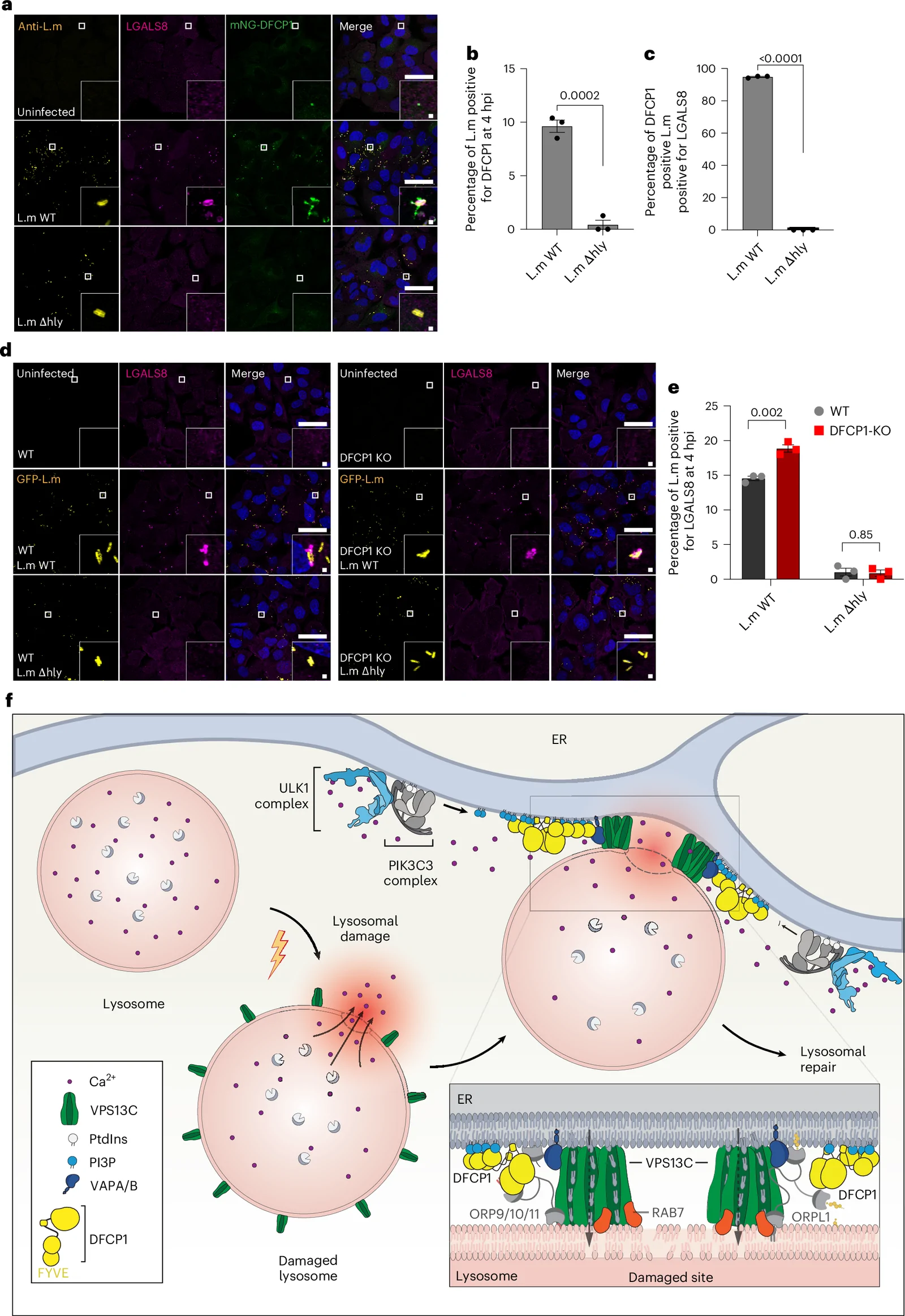
DFCP1 accumulates at *Listeria*-induced damage sites, and its loss leads to elevated damage. **a**, Immunofluorescence staining of LGALS8 and *L.* *monocytogenes* in U2OS-DFCP1-KO cells stably expressing mNG-DFCP1, with or without infection by WT or *Δhly* strains at 4 h post-infection (hpi). Scale bar, 50 µm; scale bar inset, 1 µm. **b**, Quantification of the percentage of WT and *Δhly L.* *monocytogenes* positive for mNG-DFCP1 at 4 hpi (related to **a**). Error bars denote mean ± s.e.m. from *n* = 3 biological replicates, a total of 619 WT and 379 *Δhly* bacteria were analysed. Unpaired two-sided *t*-test. **c**, Quantification of the percentage of DFCP1-positive *L.* *monocytogenes* that were also positive for LGALS8 (related to **a**). Error bars denote mean ± s.e.m. from *n* = 3 biological replicates, a total of 619 WT and 379 *Δhly* bacteria were analysed. Unpaired two-sided *t*-test. **d**, LGALS8 staining in WT and DFCP1-KO U2OS cells with or without WT or *Δhly L.* *monocytogenes* infection at 4 hpi. Scale bar, 50 µm; scale bar inset, 1 µm. **e**, Quantification of WT and *Δhly L.* *monocytogenes* positive for LGALS8 in WT versus DFCP1-KO cells at 4 hpi (related to **d**). Error bars denote mean ± s.e.m. from *n* = 3 biological replicates, a total of 470 and 477 WT bacteria, and 297 and 256 *Δhly* bacteria, were analysed in WT and DFCP1-KO cells, respectively. Unpaired two-sided *t*-test. **f**, Suggested model for DFCP1-mediated lysosomal membrane repair. Following lysosomal membrane damage, lysosomal Ca^2+^ release triggers activation of the repair response, leading to PIK3C3-dependent generation of PI3P on proximal ER domains in a VAP-dependent manner. DFCP1 is recruited to these sites via its tandem FYVE domains and assembles into dynamic omegasome-like structures at ER–lysosome contact sites. DFCP1 promotes lysosomal membrane repair by concentrating lipid transfer proteins, including VPS13C, at damaged lysosomes thereby enabling efficient lipid delivery to damaged lysosomal membranes and restoration of lysosomal integrity. Source numerical data are available in source data. Source data

Several internalized pathogens escape phagolysosomal degradation by entering into cytosol from bacterial vacuoles^46^. One of these is the gram-positive bacterium *Listeria* *monocytogenes*, which produces a toxin that causes acute damage of the bacterial vacuole membrane^47^. We investigated if DFCP1 protects against *L.* *monocytogenes*-induced vacuolar damage. To monitor if DFCP1 is recruited to *L.* *monocytogenes* vacuoles, mNG-DFCP1-expressing U2OS cells were infected with *L.* *monocytogenes*, and eventual colocalization between the internalized bacteria and mNG-DFCP1 was studied by confocal fluorescence microscopy. The microscopy showed a strong accumulation of mNG-DFCP1 to the bacteria-containing vacuoles, and this did not occur with a *L.* *monocytogenes* mutant unable to damage vacuole membranes, Δhly (Fig. 8a–c). This demonstrates that DFCP1-containing ER microdomains respond to *L.* *monocytogenes* infection.

To investigate whether DFCP1 is involved in the cellular response to *L.* *monocytogenes* infection, we monitored lysosome damage after 4 h of infection. For this purpose we used an antibody against the galectin LGALS8/GAL8, which is known to mark damaged bacterial vacuoles, including those of *L.* *monocytogenes*^48^, to stain WT and DFCP1-KO U2OS cells. As expected, there was a strong increase in LGALS8-positive profiles in WT cells at 4 h of infection compared with uninfected cells (Fig. 8d). Of note, however, there was a further increase in *L.* *monocytogenes* spots positive for LGALS8 in the DFCP1-KO cells compared with the WT cells (Fig. 8e). This indicates that DFCP1 is involved in the cellular response to *L.* *monocytogenes* infection.

## Discussion

While previous work has shown that PI4K2A and its product PI4P promote lysosomal membrane repair via recruitment of lipid transfer proteins^14,15^, this study has revealed that PIK3C3 and PI3P also play an important role. PI3P formed on ER domains functions to recruit DFCP1, which promotes lysosome repair in response to both chemical damage and microbial insults. DFCP1 accumulates on microdomains of the ER, in response to lysosome damage, whose dynamics are regulated by DFCP1’s ability to bind and hydrolyse ATP. The lipid channel VPS13C, which is initially recruited uniformly at damaged lysosomes, accumulates into these microdomains, and we propose that DFCP1 mediates lysosome repair by concentrating the lipid transport machinery at ER–lysosome contact sites where high lipid transport is required (Fig. 8f).

Sensing of lysosomal membrane damage seems to be tightly linked to leakage of Ca^2+^ from the lysosome lumen to the cytosol, as reported for activation of repair mechanisms involving ESCRT proteins^5^, ER-mediated lipid transfer^14^, sphingomyelinase activation^7^ and annexin-mediated repair^8^. We discovered that DFCP1 response to lysosome damage is highly Ca^2+^ dependent and is much more sensitive than ESCRT recruitment to Ca^2+^ chelation. The sensitivity of DFCP1 mobilization to inhibition of the lysosomal Ca^2+^ channel TRPML1 indicates that local Ca^2+^ flux from the damaged lysosome, promoted by TRPML1 activation, is a major trigger of the DFCP1 response. This could explain the local recruitment of DFCP1 at ER microdomains proximal to damaged lysosomes. We found that elevated Ca^2+^ levels after lysosome damage activate PIK3C3 via the protein kinase ULK1, similar to what has been reported for autophagy induction where Ca^2+^ flux from the ER triggers PIK3C3 via ULK1 (refs. ^27–29^).

DFCP1-containing ER domains have previously been shown to be associated with phagophores and lipid droplets in a PI3P-dependent manner^18,30,49–51^. In autophagy, DFCP1-driven omegasomes have been proposed to provide lipids to the growing phagophore^30,52^. In lipid droplet homeostasis, DFCP1-positive ER membranes provide platforms for fusion of small lipid droplets into larger ones to promote lipid droplet expansion^53^, and DFCP1 also contributes to lipid droplet growth by inhibiting the activity of a lipid droplet-metabolizing triglyceride lipase^54^. With the additional function for DFCP1 in lysosome repair, a pattern emerges that DFCP1 controls focal ER domains that can associate with other cellular structures to control their biogenesis, size or integrity. Characterizing the structure of membrane-bound DFCP1 and the function of its ATPase domain will be essential for understanding the general function for DFCP1 in ER-associated processes.

In addition to PI3P and Ca^2+^, VAP family proteins are also required for the DFCP1 response to lysosome damage. We found that the DFCP1 response was sensitive to double knockout of VAP-A and VAP-B but also to knockdown of a third VAP family member, MOSPD2. The involvement of VAP family proteins is interesting because these proteins have already been shown to mediate MCS formation between the ER and damaged lysosomes for lipid transfer^15^. They have also been found to interact with ULK1 and FIP200 (ref. ^55^), raising the possibility that, after lysosome damage and subsequent formation of lysosome–ER contacts, VAP-mediated recruitment of the ULK1 complex might create a local environment favourable for PIK3C3 activation and thereby for DFCP1 recruitment. In future studies it will be pertinent to investigate the relationships between MCS formation and DFCP1 function in membrane repair.

The involvement of DFCP1 in selective autophagy of mitochondria and protein aggregates^30^ made us initially hypothesize that its depletion would inhibit lysophagy. Instead, we detected a profound increase in lysophagy in DFCP1-KO cells in response to LLOMe. This can probably be explained as a response to faulty lysosomal membrane repair in the absence of DFCP1. Consistent with this notion, DFCP1, although facilitating mitophagy and aggrephagy, is not essential for neither bulk nor selective autophagy^18,30^, suggesting that additional ER proteins have overlapping functions in ER remodelling during autophagy.

Our detailed live microscopy revealed notable temporal differences between the various components of the lysosomal damage response, with the CASM component GABARAP responding first, followed by PI4P, ESCRT and DFCP1. We also found that VAPB responds earlier to lysosome damage than DFCP1, which is consistent with the finding that VPS13C, which is a binding partner of VAP proteins^56^, is also recruited before DFCP1. Two proteins that responded later than DFCP1 were LC3B and ATG2. This is noteworthy as both these proteins are important for lysophagy^40,57^. Further studies will be needed to clarify whether differential CASM and lipid transport machineries mediate lysosome repair and lysophagy.

Lysosomal membrane damage plays important roles in transmission of neurotoxic protein aggregates^58,59^ and in the cytosolic entry of intracellular pathogens^9,48,60^. In agreement with this we found that DFCP1-containing ER microdomains are recruited to the vacuoles of pathogenic *L.* *monocytogenes* and that KO of DFCP1 reduces bacterial escape from vacuoles. It will now be interesting to investigate the importance of DFCP1-mediated lysosomal membrane repair in neurodegenerative diseases and infections.

## Methods

### Reagents, cell culture and stable cell lines

The lysosome-damaging agents used in this study were L-leucyl-L-leucine methyl ester (cat. no. 16008) used at 250 μM, Gly-Phe-β-naphthylamide (cat. no. 14634) used at 250 μM and Ebastine (cat. no. 15372) used at 15 μM (all purchased from Cayman Chemical). Chlorpromazine hydrochloride (cat. no. C8138, Merck Life Science (Sigma)) was used at 20 μM. E64D (cat. no. A13259-5, Adooq Biosciences) was used at 50 μM. All agents were dissolved in dimethylsulfoxide (DMSO) and stored at −20 °C. Other reagents used in this study were LysoTracker Deep Red (cat. no. 12492, Molecular Probes) for live-cell imaging, used at 75 nM; ML-SI3 (cat. no. HY-139426, MedChemExpress) used at 50 μM; U18666A (cat. no. U3633-5MG, Merck Life Science (Sigma)) used at 2 μM; SAR405 (cat. no. S7682, Selleckchem) used at 1 μM or 5 μM; MRT68921 (cat. no. SML1644-5MG, Merck Life Science (Sigma)) used at 1 μM; BAPTA-AM (cat. no. A1076, Merck Life Science (Sigma)) used at 15 μM; Thapsigargin (cat. no. T7458, Thermo Fisher Scientific (Invitrogen)) used at 1.5 μM; and Torin1 (cat. no. 4247, Bio-Techne R&D systems Europe) used at 100 nM.

Human HeLa ‘Kyoto’ cells and U2OS cells (ATCC: HTB-96) were maintained in DMEM (Gibco), supplemented with 10% fetal bovine serum (FBS), 5 U ml^−1^ penicillin and 50 μg ml^−1^ streptomycin. Cells were maintained at 37 °C supplemented with 5% CO_2_. U2OS-mNG-DFCP1, U2OS-DFCP1-KO, U2OS-mNG-DFCP1T198A and U2OS-mNG-DFCP1K193A cells were previously published^30^. The HeLa-VAP-DKO stable cell line^32^ was obtained from Pietro De Camilli (Yale School of Medicine). The U2OS-Halo-ATG2A stable knock-in cell line was obtained from J. C. Schmidt (Michigan State University).

Third-generation lentiviruses were generated using procedures and plasmids as previously described^61^. In brief, tagged fusions of transgenes were generated as Gateway-compatible pENTR constructs using standard molecular biology techniques. From these vectors, lentiviral transfer vectors were generated by Gateway LR recombination into the lentiviral destination vector pLenti-PGK-Blast-DEST (Addgene plasmid #19065, a gift from E. Campeau and P. Kaufman). VSV-G pseudotyped lentiviral particles were packaged using a third-generation packaging system (Addgene plasmids #12251, #12253 and #12259, gifts from D. Trono). Cells were then transduced with low-virus titre and stable expressing populations were generated, either by antibiotic resistance or by flow cytometry. The weak PGK promoter was used for transgene expression to achieve low expression levels.

### siRNA transfections

Silencer Select siRNAs against TSG101 (#1, 5′-CCGUUUAGAUCAAGAAGUA-3′ and #2, 5′-CCUCCAGUCUUCUCUCGUC-3′), ALIX (#1, 5′-GCAGUGAGGUUGUAAAUGU-3′ and #2, 5′-CCUGGAUAAUGAUGAAGGA-3′), MOSPD2 (predesigned, cat. no. 4392420, #1 s46105 and #2 s46107), DFCP1 (predesigned, cat. no. 4392429, #1 s28712 and #28714) and non-targeting control siRNA (predesigned, cat. no. 4390844) were purchased from Ambion. For PIK3C3 knockdowns siRNA cat. no. #1 D-005250-02, #2 D-005250-03 and #3 D-005250-04, for RB1CC1/FIP200 knockdowns siRNA cat. no. #1 J-021117-05 and #2 J-021117-06 and non-targeting control siRNA cat. no D-001810-01 were purchased from Dharmacon. Cells at 60% confluency were transfected with 20–40 nM final siRNA concentration using HiPerFect transfection reagent (QIAGEN) according to the manufacturer’s instructions and collected after 48 or 72 h.

### Plasmid constructs

mCherry–P4M-SidM was a gift from T. Balla (Addgene plasmid #51471)^43^, pmCherryLGALS3–mCherry was a gift from H. Meyer (Addgene plasmid #85662)^62^, pmCherry–LC3B and pmCherry–GABARAP were gifts from S. Pankiv^63^, and LAMP1-HaloTag (Ubigene) and VPS13C-Halo were gifts from P. De Camilli (Addgene plasmid #232864)^16^. Using standard molecular cloning techniques, we generated the PI3P mutants Δ2xFYVE and C654S/C770S^18^.

### Immunofluorescence staining and antibodies

Cells seeded on coverslips were fixed in 4% highly purified (EM-grade) paraformaldehyde for 10 min and permeabilized with 0.05% saponin in phosphate-buffered saline (PBS). Cells were washed twice in PBS and once in PBS containing 0.05% saponin before staining with the indicated primary antibodies for 1 h. Previous staining with the secondary antibodies for 1 h, cells were washed three times in PBS containing 0.05% saponin. Cells were mounted in Mowiol containing 2 mg ml^−1^ Hoechst 33342 (Sigma-Aldrich). Rabbit anti-CHMP4B antibody was described previously^64^. Human Galectin-3 Alexa Fluor 488-conjugated antibody (cat. no. IC1154G) was purchased from R&D Systems, mouse anti-ALIX (cat. no. 634502) was purchased from BioLegend, mouse anti-LAMP1 (cat. no. H4A3) was purchased from Developmental Studies Hybridoma Bank, rabbit anti-MOSPD2 (cat. no. HPA003334) was from Merck Life Science (Sigma), rabbit anti-DFCP1 (cat. no. 85156) was purchased from Cell Signalling Technology, rabbit anti-p62 (cat. no. PM045) was purchased from MBL, rabbit anti-VAP-A (cat. no. HPA009174) was purchased from Merck Life Science (Sigma), mouse anti-PI4K2A (cat. no. sc-390026) and mouse anti-Reticulon-4 (cat. no. sc-271878) was purchased from Santa Cruz Biotechnology, rabbit anti-RB1CC1/FIP200 (cat. no. 17250-1-AP) was purchased from Proteintech, rabbit anti-LGALS8 (cat. no. ab109519) was purchased from Abcam, rabbit anti-ATG2A (cat. no. 15011S) was purchased from Cell Signalling Technology, mouse anti-PIK3C3 (cat. no. SAB1406260) was purchased from Merck Life Science (Sigma), mouse anti-β-actin (cat. no. A5316) from Sigma-Aldrich. All secondary antibodies used for immunofluorescence studies and western blotting were obtained from Jacksons ImmunoResearch Laboratories or from Molecular Probes (Life Technologies).

### Immunoblotting

Cells were washed with cold PBS and lysed in 2× sample buffer (125 mM Tris-HCl, pH 6.8, 4% SDS, 20% glycerol, 200 mM dithiothreitol (DTT) and 0.004% bromophenol blue). Whole-cell lysates were subjected to SDS–PAGE on 4–20% gradient gels (Mini-PROTEAN TGX; Bio-Rad). Proteins were transferred to polyvinylidene difluoride (PVDF) membranes (Trans-BlotTurbo LF PVDF, Bio-Rad) followed by blocking in 5% fat-free milk powder and antibody incubation in 5% fat-free milk powder in Tris-buffered saline with 0.1% Tween-20. Membranes incubated with horseradish peroxidase-conjugated antibodies were developed using Clarity Western ECL Substrate Solutions (Bio-Rad) with a ChemiDoc XRS+ imaging system (Bio-Rad).

### FRAP microscopy

For FRAP experiments, U2OS-DFCP1-KO cells stably expressing mNG-DFCP1, mNG-DFCP1T189A and mNG-DFCP1K193A cells were seeded on 35-mm MatTek dishes (MatTek corporation, #P35G-1.5-20-C) and incubated overnight. Cells were then treated with DMSO or LLOMe for indicated time point. The experiment was performed on a Zeiss LSM880 Airyscan FAST confocal microscope equipped with a ×63 oil-immersion objective (Plan-Apochromat ×63/1.40 NA) at 37 °C/ 5% CO_2_. Focus was maintained using the definite focus 2 system and images were acquired using Zeiss Zen software at one image per second. Three pre-bleach images were acquired before bleaching individual mNG-DFCP1-positive structures at 100% laser intensity (30 iterations).

FRAP analysis was carried out following the procedure described previously^65^ using Fiji^66^. For each acquisition, mean fluorescence intensity was measured within three regions of interest (ROIs): the bleached region, a non-bleached reference vesicle within the same cell and a cell-free background region. After subtraction of background intensity from both bleached and non-bleached ROI, a ratio of bleached to non-bleached intensity was calculated for each time point to correct for any fluorescence loss occurred during image acquisition. The resulting value was normalized to the mean pre-bleach intensity, such that the pre-bleach baseline was set to 1 across all conditions.

### Pulse-chasable reporter processing assay for lysosomal autophagic flux (Lysoflux assay) and global autophagic flux

Monitoring of lysosomal autophagic flux was performed using a pulse-chasable reporter processing assay as previously described^45^. In brief, the LGALS3 gene was fused to an EGFP–HaloTag transgene in a Gateway-compatible pENTR vector using standard molecular biology methods. The resulting lentiviral construct, pLenti-PGK-Blast-EGFP-HaloTag-LGALS3, was generated by Gateway LR recombination and subsequently used to establish stable cell lines, as detailed earlier.

For the assay, cells stably expressing EGFP-HaloTag-LGALS3 were incubated in DMEM (10% FBS, 100 U ml^−1^ penicillin and 100 μg ml^−1^ streptomycin) containing 100 nM TMR-conjugated Halo ligand (Promega, G8251) for 30 min. Cells were then treated with DMEM supplemented with 250 μM LLOMe for 0 or 15 min, or 2 h, followed by washing and incubation with fresh DMEM for 4 h. Afterward, cells were washed twice with PBS, scraped and centrifuged at 2,000*g* for 5 min. The cell pellet was resuspended in lysis buffer (20 mM Tris, pH 7.5, 137 mM NaCl, 1 mM MgCl_2_, 1 mM CaCl_2_ and 1% NP-40) supplemented with Halt protease and phosphatase inhibitor cocktail (Thermo Fisher Scientific). Protein concentration was quantified using a BCA assay.

Global autophagic flux was assessed using the same pulse–chase strategy in U2OS WT and DFCP1-KO cells stably expressing HaloTag7-LC3B.

For the in-gel fluorescence imaging, equal protein amounts (15 μg per lane) were resolved on Criterion 4–20% gradient Tris-HCl gels (18-well) and imaged immediately using the ChemiDoc Imaging System (Bio-Rad). Using the same gels, proteins were then transferred to LF PVDF membranes (RTA Transfer kit) using a TransBlot Turbo system. Membranes were blocked in 5% non-fat milk in TBS-Tween (0.05%) for 1 h at room temperature, incubated with primary antibodies in 5% BSA in TBS-Tween (0.05%) overnight at 4 °C, and then with HRP-conjugated anti-rabbit or anti-mouse secondary antibodies in 5% non-fat milk in TBS-Tween (0.05%) for 1 h at room temperature. Band detection was also performed using the ChemiDoc XRS+ imaging system (Bio-Rad).

### *Listeria monocytogenes* infection of U2OS cells

U2OS cells were seeded at a density of 10,000 cells per well onto 18-well chamber slides (DMEM supplemented with 10% FBS, without antibiotics) and incubated overnight at 37 °C in 5% CO_2_*. L.* *monocytogenes* (WT and *Δhly* mutant) was cultured overnight at 37 °C with shaking (220 rpm) in brain heart infusion (BHI) broth. Bacteria were collected by centrifugation at 300*g* for 5 min, the supernatant was discarded, and the pellet was resuspended in antibiotic-free DMEM. The optical density (OD) of the bacterial suspension was measured at 600 nm, with an OD_600_ of 0.1 corresponding to approximately 1 × 10^7^ colony-forming units per ml. Infections were performed at a multiplicity of infection (MOI) of 1 for WT *L.* *monocytogenes*, and MOI 10 for the Δhly mutant, which replicates more slowly. To facilitate bacterial contact and uptake, cells were centrifuged at 800*g* for 5 min at room temperature, followed by incubation at 37 °C for 1 h. Cells were then washed three times with PBS to remove extracellular bacteria and subsequently incubated in fresh DMEM for an additional 4 h before downstream analysis.

### Live-cell microscopy

Cells seeded in MatTek 35-mm Petri dish, 20-mm Microwell no. 1.5 coverglass or on Ibidi m-slide four-well ibiTreat chambers were imaged on a Nikon Eclipse Ti2-E inverted microscope equipped with a CrestOptics X-Light V3 spinning-disk confocal module, a dual camera imaging setup (back-illuminated sCMOS cameras, Photometrics Kinetix) and a CFI Plan Apo ×100 oil NA 1.45 lens. For temperature control during live observation, the microscope stage was kept at 37 °C by a temperature-controlled incubation chamber. Cells were imaged in live-cell imaging buffer (Invitrogen, supplemented with FBS, penicillin–streptomycin, HEPES and Aln-Gln) with 20 mM glucose, at 37 °C. For fixed cells, images were deconvolved using NisElements AR software and processed with Fiji/ImageJ. DeltaVision OMX V4 microscope equipped with three PCO.edge sCMOS cameras, a solid-state light source, a ×60 1.42 NA objective, and a laser-based autofocus was used. Environmental control was provided by a heated stage and an objective heater (20-20 Technologies). Images were deconvolved using softWoRx software and processed in ImageJ/Fiji. Additional fast live-cell imaging was performed on a Zeiss Elyra 7 Lattice-SIM microscope equipped with a Plan-Apochromat ×40/1.4 NA Oil objective and a ×1.6 tube lens, using a Hamamatsu ORCA-Fusion BT sCMOS camera. mNeonGreen and mAzamiGreen were excited at 488 nm (3% laser power); emission was collected in the 495–550 nm band (FS LBF 405/488/561/642 beam splitter) at 100 ms exposure per frame (frame interval 531.40 ms; lateral pixel size, 0.099 µm). Time-lapse acquisitions comprised 80 frames (~215 s total) with z-stacks of five optical sections (z-step, 0.360 µm; total z-range, 1.44 µm). All live-cell imaging was performed at 37 °C with 5% CO_2_. Structured illumination images were processed using the SIM^2^ algorithm with weak auto-sharpness in Zen Blue v.3.12 (Carl Zeiss).

### DFCP1 puncta quantification and correlation analysis

DFCP1 puncta and their colocalization with the ER and lysosomes were analysed using confocal z-stacks of U2OS-DFCP1-KO cells stably expressing mNG-DFCP1. Cells were immunostained with anti-LAMP1 (lysosome marker) and anti-Reticulon-4 (ER marker) under control conditions (no treatment) or after 15 min of LLOMe treatment. Nuclei were counterstained with 4,6-diamidino-2-phenylindole (DAPI). For normalization steps, we first detected the cell outline via a low intensity threshold of the maximum projection in z of the ER staining. Further, we detected nuclei within the image via thresholding of a maximum projection in z of the DAPI signal. Combining these data, we assigned pixels within the mask of the cells (based on the ER stain) to individual cells based on distance to the closest nucleus. This analysis yielded a mask of each cell and a distance map indicating each pixel’s minimal distance to the cell nucleus.

For each segmented cell, we applied the cell mask to each slice of the confocal stack of the DFCP1, ER and lysosome channels and further analysed each cell individually. DFCP1 puncta were identified via thresholding of the confocal stack as those connected pixel that exceeded the intensity of the mean of the maximum projection of DFCP1 within the cell plus 3 × s.d. thereof. Puncta with a volume below 60 pixels were discarded. The cellular organization of ER and lysosomes is not homogeneous but shows typically brighter signal closer to the nucleus. To account for such systematic cellular occurring between DFCP1 puncta closer or further from the nucleus, we normalized all data by an intensity map representing the average intensity of a given channel at a given distance from the nucleus per z-slice. Utilizing the distance map of all pixels relative to the cell nucleus, we calculated the average intensity for each distance from the nucleus in a given channel and z-slice. These intensities are then transferred to the pixels with the respective distance to generate a three-dimensional normalization map capturing the average intensity distribution in the cell. Normalizing each voxel in the confocal stack by this intensity map, we arrived at an intensity scale where a value of 1 indicates that the local intensity is equal to the average intensity of all pixels in the same cell, in the same z-slice, and at the same distance from the nucleus. Values < 1 indicate lower-than-expected intensity, and values > 1 indicate higher-than-expected intensity. With this normalization at hand, we then extracted an image around the centre plane of each DFCP1 punctum in the normalized DFCP1, ER and lysosome channel. Next, we calculated the average intensity as a function of distance from the detected DFCP1 interface, where a distance of zero indicates the outline of the respective DFCP1 punctum, with negative values inside, positive values outside. The same was carried out for the corresponding ER and lysosome images to interrogate colocalization or lack thereof. The individual intensity profiles for each puncta from all cells within one experiment were then aligned and the median intensity across all puncta calculated.

### EM grid seeding and cryo-FIB milling

Gold Quantifoil R2/2 200-mesh EM grids (EMS Q250-AR2) were glow-discharged (30 s, 25 mA) and coated with 0.01% poly-L-lysine (Sigma A-005-M). U2OS cells were trypsinized (0.25%, Thermo Fisher 25-200-056) and seeded at 20,000 cells per grid in an eight-chamber slide (Sigma PEZGS0816). After overnight recovery, cells were treated with 250 µM LLOMe for 20 min. Grids were washed in PBS, blotted with blotting paper and a Teflon sheet (force 10, 8 s) in a Vitrobot Mark IV (37 °C, 90% humidity) and plunge-frozen in liquid ethane. Grids were clipped into notched bases for cryo-focused ion beam (FIB) milling (Thermo Fisher).

Notched grids were loaded into an Aquilos 2 with integrated fluorescence (iFLM). After scanning electron microscopy screening, grids were sputter-coated (30 mA, 10 Pa, 15 s) and GIS-coated (1 min). Auto-transmission electron microscopy milling produced ~200 nm lamellae using stepwise currents: 0.3–0.5 nA to 3 µm, 100 pA to 1 µm, 50 pA to 500 nm and 30 pA to final thickness.

### Cryo-electron tomography data acquisition and processing

Lamellae were transferred from the Aquilos to a 300 kV Titan G2 equipped with a Gatan Quantum K3 detector. Images were acquired in energy-filtered transmission electron microscopy mode (20 eV slit). Autogrids were loaded so FIB pre-tilt was perpendicular to the microscope tilt axis. Whole-grid and medium-magnification montages guided targeting. Tilt series were acquired using a dose-symmetric scheme starting from the 10–15° lamella pre-tilt with 3° increments in groups of two tilts^67^. Nominal defocus varied from –2 to –6 µm (0.25-µm steps). Total dose per series was ~120 e⁻ per Å (ref. ^68^). The conditions for data collection and processing are summarized in Supplementary Table 1.

Scipion was used to facilitate all downstream data processing^68^. In brief, MotionCor3 was used to motion correct tilt series and binning in Fourier space to the physical pixel resolution was applied during correction^69^. CTFfind5 was used for CTF estimation and AreTomo2 or Etomo was used for automatic or manual tilt series alignment and tomogram reconstruction^70–72^. Tomograms were denoised using Isonet2 (ref. ^73^) and a short 3DEM^68^ run to enhance visual contrast. Membranes were segmented by using MemBrain (v.2)^72^. Ribosomes were template matched with PyTOM^74^ using EMD-2938 (ref. ^75^) and back projected to the segmentation with ArtiaX^76^.

### Image analysis and post-processing

Images were analysed in Fiji/ImageJ using custom Python scripts. In brief, images were filtered to remove noise, foci of different proteins were segmented by manual thresholding, and objects were then identified and scored by the ‘Analyse Particles’ function of ImageJ^15^. For live-cell imaging experiments, the value at the time of LLOMe or drug addition (time 0 min) was set to 1, and all subsequent measurements were normalized to this value. Manual thresholds were chosen to detect prominent LLOMe-induced structures; therefore, only spots above a certain intensity were scored. For live-cell videos, foci counts were normalized to the cell number or area (Figs. 1b,d, 3e,i,g, 4b,e–g,i, 5b,c,g and 7a and Extended Data Figs. 1d and 5c,h).

For LysoTracker recovery analysis fully automated analysis using NisElements software, detecting LysoTracker-positive bright puncta per time point, from live-cell imaging videos was applied. Intensity-based thresholding was used for segmentation of cell area. Identical settings for all conditions within one experiment (Figs. 1f,h, 5j and 6g,h and Extended Data Fig. 3i). The analysis of lysosome damage response of various markers based on immunofluorescence confocal microscopy images was fully automated by using NisElements software, detecting bright puncta of indicated marker. Intensity-based thresholding was used for segmentation of cell area and nuclei. Identical settings for all conditions within one experiment (Figs. 2f and 4n and Extended Data Figs. 3k, 4b and 5f). For the analysis of recruitment dynamics of various markers, the semi-automated analysis using NisElements software was applied, detecting bright puncta of indicated markers per cell (transient transfection) or per video consisting of several cells (stable cell lines). For transiently transfected cells, the mean intensity of the marker was measured in the whole cell to indicate the expression level, and the cells were then assigned to a dedicated analysis, set for low, medium or high expression. Identical selection criteria were used for all conditions. Intensity-based thresholding was used for segmentation of cell area or cell numbers were counted manually (Figs. 1j,l, 4k,m, 5h and 6a,e,i and Extended Data Fig. 5d). For the intensity measurement of ER-FYVE and HRS-2XFYVE probes associated with LAMP1, automated analysis using NisElements software was used. Mean fluorescence intensities of probes, LysoTracker or LAMP1 were measured over time in LAMP1-positive areas selected by threshold-based segmentation. Identical settings for all conditions within one experiment (Fig. 1e and Extended Data Fig. 1b). Individual tracking of lysosomes was performed as described previously^77^ (Figs. 1c,d and 6f).

### Statistics and reproducibility

The number of individual experiments and the number of cells analysed are indicated in the figure legends. We tested our datasets for normal distribution and chose an appropriate test accordingly using GraphPad Prism v.10. No statistical methods were used to predetermine sample sizes. The statistical tests performed for each experiment are specified in the figure legends. *P* values are indicated for each experiment and *P* values <0.05 were regarded as significant. Data collection and analysis were not performed blind to the conditions of the experiments and no randomization was applied. Data were excluded only for technical reasons, including loss of focus, excessive photobleaching or phototoxicity during live-cell imaging experiments. To ensure the reproducibility of the data presented, experiments were performed by different researchers and different groups.

### Reporting summary

Further information on research design is available in the Nature Portfolio Reporting Summary linked to this article.

## Online content

Any methods, additional references, Nature Portfolio reporting summaries, source data, extended data, supplementary information, acknowledgements, peer review information; details of author contributions and competing interests; and statements of data and code availability are available at https://doi.org/10.1038/s41556-026-02062-z.

## Supplementary information

Reporting Summary

Supplementary Video 1 Video for Extended Fig. 1a,b. Live-cell imaging of HeLa cells transiently expressing PI3P probe EGFP-2xFYVE^HRS^ (green) and mCherry–LAMP1 (magenta) treated with 250 μM LLOMe. Images were acquired every 20 s for 30 min on a Deltavision OMX V4 microscope. Scale bar, 5 µm.

Supplementary Video 2 Video for Fig. 1a. Live-cell imaging of U2OS cells transiently expressing ER-targeted PI3P probe GFP-ER-FYVE-WDFY1 treated with 250 μM LLOMe as indicated in the video. Images were acquired every 20 s for 30 min on a Deltavision OMX V4 microscope. Scale bar, 5 µm.

Supplementary Video 3 Video for Fig. 1b. Live-cell imaging of U2OS cells stably expressing GFP-ER-FYVE-WDFY1 probe (green) with the ER marker VVAPB–mCherry (magenta) following 250 µM LLOMe treatment. Cells were pre-treated with 75 nM LysoTracker Deep Red for 30 min to monitor lysosome damage. Scale bar, 5 µm.

Supplementary Video 4 Video for Fig. 1c. Live-cell imaging of single lysosomes in U2OS cells stably expressing GFP-ER-FYVE-WDFY1 probe (green) with the ER marker VAP-B–mCherry (magenta) following 250 µM LLOMe treatment. Cells were pre-treated with 75 nM LysoTracker Deep Red for 30 min to monitor lysosome damage (blue). Scale bar, 500 nm.

Supplementary Video 5 Video for Extended Fig. 1c,d. Live-cell imaging of U2OS cells transiently expressing ER-targeted PI3P probe GFP-ER-FYVE-WDFY1 treated with SAR405 as indicated in the video. Images were acquired every 20 s for 30 min on a Deltavision OMX V4 microscope. Scale bar, 5 µm.

Supplementary Video 6 Video for Fig. 1d. Live-cell imaging of U2OS cells transiently expressing ER-targeted PI3P probe GFP-ER-FYVE-WDFY1 and mCherry–LAMP1 treated with 250 µM LLOMe and 1 μM SAR405 as indicated in the video. Images were acquired every 10 s for 30 mins. Scale bar, 500 nm.

Supplementary Video 7 Video for Fig. 1f and Extended Fig. 1e. HeLa cells were pre-treated with 75 nM LysoTracker Deep Red for 30 min before the addition of: a) 250 µM LLOMe, b) 1 µM SAR405 (pre-incubation for 15 min) or c) SAR405 (pre-incubation for 15 min) and LLOMe (times indicated in the videos). While the decrease in the number of LysoTracker foci is quickly recovered in HeLa cells treated with LLOMe, there is a severe impairment in lysosomal repair in HeLa cells co-treated with SAR405 and LLOMe. Number of LysoTracker foci remained unchanged in cells treated with SAR405 alone. Images were acquired on a Nikon Crest microscope. Scale bar, 5 µm.

Supplementary Video 8 Video for Fig. 1f and Extended Fig. 1e. HeLa cells were pre-treated with 75 nM LysoTracker Deep Red for 30 min before the addition of: a) 250 µM LLOMe, b) 1 µM SAR405 (pre-incubation for 15 min) or c) SAR405 (pre-incubation for 15 min) and LLOMe (times indicated in the videos). While the decrease in the number of LysoTracker foci is quickly recovered in HeLa cells treated with LLOMe, there is a severe impairment in lysosomal repair in HeLa cells co-treated with SAR405 and LLOMe. Number of LysoTracker foci remained unchanged in cells treated with SAR405 alone. Images were acquired on a Nikon Crest microscope. Scale bar, 5 µm.

Supplementary Video 9 Video for Fig. 1f and Extended Fig. 1e. HeLa cells were pre-treated with 75 nM LysoTracker Deep Red for 30 min before the addition of: a) 250 µM LLOMe, b) 1 µM SAR405 (pre-incubation for 15 min) or c) SAR405 (pre-incubation for 15 min) and LLOMe (times indicated in the videos). While the decrease in the number of LysoTracker foci is quickly recovered in HeLa cells treated with LLOMe, there is a severe impairment in lysosomal repair in HeLa cells co-treated with SAR405 and LLOMe. Number of LysoTracker foci remained unchanged in cells treated with SAR405 alone. Images were acquired on a Nikon Crest microscope. Scale bar, 5 µm.

Supplementary Video 10 Video for Fig. 2a,b. Live-cell imaging of U2OS-DFCP1-KO cells stably expressing mNG-DFCP1 treated with 250 μM LLOMe. Images were acquired every 30 s for 30 min on a Deltavision OMX V4 microscope. Scale bar, 5 µm.

Supplementary Video 11 U2OS-DFCP1-KO cells stably expressing mNG-DFCP1 were transfected with LGALS3–mCherry and imaged using a Lattice-SIM microscope in the presence of LLOMe in 5-s time intervals.

Supplementary Video 12 Video for Fig.3a. Live-cell imaging of U2OS-mNG-DFCP1 cells (green) transiently expressing LAMP1 (magenta). Images were taken under a Nikon CSU-W1 SoRa microscope in the presence of LLOMe following 6.52 s time-lapse. Scale bar, 1 µm.

Supplementary Video 13 Video for Fig. 3b. Live-cell imaging of U2OS-mNG-DFCP1 cells (green) transiently expressing SEC61β. The imaging started right after addition of 250 µM LLOMe. The interval between the frames is 6.09 s. For SEC61β the LUT used was JDM_Greys g = 1.25. Scale bar, 1 µm.

Supplementary Video 14 Video for Fig. 3h,i. Live-cell imaging of U2OS-mEGFP-DFCP1 and U2OS-mEGFP-DFCP1ΔER cells treated with LLOMe as indicated in the video. Images were acquired every 20 s for 30 min on a Deltavision OMX V4 microscope. Scale bar, 5 µm.

Supplementary Video 15 Video for Fig. 4d,e. Live-cell imaging of U2OS-mNG-DFCP1 cells treated with LLOMe, ML-SI3 or ML-SI3 + LLOMe as indicated in the videos. Images were acquired every 20 s for 30 min on a Deltavision OMX V4 microscope. Scale bar, 5 µm.

Supplementary Video 16 Video for Fig. 4f. Live-cell imaging of full-length DFCP1 cells pre-treated with Thapsigargin for 30 min and/or LLOMe as indicated in the videos. Images were acquired every 20 s for 30 min on a Deltavision OMX V4 microscope. Scale bar, 5 µm.

Supplementary Video 17 Video for Fig. 4g. Live-cell imaging of a mutant DFCP1ΔER cells pre-treated with Thapsigargin for 30 min and/or LLOMe as indicated in the videos. Images were acquired every 20 s for 30 min on a Deltavision OMX V4 microscope. Scale bar, 5 µm.

Supplementary Video 18 Video for Fig. 4h,i. U2OS-DFCP1-KO cell stably expressing mNG-DFCP1. DFCP1 does not accumulate in cells treated with SAR405+LLOMe when compared to LLOMe control. Images were acquired every 20 s for 30 min on a Deltavision OMX V4 microscope. Scale bar, 5 µm.

Supplementary Video 19 Video for Fig. 5a,b. WT and DFCP1-KO cells were pre-treated with 75 nM LysoTracker Deep Red for 30 min before the addition of 250 µM LLOMe as indicated in the videos. Images were acquired every 1 min for 2 h on a Nikon Crest microscope. Scale bar, 5 µm.

Supplementary Video 20 Video for Fig. 5c and Extended Fig. 4a. Live-cell imaging experiments monitoring LysoTracker recovery after induced lysosomal damage using 250 μM LLOMe. U2OS-mEGFP-DFCP1 and U2OS-mEGFP-DFCP1ΔER cells were pre-treated with 75 nM LysoTracker Deep Red for 30 min before the addition of LLOMe. Images were acquired every 1 min for 2 h on a Nikon Crest microscope. Scale bar, 5 µm.

Supplementary Video 21 Video for Fig. 6c. Live-cell imaging of U2OS-DFCP1-KO cells stably expressing mNG-DFCP1 and transiently transfected with VPS13C-Halo (magenta) in the presence of LLOMe. Cells were imaged every 10 s for 30 min. Frame 1 contains arrow pointing to the event shown in insets. Scale bar, 5 µm, scale bar insets, 2 µm.

Supplementary Video 22 Video for Fig. 6d,e. U2OS and U2OS-DFCP1-KO cells transiently transfected with VPS13C-Halo and imaged every 10 s for 30 min. LLOMe was added as indicated. Scale bar, 5 µm.

Supplementary Video 23 Video for Fig. 7a. Live-cell imaging experiments of U2OS-mNG-DFCP1 cells treated with LLOMe or Torin1+LLOMe. Cells were pre-incubated with Torin1 for 2 h before imaging. Images were acquired every 20 s for 30 min on a Deltavision OMX V4 microscope. Scale bar, 5 µm.

Supplementary Table 1 Cryo-EM data collection, refinement and validation statistics.

Supplementary Table 2 Light microscopy reporting tables.

## Source data

Source Data Figs. 1–8 and Extended Data Figs. 1, 3–5 Statistical source data.

Source Data Extended Data Figs. 1–3, 6 and 7 Unprocessed western blots.

## Data Availability

All data supporting the findings of this study are available from the corresponding authors on reasonable request. Source data are provided with this paper.

## Code availability

All data analysis and visualization were performed using the software packages described in the Reporting Summary. Scripts used for the DFCP1 puncta quantification and correlation analysis in Fig. 2h are available at Zenodo at https://doi.org/10.5281/zenodo.21619487 (ref. ^78^).
