# Supplementary material for: DFCP1-containing ER microdomains mediate lysosomal membrane repair
Source: Nat Cell Biol. 2026 Sep 15;28(10):2036–53. doi: 10.1038/s41556-026-02062-z (PMC13661268; doi:10.1038/s41556-026-02062-z)
Supplement: Supplementary file 1 [file 41556_2026_2062_MOESM1_ESM.pdf]

nature portfolio

Corresponding author(s): Maja Radulovic
Harald Stenmark

Last updated by author(s): 19/6/26

Reporting Summary

Nature Portfolio wishes to improve the reproducibility of the work that we publish. This form provides structure for consistency and transparency in reporting. For further information on Nature Portfolio policies, see our Editorial Policies and the Editorial Policy Checklist.

Statistics

For all statistical analyses, confirm that the following items are present in the figure legend, table legend, main text, or Methods section.

n/a	Confirmed	
☐	☒	The exact sample size (*n*) for each experimental group/condition, given as a discrete number and unit of measurement
☐	☒	A statement on whether measurements were taken from distinct samples or whether the same sample was measured repeatedly
☐	☒	The statistical test(s) used AND whether they are one- or two-sided<br>*Only common tests should be described solely by name; describe more complex techniques in the Methods section.*
☒	☐	A description of all covariates tested
☐	☒	A description of any assumptions or corrections, such as tests of normality and adjustment for multiple comparisons
☐	☒	A full description of the statistical parameters including central tendency (e.g. means) or other basic estimates (e.g. regression coefficient) AND variation (e.g. standard deviation) or associated estimates of uncertainty (e.g. confidence intervals)
☐	☒	For null hypothesis testing, the test statistic (e.g. *F*, *t*, *r*) with confidence intervals, effect sizes, degrees of freedom and *P* value noted<br>*Give P values as exact values whenever suitable.*
☒	☐	For Bayesian analysis, information on the choice of priors and Markov chain Monte Carlo settings
☒	☐	For hierarchical and complex designs, identification of the appropriate level for tests and full reporting of outcomes
☒	☐	Estimates of effect sizes (e.g. Cohen's *d*, Pearson's *r*), indicating how they were calculated

Our web collection on statistics for biologists contains articles on many of the points above.

Software and code

Policy information about availability of computer code

Data collection	Image acquisition was performed using Softworx software 7.0 (GE Healthcare), Zen Software 2012 (Zeiss), NIS-Elements 5.30.04 and 5.42.02 (Nikon). For cryo-electron tomography data was collected on 300 kV Titan G2 equipped with a Gatan Quantum K3 detector.
Data analysis	Deconvolution was performed using Softworx 7.0 software and NIS-Elements.<br>Imaging data was analysed using FIJI/ImageJ.<br>Data analysis, statistics and plotting was performed using GraphPad Prism (10.0)<br>Code for analyses used in Figure 2H can be found here https://doi.org/10.5281/zenodo.21619487

For manuscripts utilizing custom algorithms or software that are central to the research but not yet described in published literature, software must be made available to editors and reviewers. We strongly encourage code deposition in a community repository (e.g. GitHub). See the Nature Portfolio guidelines for submitting code & software for further information.

Data

Policy information about availability of data

All manuscripts must include a data availability statement. This statement should provide the following information, where applicable:

- Accession codes, unique identifiers, or web links for publicly available datasets
- A description of any restrictions on data availability
- For clinical datasets or third party data, please ensure that the statement adheres to our policy

All data shown and used to generate plots , as well as detailed statistical information, it is contained in the manuscript or is present in the source data file. Uncropped western blots and gels are shown in source data files. Underlying image data are available from the corresponding authors (H.S. and M.R.) upon request. A data availability statement is included in the text.

Research involving human participants, their data, or biological material

Policy information about studies with human participants or human data. See also policy information about sex, gender (identity/presentation), and sexual orientation and race, ethnicity and racism.

Reporting on sex and gender	not applicable
Reporting on race, ethnicity, or other socially relevant groupings	not apllicable
Population characteristics	not applicable
Recruitment	not applicable
Ethics oversight	not applicable

Note that full information on the approval of the study protocol must also be provided in the manuscript.

Field-specific reporting

Please select the one below that is the best fit for your research. If you are not sure, read the appropriate sections before making your selection.

☒ Life sciences ☐ Behavioural & social sciences ☐ Ecological, evolutionary & environmental sciences

For a reference copy of the document with all sections, see nature.com/documents/nr-reporting-summary-flat.pdf

Life sciences study design

All studies must disclose on these points even when the disclosure is negative.

Sample size	No statistical methods were used to pre-determine the correct sample size. All statistical calculations were derived from at least 3 biological replicates. For microscopy-based data, each biological repeat included sufficient cell numbers to ensure that the effects could be robustly measured and was repeated at least 3 times.
Data exclusions	Collected data were only excluded due to technical reasons such as law knockdown efficiency. For live-cell imaging data was excluded prior to analysis if it showed strong photobleaching or the imaged cells showed phototoxicity effects.
Replication	Data was replicated in at least 3 independent experiments and was reproduced by independent researchers.
Randomization	This study used cultured cell lines which are homogeneous and isogenic. Therefore, no randomization was used prior to treatment.
Blinding	Investigators were not blinded since blinding is technically and practically not feasible in this type of cell biology study. Key experiments were repeated by independent researchers and scientific groups. For live cell imaging, cells were selected before their behavior was known. Key conclusions were drawn by at least two independent researchers.

Reporting for specific materials, systems and methods

We require information from authors about some types of materials, experimental systems and methods used in many studies. Here, indicate whether each material, system or method listed is relevant to your study. If you are not sure if a list item applies to your research, read the appropriate section before selecting a response.

Materials & experimental systems

n/a	Involved in the study
☐	☒ Antibodies
☐	☒ Eukaryotic cell lines
☒	☐ Palaeontology and archaeology
☒	☐ Animals and other organisms
☒	☐ Clinical data
☒	☐ Dual use research of concern
☒	☐ Plants

Methods

n/a	Involved in the study
☒	☐ ChIP-seq
☒	☐ Flow cytometry
☒	☐ MRI-based neuroimaging

Antibodies

Antibodies used

Human Galectin-3 Alexa Fluor 488-conjugated antibody (cat. no. IC1154G) was purchased from R&D Systems IF 1:100
mouse anti-ALIX (cat. no. 634502) was purchased from BioLegend IF 1:100
mouse anti-LAMP1 (cat. no. H4A3) was purchased from Developmental Studies Hybridoma Bank IF 1:100
rabbit-MOSPD2 (cat. no HPA003334) was from Merck Life Science (Sigma) IF 1:100, WB 1:1000
rabbit anti-LC3 (cat. no. PM036) from MBL International Corporation IF 1:400, WB 1:1000
rabbit anti-DFCP1 (cat. no. 85156) was purchased from Cell Signalling Technology WB 1:1000
rabbit anti-p62 (cat. no. PM045) was purchased from MBL IF 1:500
rabbit anti-VAPA (cat. no. HPA009174) was purchased from Merck Life Science (Sigma) WB 1:1000
mouse anti-PI4K2A (cat. no. sc-390026) as purchased from Santa Cruz WB 1:1000
mouse anti-Reticulon4 (cat. no. sc-271878) was purchased from Santa Cruz IF 1:100
rabbit anti-RB1CC1/FIP200 (cat. no. 17250-1-AP) was purchased from Proteintech WB 1:500
rabbit anti-LGALS8 (cat. no. ab109519) was purchased from Abcam IF 1:100
mouse anti-PIK3C3 (cat. no. SAB1406260) was purchased from Merck Life Science (Sigma) WB 1:1000
mouse anti-β-actin (cat. no. A5316) from Sigma-Aldrich WB 1:20000

Validation

Antibodies were validated using either siRNA depletion, knockout or functional tests.

Eukaryotic cell lines

Policy information about cell lines and Sex and Gender in Research

Cell line source(s)

U2OS cells (ATCC: HTB-96), female origin
HeLa cells (Obtained from Kyoto, Japan), female origin

Authentication

Cells were authenticated by the Genotyping core facility at the Oslo University Hospital using Powerplex16 assays.

Mycoplasma contamination

Cell lines were verified to be mycoplasma-free and were regularly tested for mycoplasma contamination. All cell lines were negative for mycoplasma infection.

Commonly misidentified lines
(See ICLAC register)

none

Plants

Seed stocks

not applicable

Novel plant genotypes

not applicable

Authentication

not applicable
