# Supplementary material for: DFCP1-containing ER microdomains mediate lysosomal membrane repair
Source: Nat Cell Biol. 2026 Sep 15;28(10):2036–53. doi: 10.1038/s41556-026-02062-z (PMC13661268; doi:10.1038/s41556-026-02062-z)

Extended Figure 1F

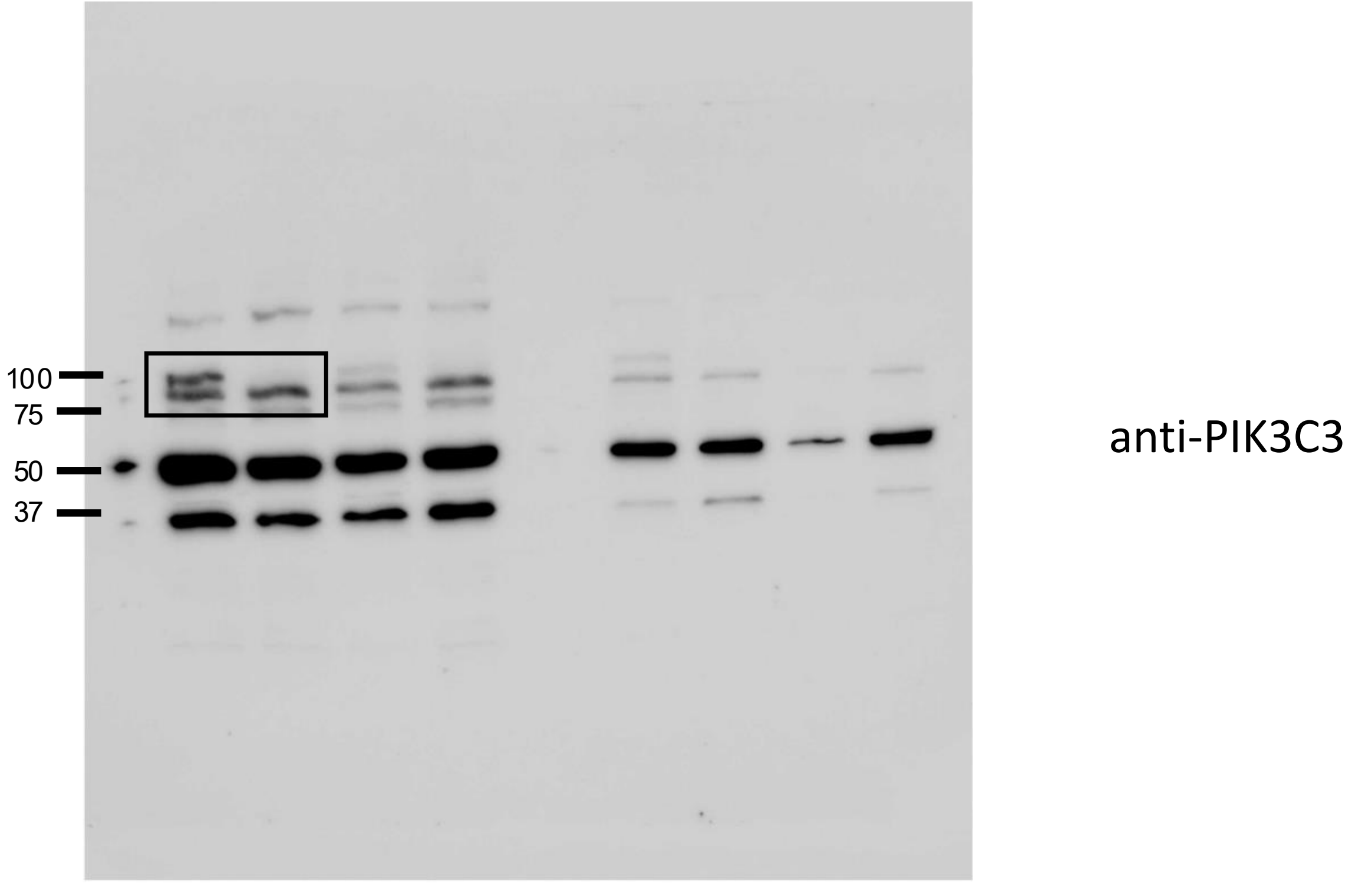

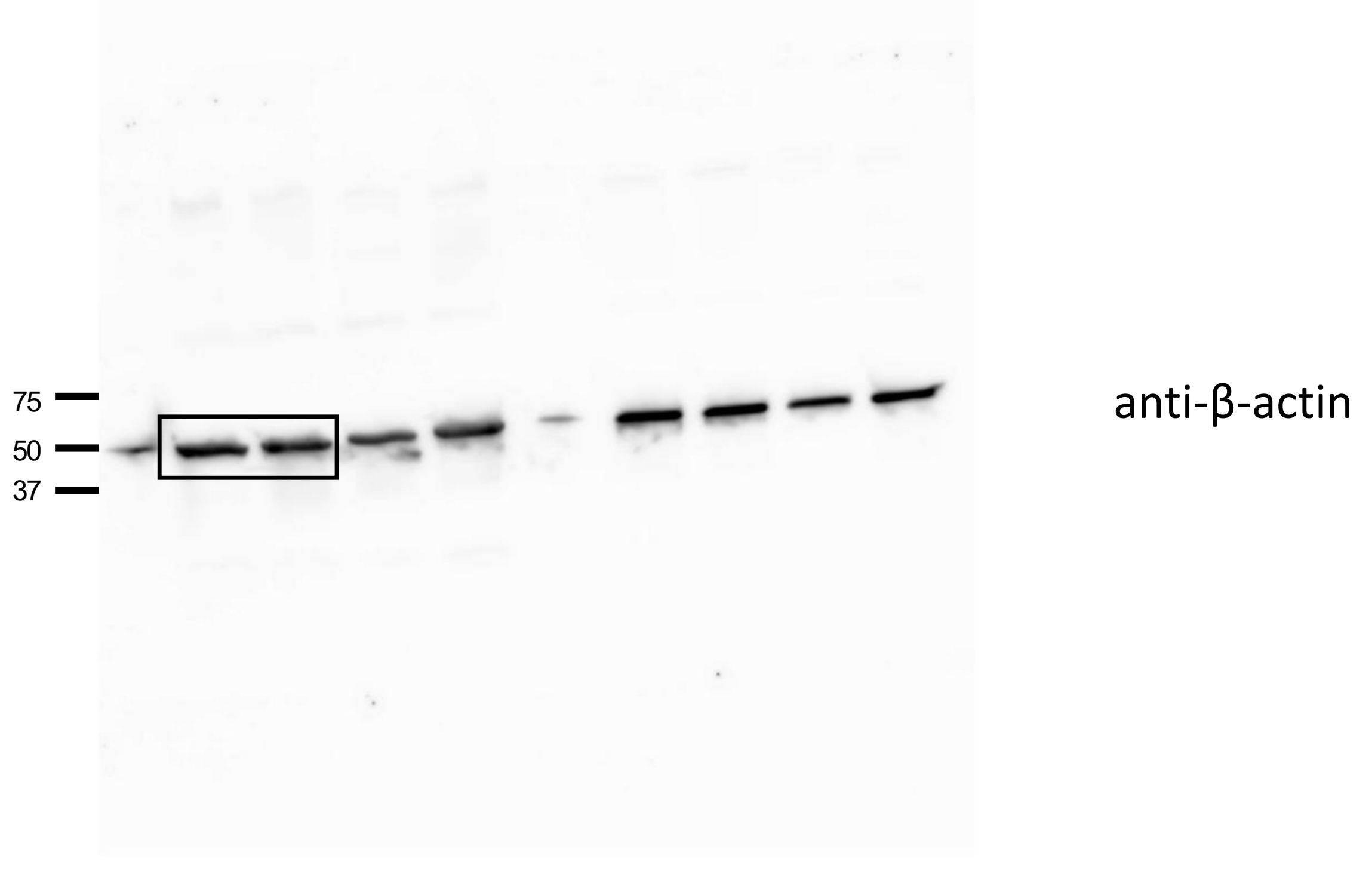

Extended Figure 2B

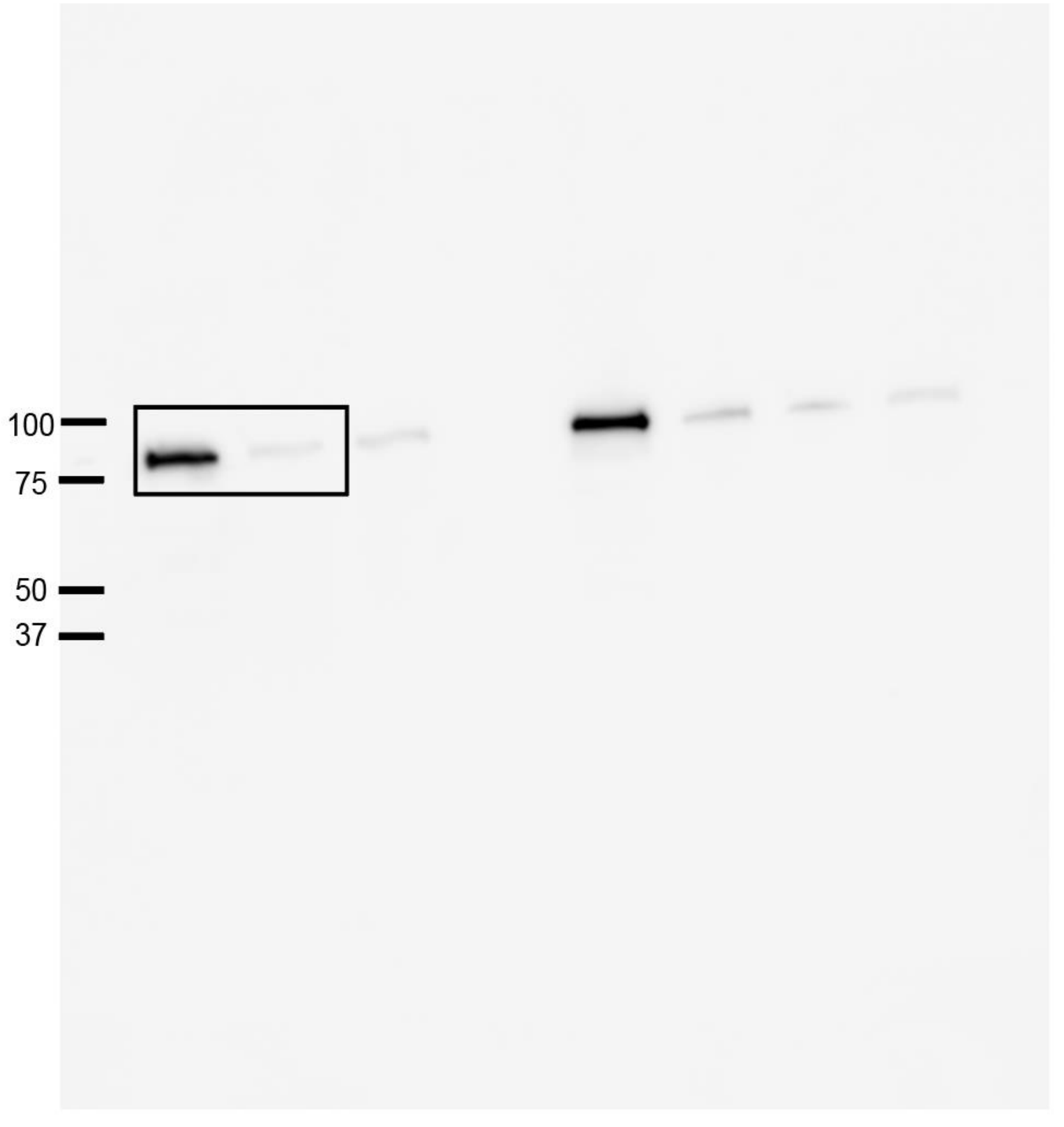

anti-DFCP1

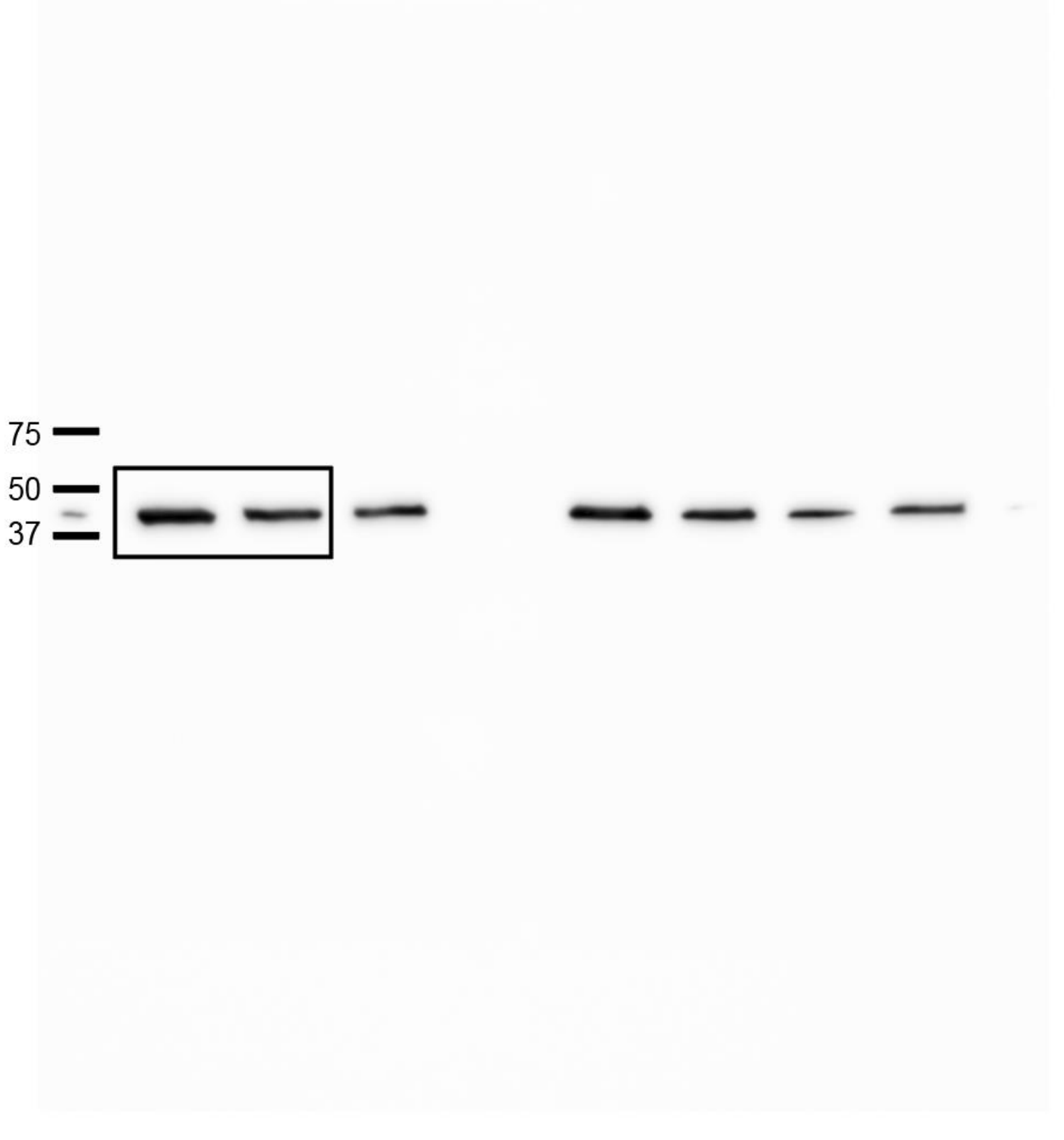

anti-β-actin

Extended Figure 3F

75
50

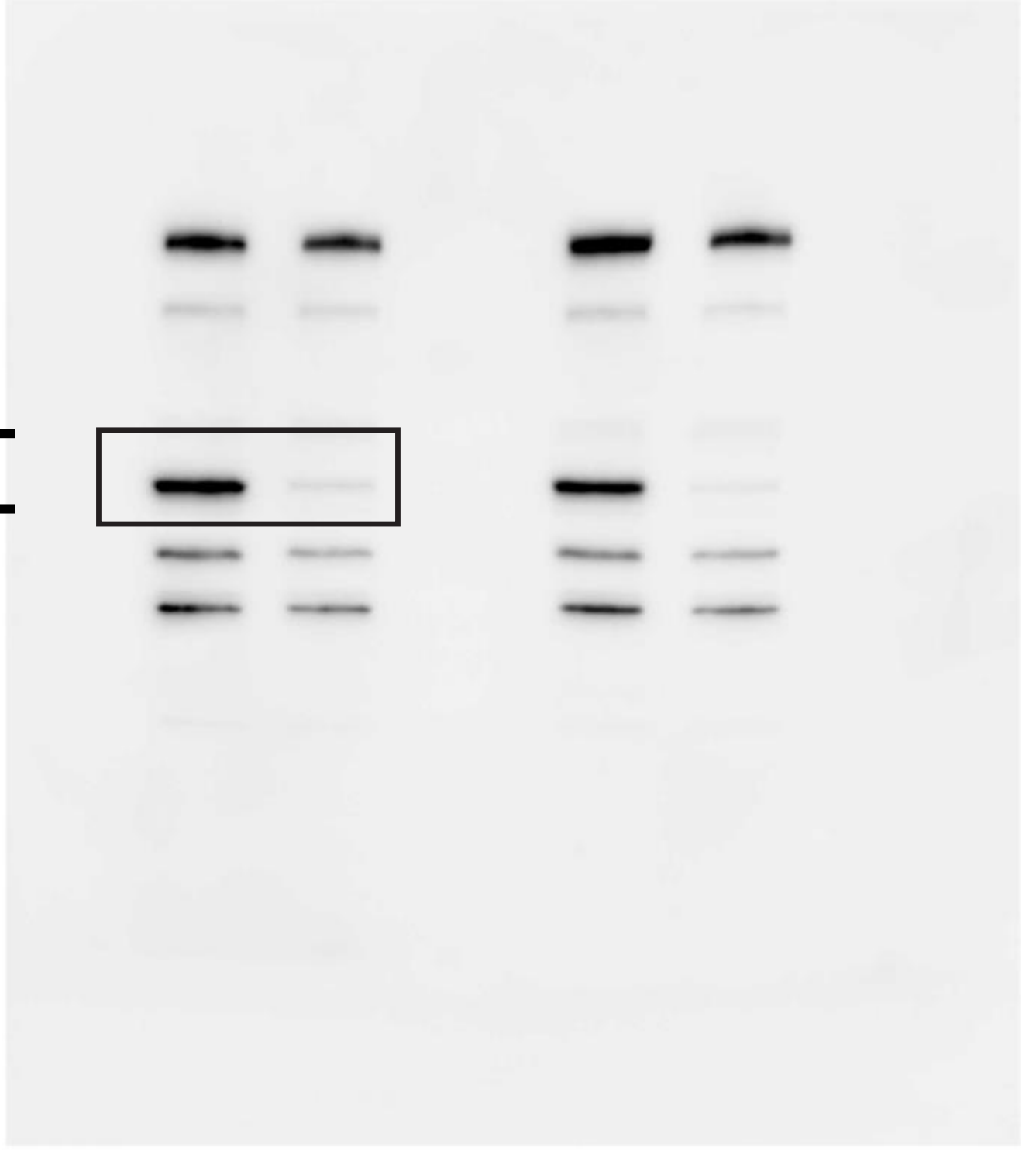

anti-MOSPD2

50
37

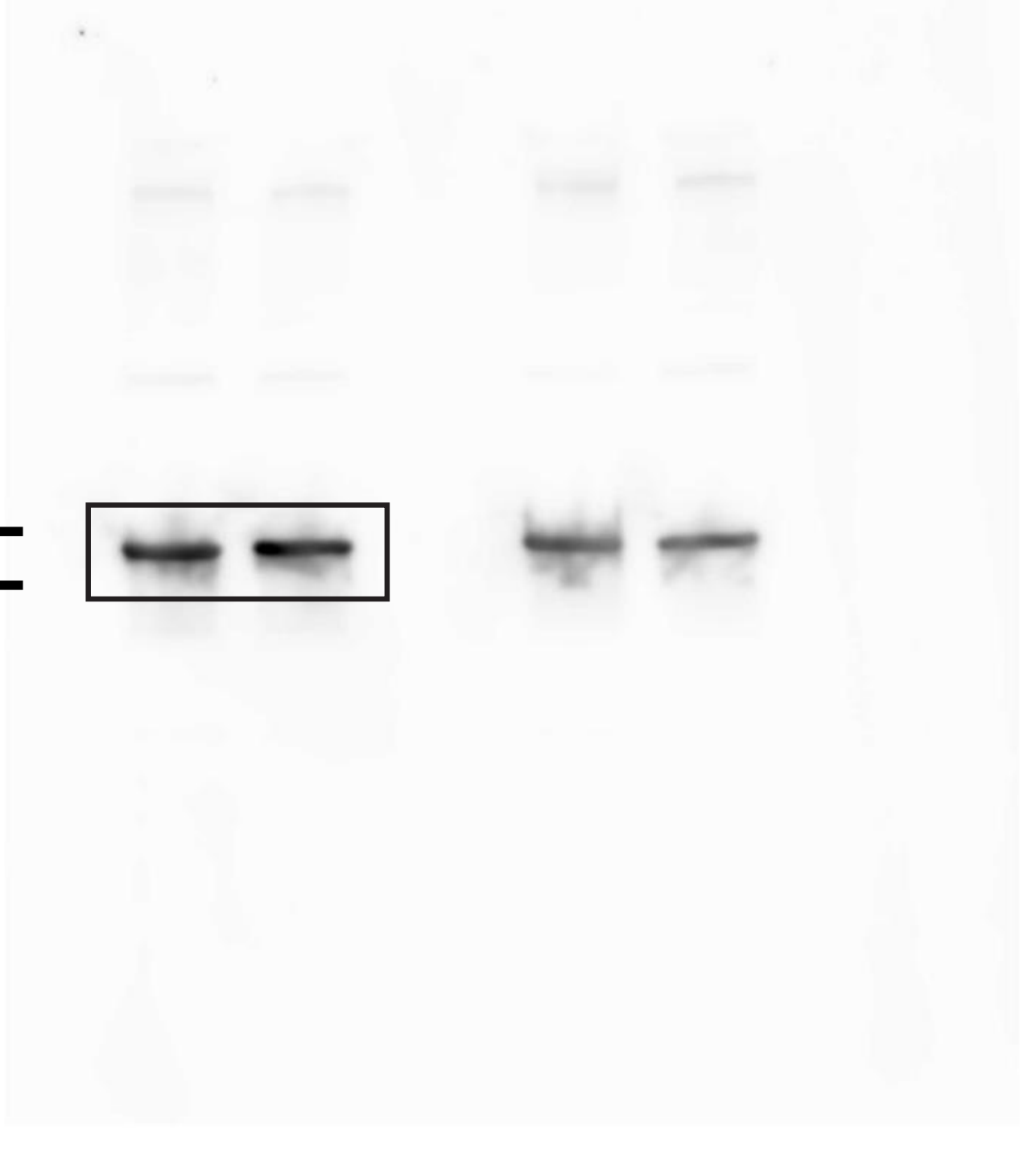

anti-β-actin

Extended Figure 3G

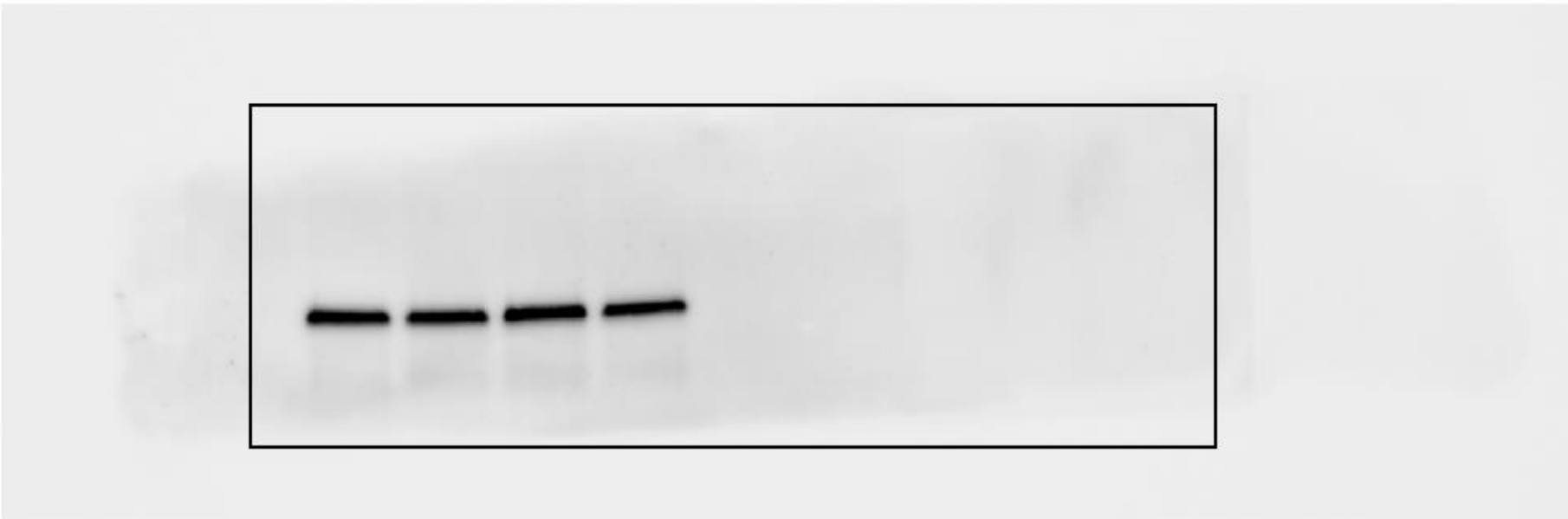

anti-DFCP1

In-gel fluorescence

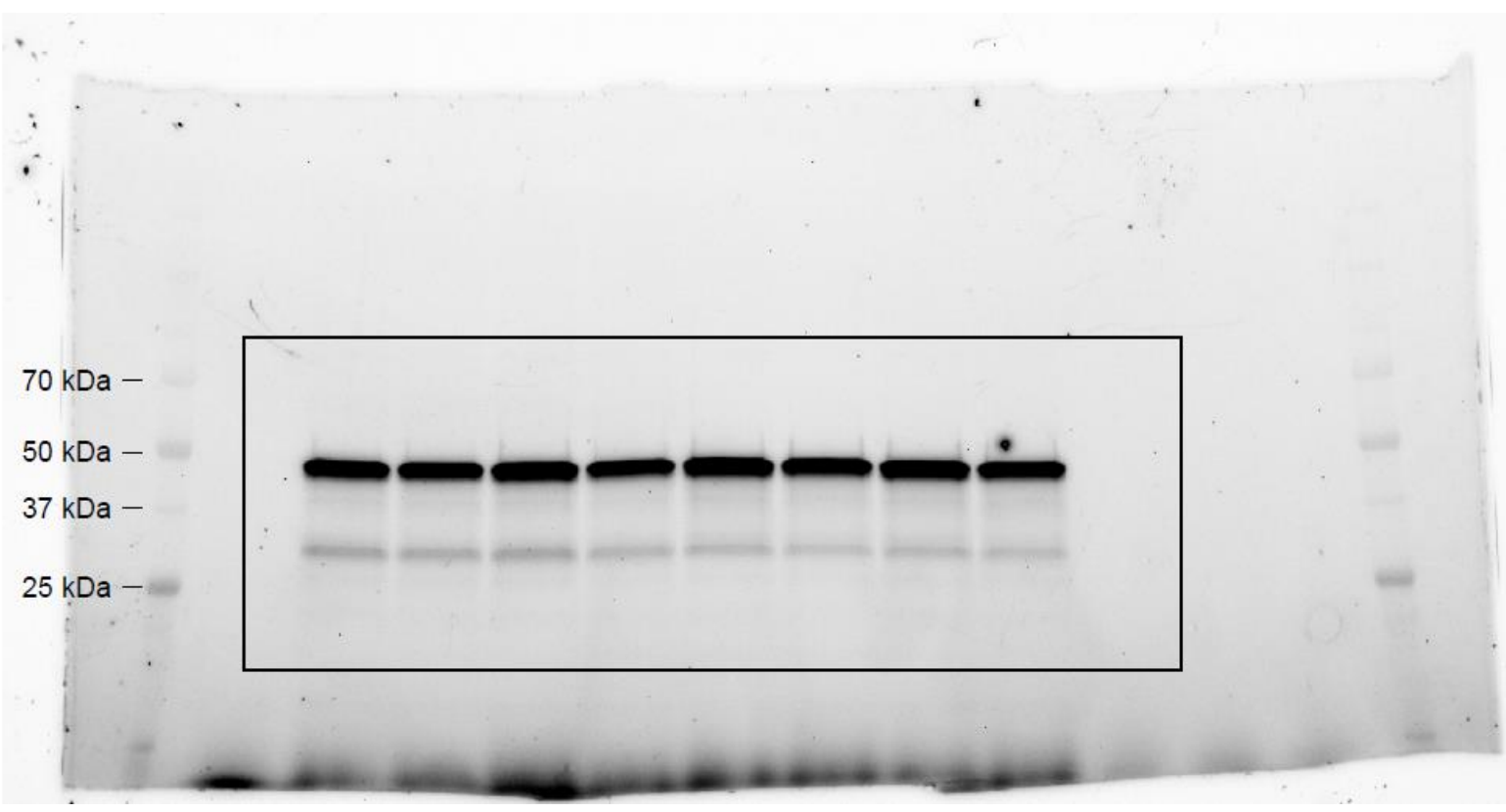

Stain-free gel

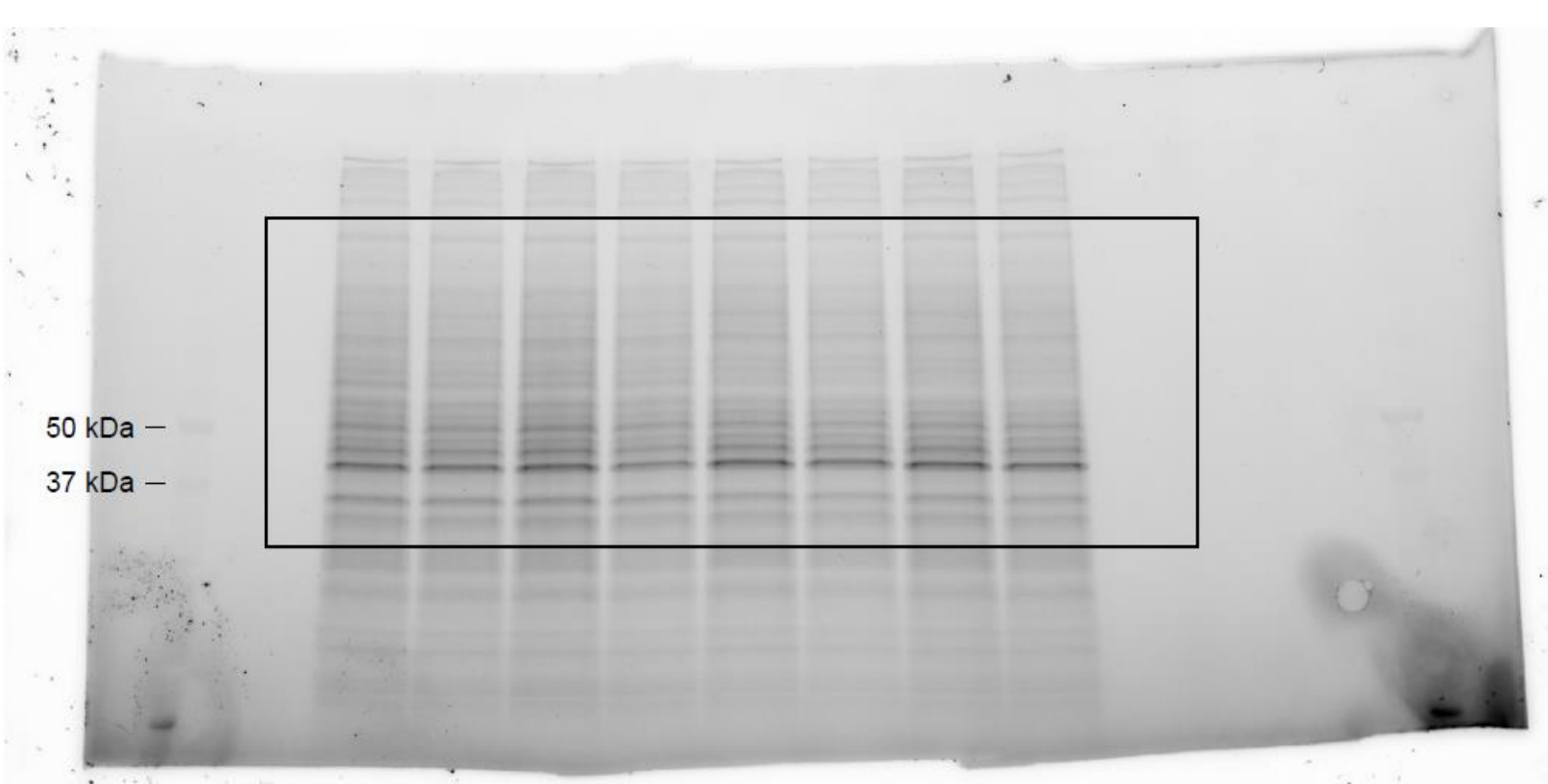

Extended Figure 3L

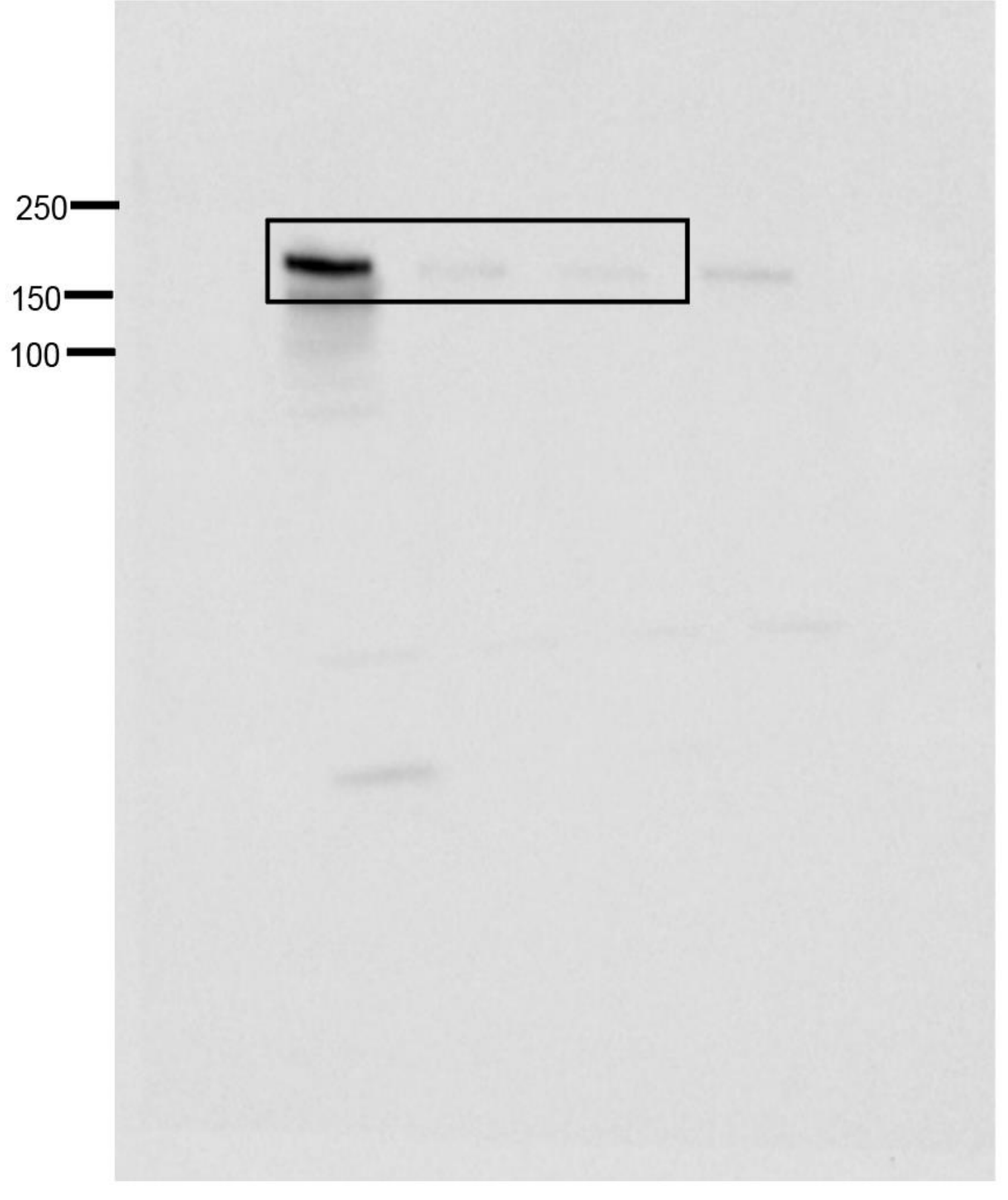

anti-FIP200

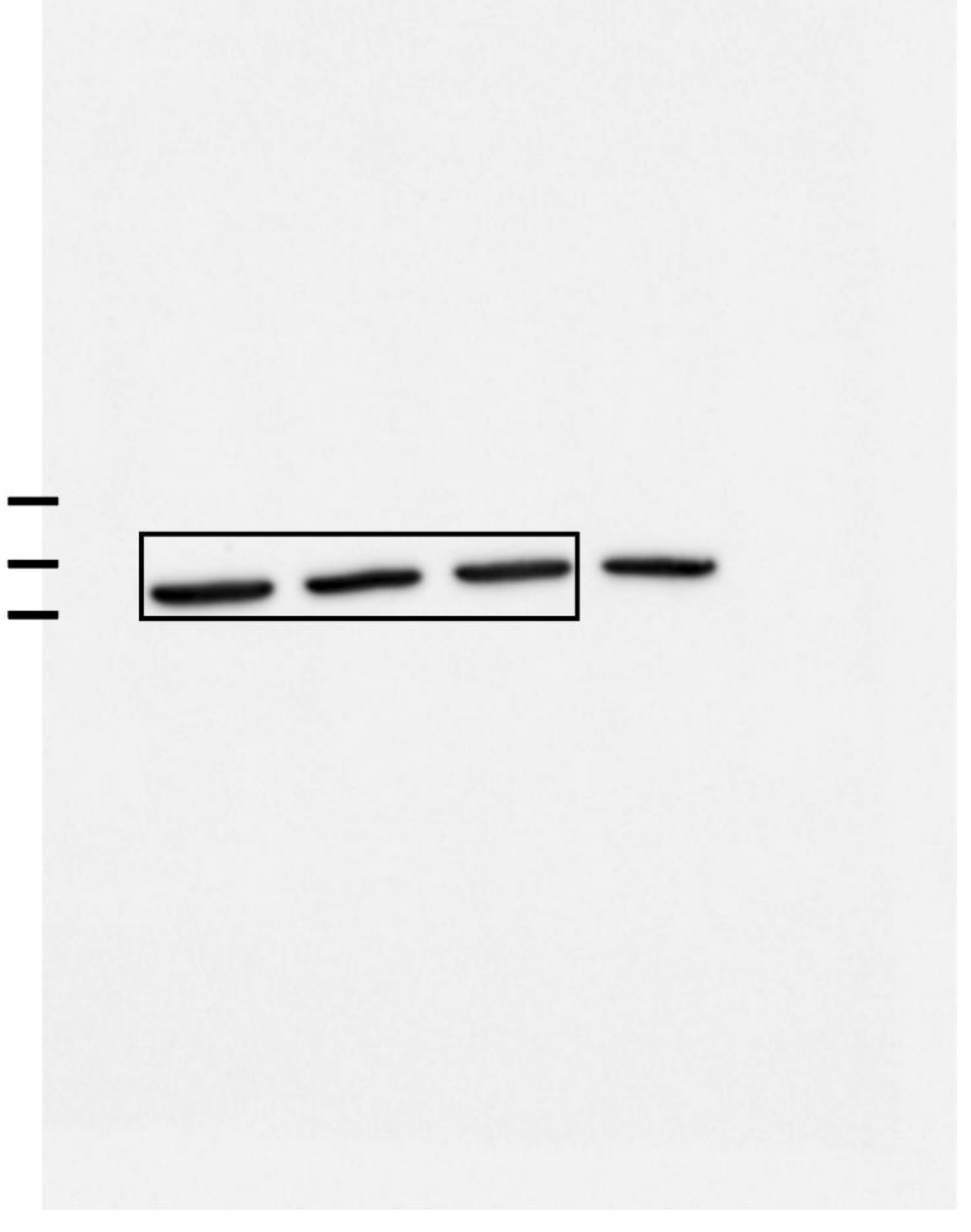

anti-β-actin

Extended Figure 6A

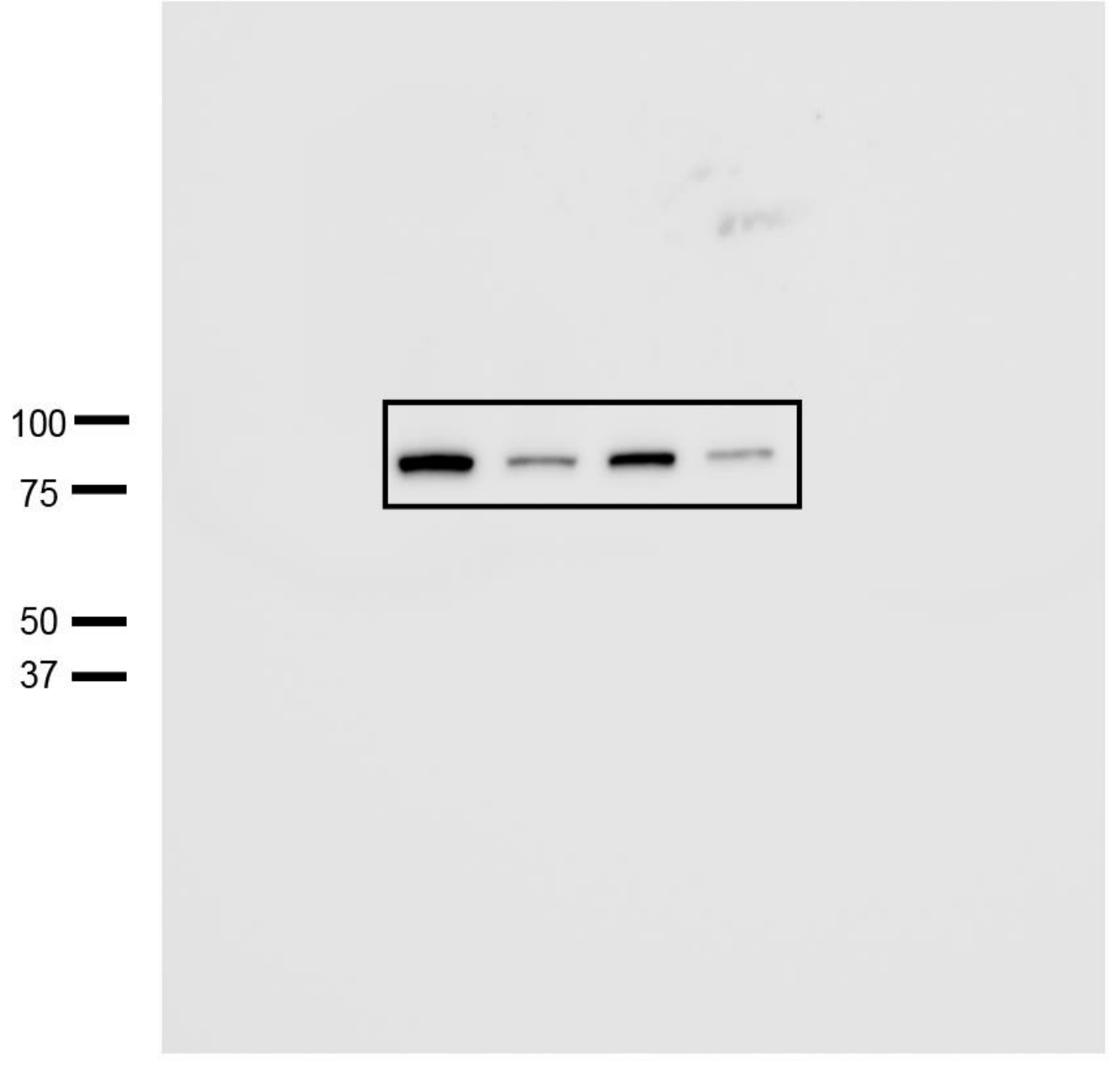

anti-DFCP1

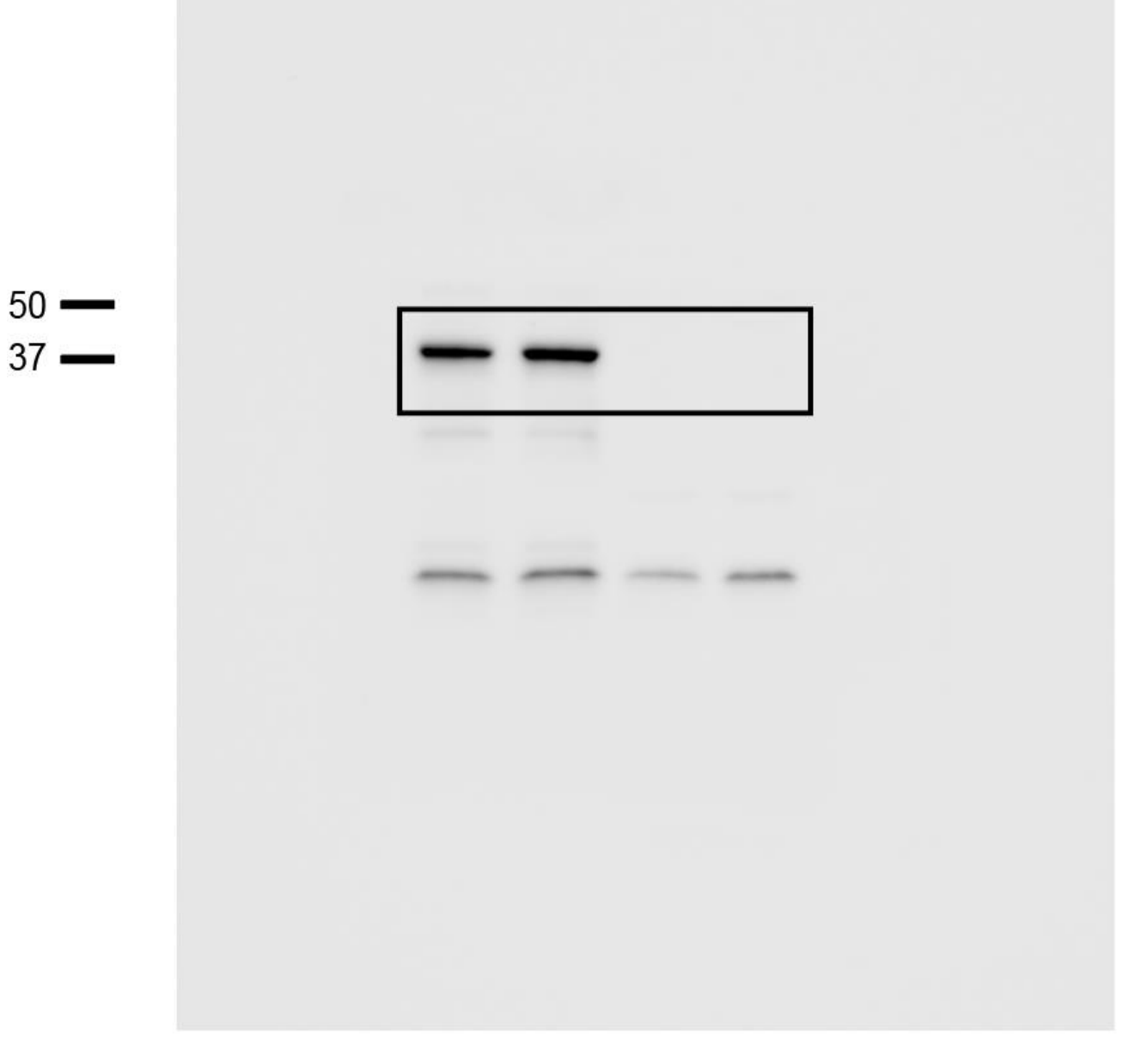

anti-PI4K2A

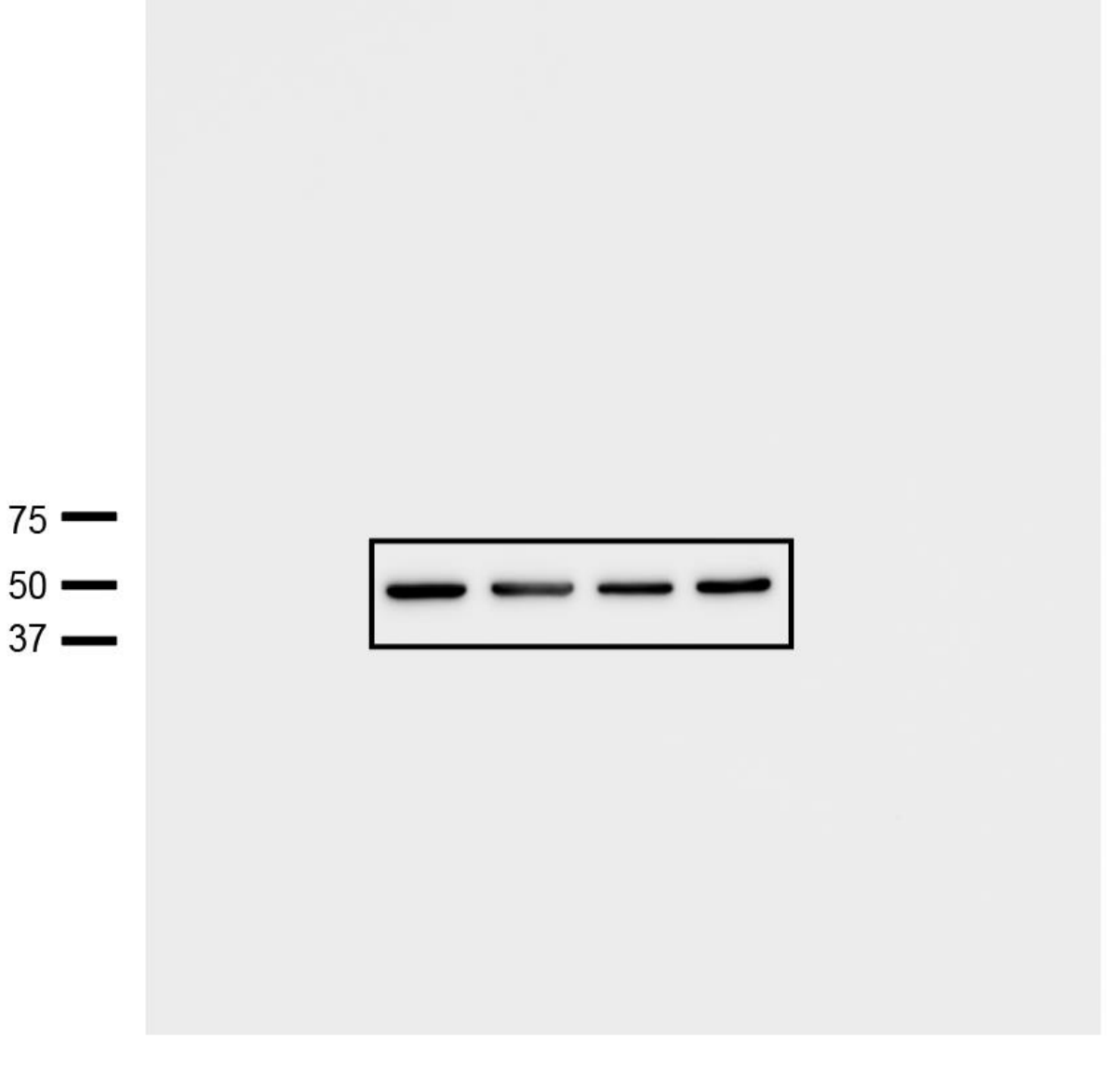

anti-β-actin

Extended Figure 6C

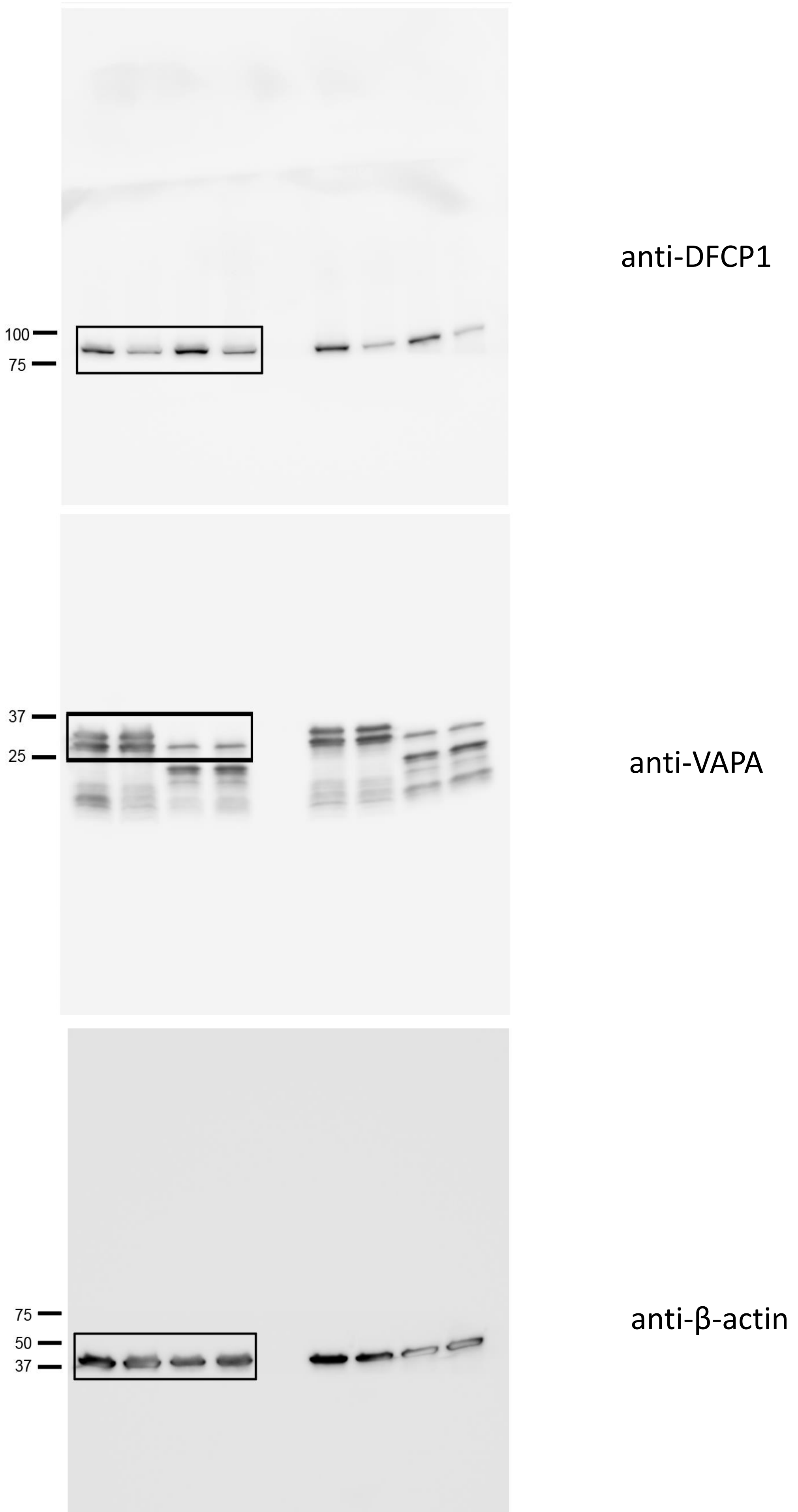

Extended Figure 7A

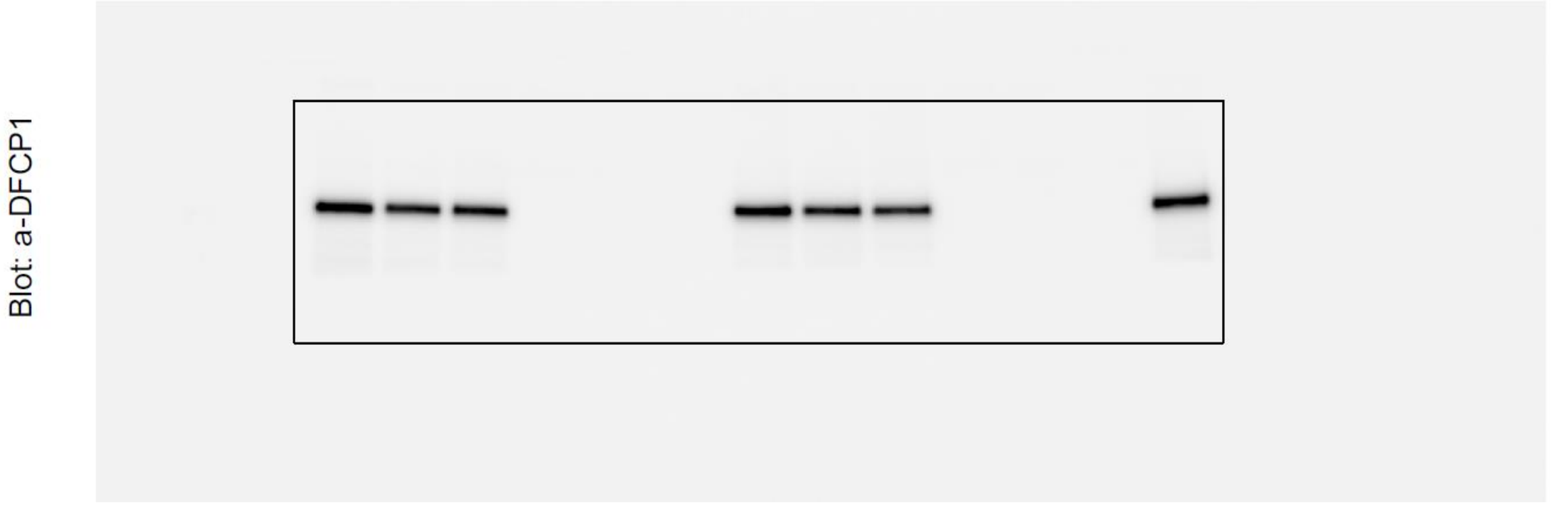

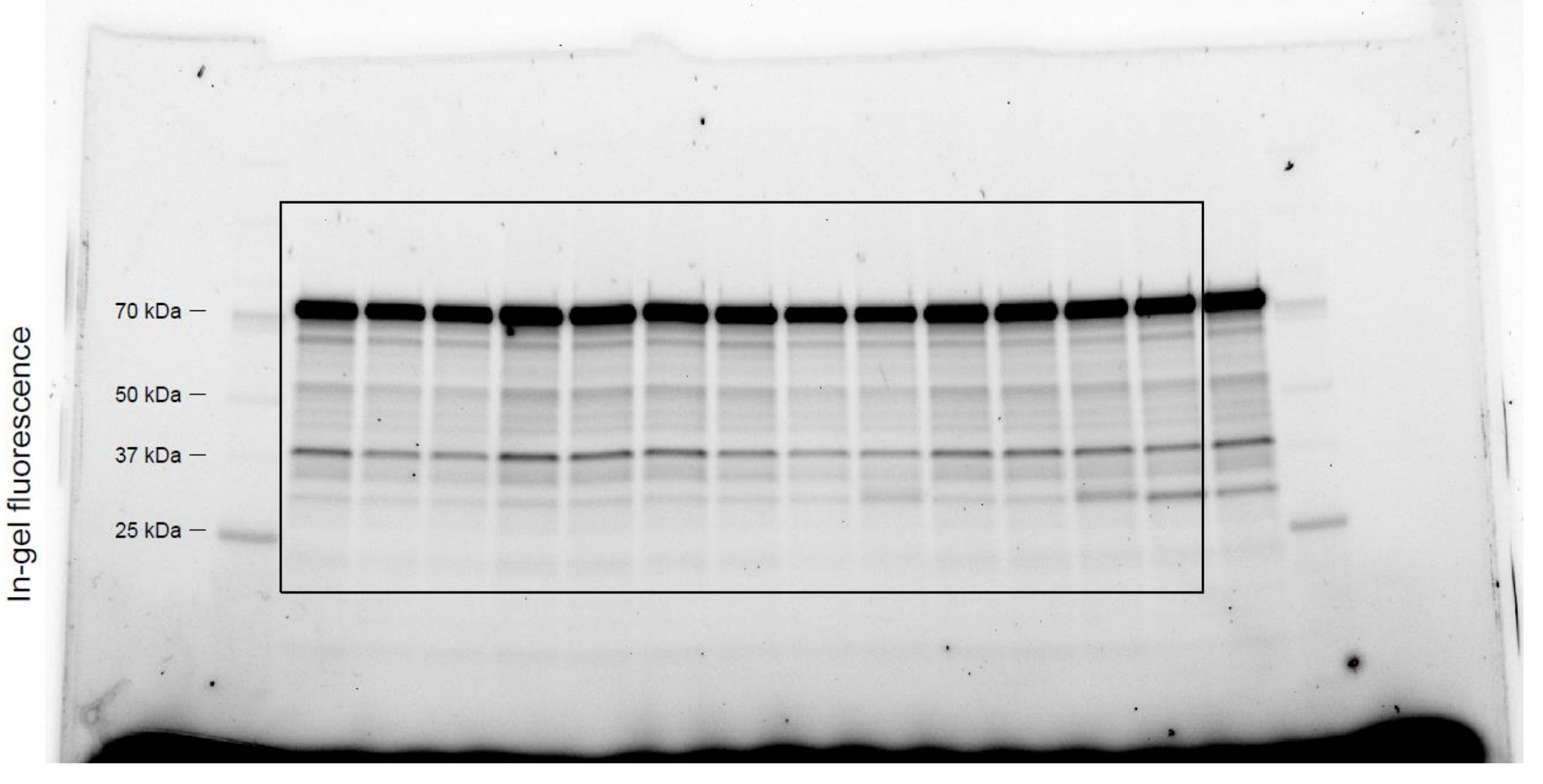

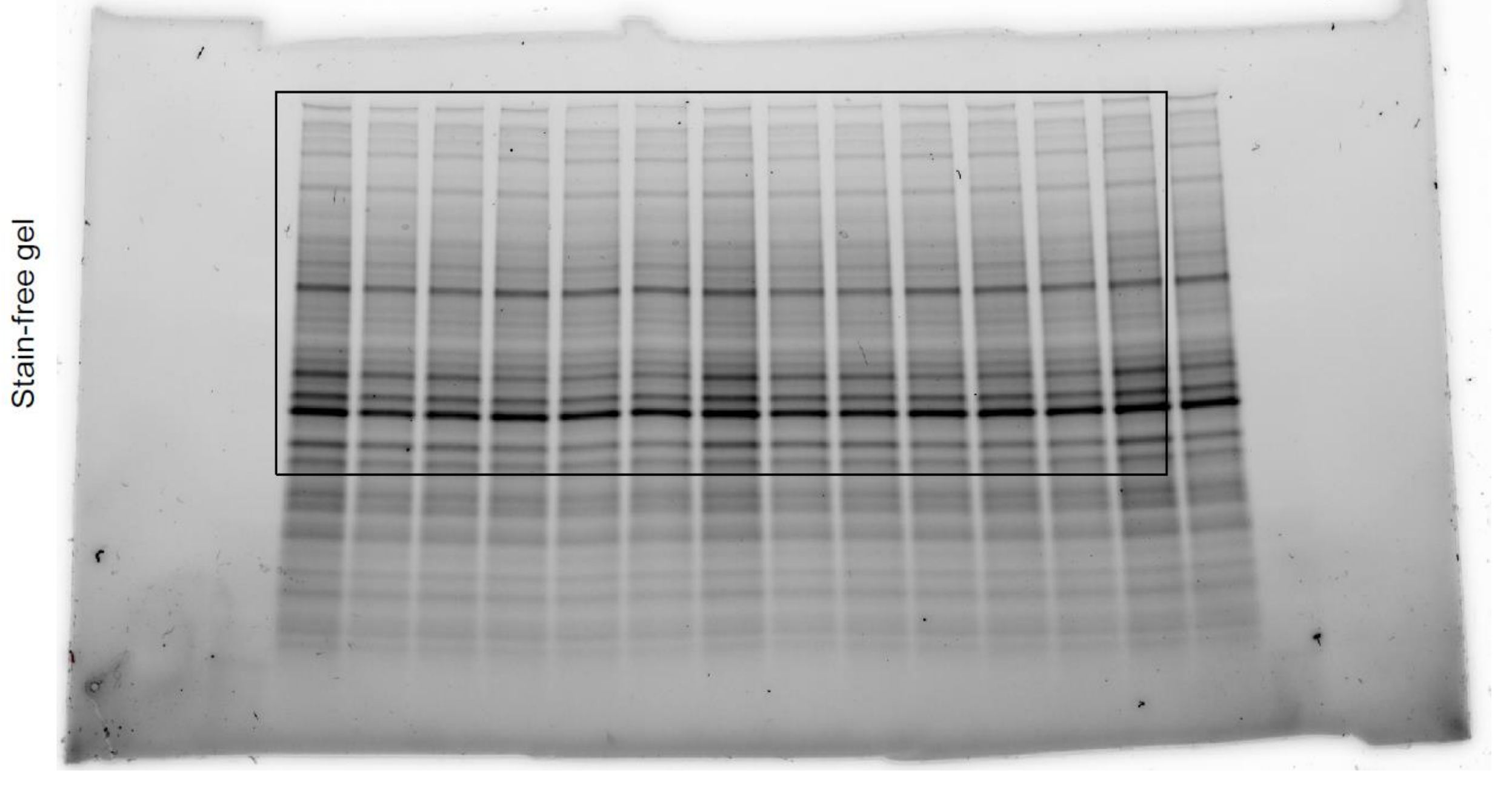

Supplement: Supplementary file 2 [file 41556_2026_2062_MOESM28_ESM.pdf]
